# Unsaturated fatty acids and a prenylated tryptophan derivative from a rare actinomycete of the genus *Couchioplanes*

**DOI:** 10.3762/bjoc.17.203

**Published:** 2021-12-16

**Authors:** Shun Saito, Kanji Indo, Naoya Oku, Hisayuki Komaki, Masashi Kawasaki, Yasuhiro Igarashi

**Affiliations:** 1 Biotechnology Research Center and Department of Biotechnology, Toyama Prefectural University, 5180 Kurokawa, Imizu, Toyama 939-0398, Japan; 2 Department of Biosciences and Informatics, Keio University, 3-14-1 Hiyoshi, Kohoku-ku, Yokohama 223-8522, Japan; 3 Biological Resource Center, National Institute of Technology and Evaluation (NBRC), Kisarazu, Chiba 292-0818, Japan; 4 Center for Liberal Arts and Sciences, Faculty of Engineering, Toyama Prefectural University, 5180 Kurokawa, Imizu, Toyama 939-0398, Japan

**Keywords:** *Couchioplanes*, prenylated tryptophan, rare actinomycete, unsaturated fatty acid

## Abstract

A genome mining survey combined with metabolome analysis of publicly available strains identified *Couchioplanes* sp. RD010705, a strain belonging to an underexplored genus of rare actinomycetes, as a producer of new metabolites. HPLC-DAD-guided fractionation of its fermentation extracts resulted in the isolation of five new methyl-branched unsaturated fatty acids, (2*E*,4*E*)-2,4-dimethyl-2,4-octadienoic acid (**1**), (2*E*,4*E*)-2,4,7-trimethyl-2,4-octadienoic acid (**2**), (*R*)-(−)-phialomustin B (**3**), (2*E*,4*E*)-7-hydroxy-2,4-dimethyl-2,4-octadienoic acid (**4**), (2*E*,4*E*)-7-hydroxy-2,4,7-trimethyl-2,4-octadienoic acid (**5**), and one prenylated tryptophan derivative, 6-(3,3-dimethylallyl)-*N*-acetyl-ʟ-tryptophan (**6**). The enantiomer ratio of **4** was determined to be approximately *S*/*R* = 56:44 by a recursive application of Trost’s chiral anisotropy analysis and chiral HPLC analysis of its methyl ester. Compounds **1**–**5** were weakly inhibitory against *Kocuria rhizophila* at MIC 100 μg/mL and none were cytotoxic against P388 at the same concentration.

## Introduction

Actinomycetes, a subgroup of filamentous Gram-positive bacteria within the class *Actinomycetales*, have provided many important clinical drugs [1–2], agrochemicals [3], food additives [4–5], and biochemical reagents [6–9], and continue to be a core source of bioactive molecules [10]. While most of the actinomycetes-derived compounds have been reported from the genus *Streptomyces* [11], non-*Streptomyces* actinomycetes, commonly referred to as rare actinomycetes [12], are attracting considerable attention as less tapped taxa for drug discovery. Those within the family *Micromonosporaceae*, represented by the second most prolific genus *Micromonospora* following *Streptomyces*, are especially noted, accounting for more than 800 metabolites of actinomycetes origin [11], which include the antiinfective aminoglycoside gentamicin [13], antidiabetic glycoside acarbose [14], glycopeptide antibiotic teicoplanin [15], enediyne antitumor component of drug-antibody conjugate calicheamicin γ_1_^I^ [16], and *Clostridium difficile*-specific macrolide fidaxomicin [17].

At present, only 5 out of 29 valid genera in this family [18] – *Actinoplanes*, *Dactylosporangium*, *Micromonospora*, *Salinispora*, and *Verrucosispora* – have mainly been investigated [11] and the remaining 24 are still untouched or underexplored. However, in silico genome mining identified multiple secondary metabolite biosynthetic gene clusters in selected strains from minor actinomycetes genera, implying their comparable biosynthetic capacities to those of the already proven genera [19]. Encouraged by these reports, we examined the metabolites of *Pseudosporangium* sp. RD062863, a strain available at the culture collection of the Biological Resource Center, National Institute of Technology and Evaluation (NBRC) [20], and discovered a novel cyclopeptide pseudosporamide along with three new oligomycin-class polyketide [21]. In addition, the same approach to the different family (*Pseudonocardiaceae*) yielded mycetoindole, a new class of dehydrotryptophan derivative from *Actinomycetospora* [22].

**Figure 1:**
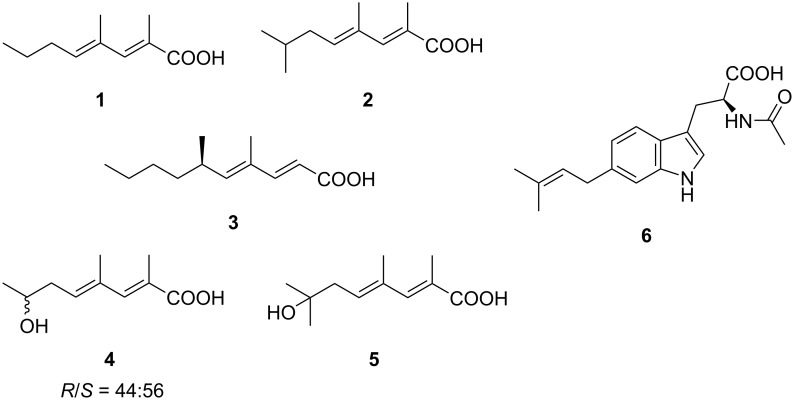
Structures of **1**–**6**.

As part of our continuing studies on the metabolites from underexplored rare actinomycetes, the genus *Couchioplanes*, another minor genus in *Micromonosporaceae* first isolated in 1994 from a sandy soil in Japan [23], was set to be the next target. While the anti-SMASH-assisted genome mining [24] in *C. caeruleus* DSM 43634 revealed approximately 20 secondary metabolite biosynthetic gene clusters, only one compound, heptaene macrolide 67-121C, is known to date [25], leaving room for exploration. Four strains of the same genus*,* available at the NBRC’s culture collection [20], were fermented and their metabolites were analyzed by HPLC-DAD, which detected several prominent peaks from the culture extracts of strain RD010705. Fractionation and purification guided by characteristic UV spectra led to the discovery of five new methyl-branched unsaturated fatty acids, (2*E*,4*E*)-2,4-dimethyl-2,4-octadienoic acid (**1**), (2*E*,4*E*)-2,4,7-trimethyl-2,4-octadienoic acid (**2**), (*R*)-(−)-phialomustin B (**3**), (2*E*,4*E*)-7-hydroxy-2,4-dimethyl-2,4-octadienoic acid (**4**), and (2*E*,4*E*)-7-hydroxy-2,4,7-trimethyl-2,4-octadienoic acid (**5**), as well as one new prenylated tryptophan derivative, 6-(3,3-dimethylallyl)-*N*-acetyl-ʟ-tryptophan (**6**) (Figure 1).

## Results and Discussion

The producing strain RD010705 was shake-cultured in A16 liquid medium at 30 °C for 8 days, and the whole culture was extracted with 1-butanol. The extract (4.4 g from 3 L) was sequentially fractionated by column chromatographies on silica gel and ODS, and the resulting fractions were purified by reverse-phase HPLC to give **1** (5.2 mg), **2** (2.3 mg), **3** (1.0 mg), **4** (6.3 mg), and **5** (8.0 mg).

**Figure 2:**
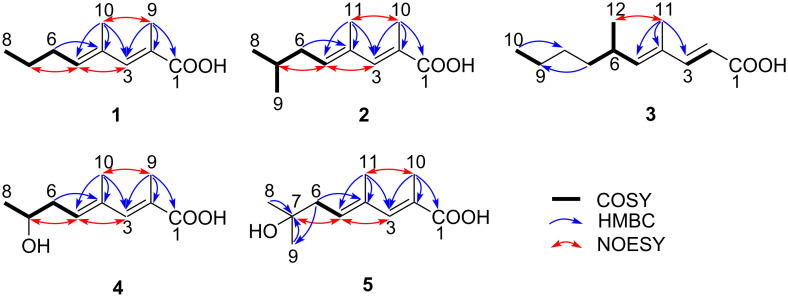
COSY, key HMBC and NOESY correlations of **1**–**5**.

The molecular formula of **1** was determined to be C_10_H_16_O_2_ on the basis of its NMR and HR–ESI–TOFMS data (*m/z* 191.1044 [M + Na]^+^, Δ + 0.1 mmu). Three degrees of unsaturation, calculated from the molecular formula, a UV absorption maximum at 264 nm, and IR absorption bands at 1679 and 2800–3200 cm^−1^, suggested dienone and hydroxy functionalities. The ^1^H, ^13^C, and HSQC data allowed to assign ten carbon signals to one carbonyl carbon (δ_C_ 174.0), two each of non-protonated sp^2^ carbons (δ_C_ 132.5, 123.9), sp^2^ methines (δ_C_ 145.7, 138.5), and sp^3^ methylenes, and three methyls (Table 1 and Table 2). A sequence of COSY correlations was detected from a triplet methyl H-8 to an olefinic methine H-5 via two methylenes H-7 and H-6, which revealed a propyl-substituted olefin moiety (Figure 2). In addition, HMBC correlations (Table 2) from the allylic methyl H-10 to C-5, C-4, and C-3 and from the other allylic methyl H-9 to C-3, C-2, and C-1 elucidated the carbon connectivity from C-5 to C-1, thus completing the planar structure. The double bond geometries were determined to be both *E* based on NOESY correlations between H-3 and H-5 and between H-9 and H-10 (Figure 2). Therefore, **1** was determined to be (2*E*,4*E*)-2,4-dimethyl-2,4-octadienoic acid.

**Table 1:** ^13^C NMR data for **1**–**5** in CDCl_3_.

	**1**	**2**	**3**	**4**	**5**
	
No.	δ_C_, type^a^	δ_C_, type^a^	δ_C_, type^a^	δ_C_, type^a^	δ_C_, type^a^

1	174.0, C	173.9, C	177.1, C	174.1, C	174.1, C
2	123.9, C	124.0, C	115.1, CH	124.8, C	124.8, C
3	145.7, CH	145.7, CH	151.7, CH	144.8, CH	145.2, CH
4	132.5, C	132.9, C	131.5, C	134.7, C	134.9, C
5	138.5, CH	137.4, CH	149.3, CH	133.0, CH	132.8, CH
6	30.7, CH_2_	37.7, CH_2_	33.3, CH	38.2, CH_2_	42.5, CH_2_
7	22.5, CH_2_	28.8, CH	37.0, CH_2_	67.8, CH	71.7, C
8	14.0, CH_3_	22.6, CH_3_^b^	29.8, CH_2_	23.1, CH_3_	29.4, CH_3_^b^
9	13.8, CH_3_	22.6, CH_3_^b^	22.9, CH_2_	13.7, CH_3_	29.4, CH_3_^b^
10	16.3, CH_3_	13.9, CH_3_	14.2, CH_3_	16.5, CH_3_	13.9, CH_3_
11		16.4, CH_3_	12.4, CH_3_		16.6, CH_3_
12			20.6, CH_3_		

^a^Recorded at 125 MHz (reference δ_C_ 77.2). ^b^Overlapping signals.

**Table 2:** ^1^H NMR data for **1**–**5** in CDCl_3_.

	**1**	**2**	**3**	**4**	**5**
	
No.	δ_H_, mult (*J* in Hz)^a^	δ_H_, mult (*J* in Hz)^a^	δ_H_, mult (*J* in Hz)^a^	δ_H_, mult (*J* in Hz)^a^	δ_H_, mult (*J* in Hz)^a^

1					
2			5.79, d (15.2)		
3	7.26, s	7.26, s	7.35, d (14.7)	7.26, s	7.28, s
4					
5	5.70, t (7.2)	5.72, t (7.5)	5.68, d (9.1)	5.72, t (7.3)	5.79, t (7.6)
6	2.14, q (7.5)	2.05, t (7.1)	2.51, m	2.35, m	2.36, d (7.9)
7	1.45, sex (7.4)	1.71, m	1.27, m^b^	3.94, sex (6.2)	
			1.36, m		
8	0.94, t (7.4)	0.93, d (6.7)^b^	1.21–1.26, m	1.24, dd (6.2)	1.27, s^b^
9	2.02, s	0.93, d (6.7)^b^	1.23–1.27, m^b^	2.02, s	1.27, s^b^
10	1.86, s	2.03, s	0.87, t (7.3)	1.89, s	2.01, s
11		1.86, s	1.77, s		1.86, s
12			0.97, d (6.6)		

No.	HMBC^c^	HMBC^c^	HMBC^c^	HMBC^c^	HMBC^c^

1					
2					
3	1, 2, 5, 9, 10	1, 2, 5, 10, 11		1, 2, 5, 9, 10	1, 2, 5, 10, 11
4					
5	3, 6, 7, 10	3, 6, 7, 11		3, 6, 7, 10	3, 6, 7, 11
6	4, 5, 7, 8	4, 5, 7, 8, 9		4, 5, 7, 8	4, 5, 7, 8, 9
7	5, 6, 8	5, 6, 8, 9	8, 9	5, 6, 8	
8	6, 7	6, 7, 9	7, 9	6, 7	6, 7, 9
9	1, 2, 3	6, 7, 8	8, 10	1, 2, 3	6, 7, 8
10	3, 4, 5	1, 2, 3	8, 9	3, 4, 5	1, 2, 3
11		3, 4, 5	3, 4, 5		3, 4, 5
12			5, 6, 7		

^a^Recorded at 500 MHz (reference δ_H_ 7.26). ^b^Overlapping signals. ^c^From proton to indicated carbon(s).

The ^1^H NMR spectrum of **2** was similar to those of **1** in overall (Table 1 and Table 2). One major difference was a replacement of the triplet methyl peak in **1** by a six proton-equivalent doublet methyl peak, which suggested an isopropyl-terminated structure. This was proven by a COSY correlation between this methyl proton (H-8/H-9) and a methine proton (H-7) and HMBC correlations from H-8/H-9 to the methine carbon (C-7) and the allylic methylene carbon (C-6). The deduced structure was consistent with a molecular formula C_11_H_18_O_2_ established by HR–ESI–TOFMS analysis (*m/z* 205.1202 [M + Na]^+^, Δ + 0.3 mmu), which is larger than **1** by one methylene. NOESY correlations surrounding the double bonds was the same to those observed for **1** (Figure 2), thus concluding **2** to be (2*E*,4*E*)-2,4,7-trimethyl-2,4-octadienoic acid.

Compound **3** has a molecular formula of C_12_H_20_O_2_ (*m/z* 219.1356 [M + Na]^+^, Δ 0.0 mmu), which is larger by C_2_H_4_ than **1**. Analysis of a COSY spectrum identified a 1,2-disubstituted (*E*)-olefin fragment H-2/H-3 (^3^*J*_H-H_ = 15.0 Hz), a four-carbon fragment containing a methyl-substituted olefinic methine H-5/H-6/(H-12)/H-7, and an ethyl group H-9/H-10 (Figure 2, Table 1 and Table 2). The connectivity of the former two fragments were intervened by an sp^2^ quaternary carbon (C-4) substituted by an allylic methyl group (H-11) based on HMBC correlations from H-11 to C-3, C-4, and C-5. Another intervention by a methylene unit (C-8) to connect the second and third fragments was supported by HMBC correlations from H-7 to C-9, H-8 to C-6, and H-10 to C-8, thus completing an alkyl chain part. The remaining atomic composition was CHO_2_, and despite the lack of evidentiary HMBC correlations, placing a carboxylic acid functionality at the open end (C-2) was reasonable in consideration of the chemical shift of the unused carbon (δ_H_ 177.1, C-1) and the molecular formula. A NOESY correlation between H-11 and H-12 supported an *E*-configuration for the C-4/C-5 double bond. The established planar structure was identical to that of a fungal metabolite phialomustin B [26], for which specific rotation, enumerated ^1^H and ^13^C NMR data, and high-resolution ESIMS data were presented. While an *S*-configuration was assigned for phialomustin B based on its positive specific rotation ([α]_D_^25^ +55.5, *c* 1.5, CHCl_3_) in comparison with those of synthetic standards [26], opposite negative signs in CHCl_3_ and MeOH ([α]_D_^27^ –12, *c* 0.035, CHCl_3_; [α]_D_^25^ –76.0, *c* 0.05, MeOH), though the rotatory power in CHCl_3_ not as large as expected presumably due to the sample scarcity, supported an *R*-configuration. Therefore, **3** was speculated to be (*R*)-(−)-phialomustin B.

**Figure 3:**
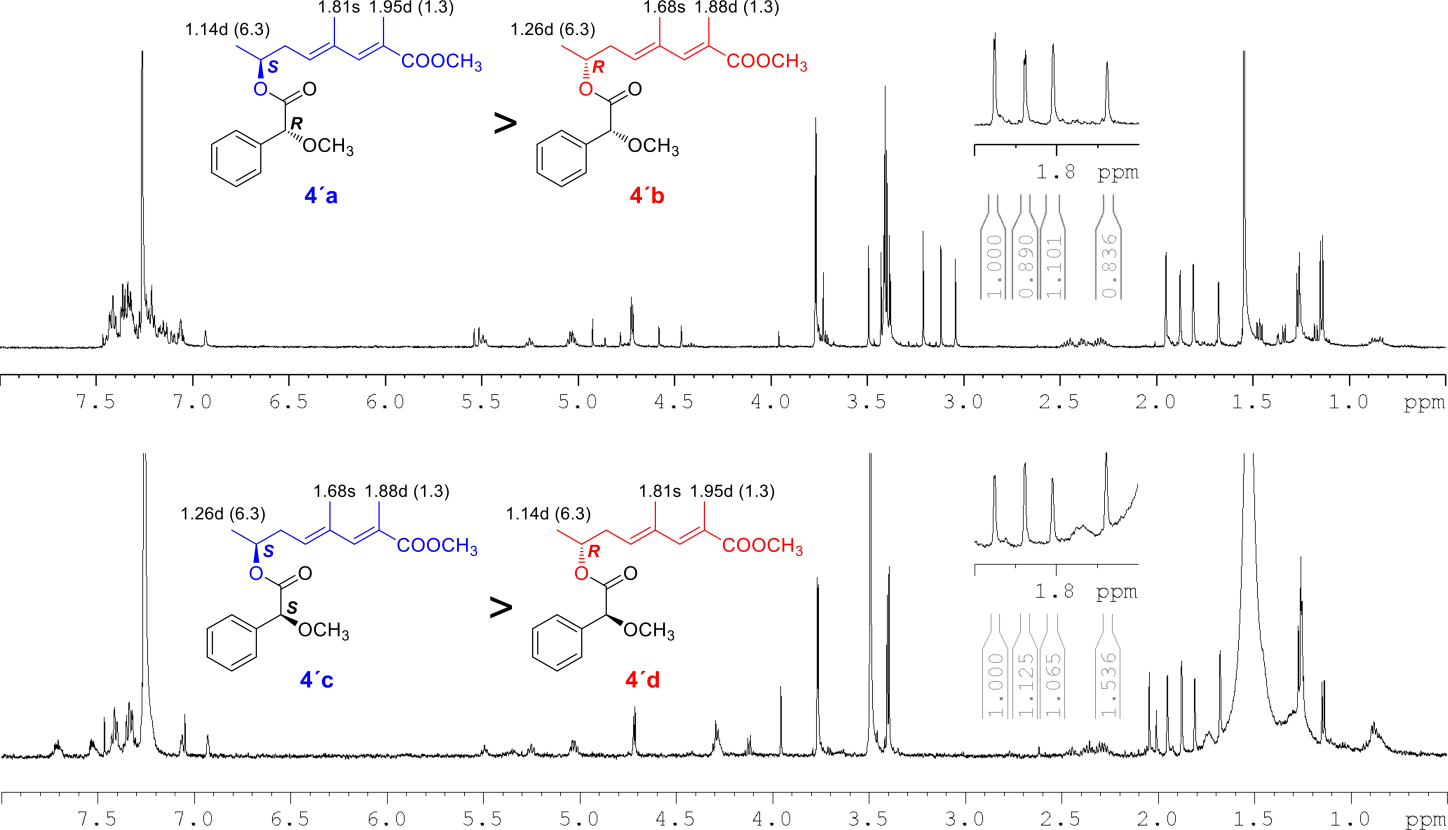
^1^H NMR spectra and partial chemical shift assignments for MPA esters **4'a**–**4'd**. Upper: (*R*)-MPA acylation products, Lower: (*S*)-MPA acylation products.

**Figure 4:**
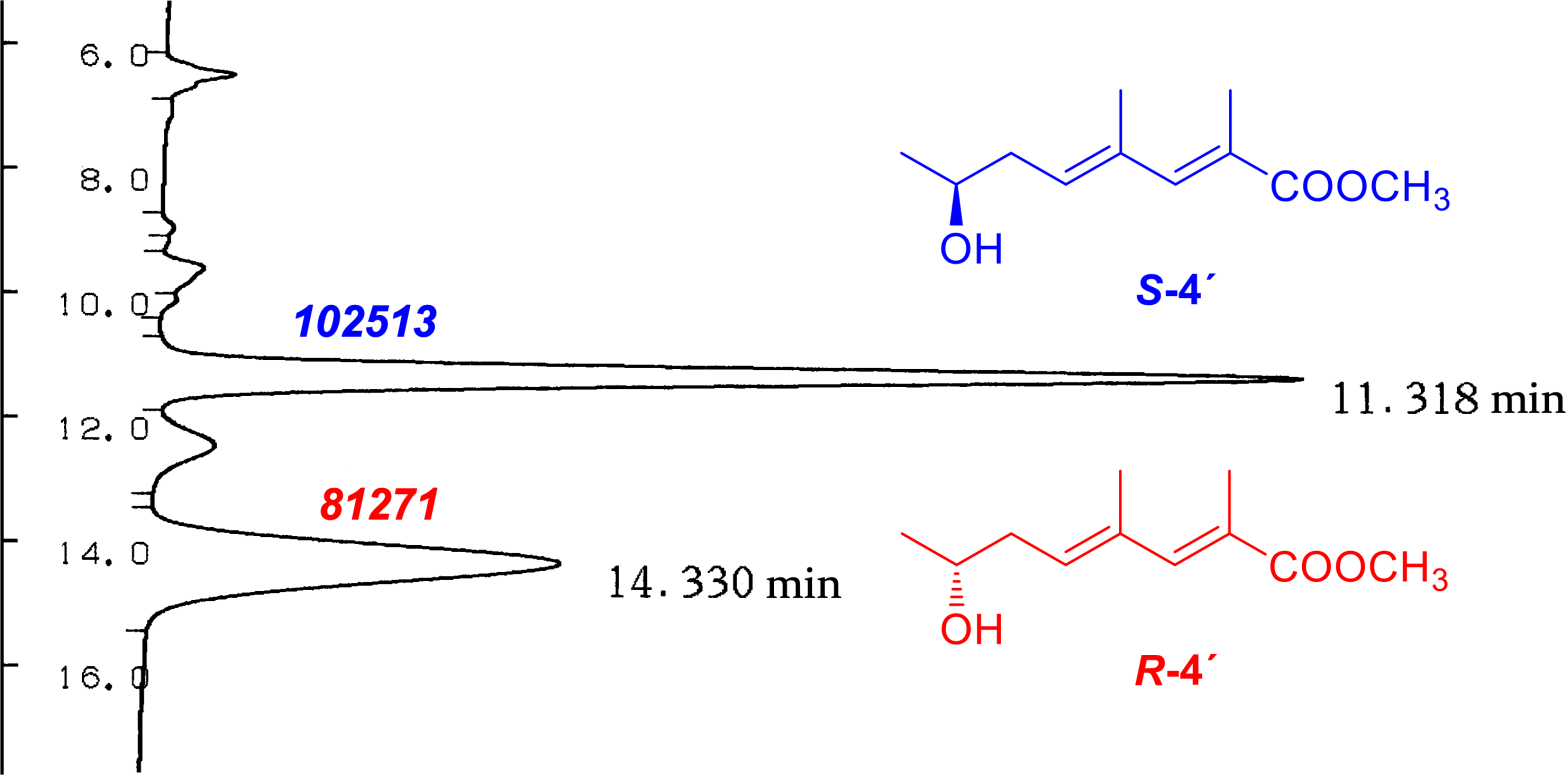
Chiral phase HPLC analysis of methyl ester **4'**. Italicized numbers indicate peak areas.

The molecular formula of **4**, determined to be C_10_H_16_O_3_ on the basis of HR-ESITOF-MS data (*m/z* 207.0992 [M + Na]^+^, Δ 0.0 mmu), was one-oxygen larger than that of **1**. The ^1^H NMR spectrum was mostly similar to those of **1** (Table 1 and Table 2), except for the presence of an oxymethine resonance (δ_H_ 3.96/δ_C_ 67.8) in place of the shielded methylene at C-7, which suggested hydroxylation on the same carbon. This was supported by COSY correlations establishing the connectivity from H-5 to H-8, and completely the same HMBC and NOESY correlations for the remaining part to those observed for **1** and **2** (Figure 2). To address the absolute configuration, **4** was esterified with TMS-diazomethane and the resulting methyl ester **4'** was acylated with (*R*)- or (*S*)-α-methoxyphenylacetic acid (MPA). To our surprise, however, ^1^H NMR spectra of both the acylation products were substantially the same but contained several duplicated signals with contrasting peak intensities, indicating **4** to be an enantiomeric mixture of unequal quantities (Figure 3). Indeed, two ^1^H resonance sets with slightly different chemical shifts were obtained, which were assigned to be a pair of MPA-acylated diastereomers (**4'a** and **4'b**; **4'c** and **4'd**) by careful interpretation of a COSY spectrum of the (*R*)-MPA acylation product mixture (Supporting Information File 1, Figure S25). Furthermore, the slightly excessive **4'a** and **4'c** were identified to be MPA derivatives of (*S*)-**4**, and hence less excessive **4'b** and **4'd** to be those of (*R*)-**4**, by recursive application of Trost’s chiral anisotropy rule to the ^1^H chemical shift differences between the diastereomer pairs. The enantiomer ratio of **4** was estimated to be *S*/*R* = 56:44 or near by chiral phase HPLC analysis of **4'** on a cellulose tribenzoate-coated silica gel column (Figure 4). Thus, **4** was concluded to be an enantiomeric mixture of (2*E*,4*E*)-7-hydroxy-2,4-dimethyl-2,4-octadienoic acid with an approximate enantiomer ratio of *S*/*R* = 56:44.

The ^1^H and ^13^C NMR spectra of **5** were quite similar to those of **4** (Table 1 and Table 2), except for the lack of the oxymethine proton resonance and replacement of the doublet methyl resonance by a singlet signal with a six-proton intensity. These differences, together with a one methylene-larger molecular formula (C_11_H_18_O_3_), suggested a methyl substitution at the carbinol carbon (δ_C_ 71.7, C-7). This was verified by HMBC correlations from the singlet methyl proton (H-8/H-9) to the carbinol carbon. Thus, **5** was identified to be (2*E*,4*E*)-7-hydroxy-2,4,7-trimethyl-2,4-octadienoic acid.

The same strain also produced another new compound, 6-(3,3-dimethylallyl)-*N*-acetyl-ʟ-tryptophan (**6**), when fermented in modified V22, a different medium. This compound was isolated by the same sequence of chromatographies as above, yielding 45 mg from a 6 L culture. The molecular formula was determined to be C_18_H_22_N_2_O_3_ based on its NMR and HR–ESI–TOFMS data (*m/z* 313.1556 [M – H]^–^, Δ – 0.2 mmu), corresponding to nine degrees of unsaturation. The UV spectrum, exhibiting the absorption maxima at 229 and 282 nm, was typical of an indole functionality. The IR absorption bands at 3310 and 1656 cm^–1^ suggested the presence of OH/NH and carbonyl groups. ^1^H, ^13^C, and HSQC spectra (Table 3) revealed the composition of this molecule to be two shielded carbonyls, five other sp^2^ nonprotonated carbons, five sp^2^ methines, one sp^3^ methine, two sp^3^ methylenes, three singlet methyls, and two amino protons. The remaining OH group should be a part of a carboxylic acid functionality considering the lack of oxygenated carbons besides carbonyls, and two degrees of unsaturation, not accounted for by double bonds, were consistent with the indole ring.

**Table 3:** NMR data for **6** in CDCl_3_.

No.	δ_C_,^a^ type	δ_H_, mult (*J* in Hz)^b^	HMBC^c^

1	NH	8.41, s	2, 3, 3a, 7a
2	122.9, CH	6.91, s	3, 3a, 7a
3	109.9, C		
3a	126.3, C		
4	118.6, CH	7.45, d (8.2)	3, 3a, 6
5	120.8, CH	6.92, d (8.3)	3a, 7, 1'
6	136.0, C		
7	110.7, CH	7.11, s	3a, 5, 6, 1'
7a	136.7, C		
8	27.4, CH_2_	3.29, m	2, 3, 3a, 9, 10
9	53.4, CH	4.87, m	3, 8, 10, 11
10	174.3, C		
11	NH	6.26, d (6.9)	9, 10, 12
12	170.7, C		
13	23.2, CH_3_	1.91, s	12
1'	34.7, CH_2_	3.41, d (6.9)	5, 6, 7, 2', 3'
2'	124.1, CH	5.35, t (7.5)	1', 4', 5'
3'	132.1, C		
4'	18.0, CH_3_	1.73, s^d^	2', 3', 5'
5'	25.9, CH_3_	1.73, s^d^	2', 3', 4'

^a^Recorded at 125 MHz (reference δ_C_ 77.2). ^b^Recorded at 500 MHz (reference δ_H_ 7.26). ^c^From proton to indicated carbon(s). ^d^Overlapping signals.

**Figure 5:**
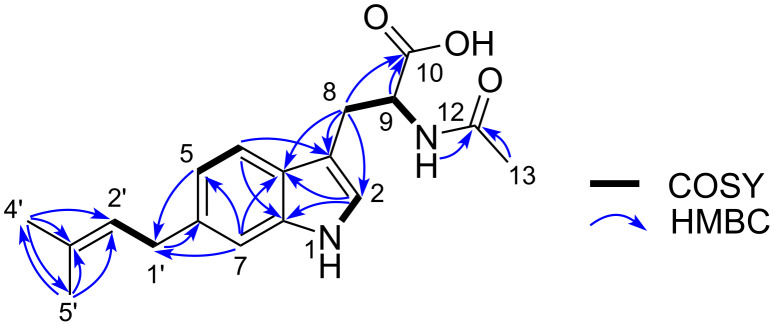
COSY and key HMBC correlations observed for **6**.

**Figure 6:**
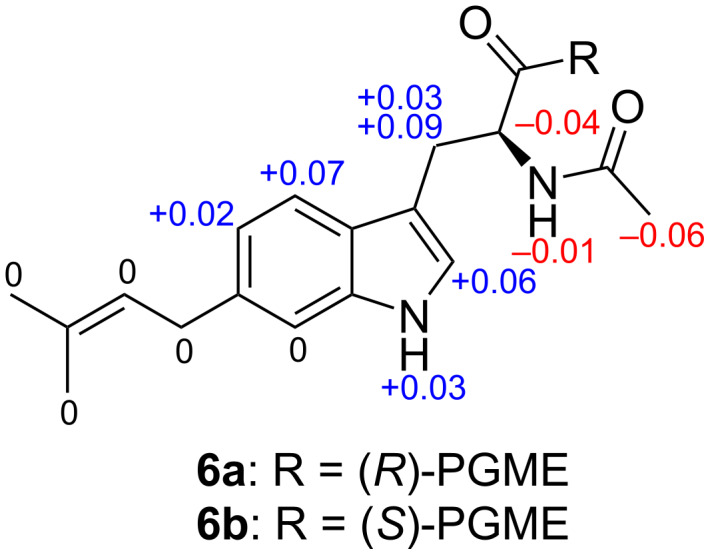
Δδ_H_(*S-R*) values in ppm calculated from PGME amides **6a** and **6b**.

As expected, assembling the above components by COSY and HMBC correlations established 6-prenylated *N*-acetyltryptophan (Figure 5). The *N*-acetylation was evident from HMBC correlations from the amide (NH-11) and acetyl methyl protons (H-13) to the amide carbon (C-12), while prenylation at C-6 was supported by HMBC correlations from H-5 and H-7 to C-1' and from H-1' to C-6. The absolute configuration was determined by chiral anisotropy analysis after derivatization with each of the phenylglycine methyl ester (PGME) enantiomers [27], which gave positive Δδ_H_(*S*-*R*) values for NH-1, H-2, H-4, H-5 and H-8 and negative values for H-9, NH-11 and H-13 (Figure 6). Thus, an *S*-configuration, corresponding to an ʟ-chirality for tryptophan, was assigned.

Compounds **1**–**6** were not cytotoxic against P388 murine leukemia cells (IC_50_ > 100 μM), nor antimicrobial against five bacteria, *Bacillus subtilis*, *Staphylococcus aureus*, *Ralstonia solanacearum*, *Rhizobium radiobacter* and *Escherichia coli*, or a yeast *Candida albicans* (MIC > 100 μg/mL). Compounds **1**–**5** were marginally active against *Kocuria rhizophila* (MIC = 100 μg/mL).

## Conclusion

**Figure 7:**
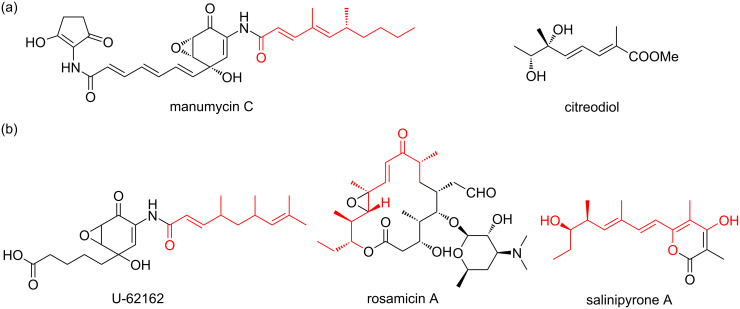
Selected examples of the related compounds derived from the strains in the genus *Streptomyces* (a) and the family *Micromonosporaceae* (b).

In this study, five unsaturated fatty acids (**1**–**5**) and one prenylated tryptophan derivative (**6**) were isolated as new natural products from *Couchioplanes* sp. RD010705. The α,γ-dimethyl-α,γ-dienoyl C_8_ motif in **1**, **2**, **4**, and **5** is only precedented by 64p-B (2,4-dimethyl-2,4-octadienamide) produced by manumycin-producing *Streptomyces*, though the physicochemical properties of which is yet to be disclosed [28]. The γ,ε-dimethyl-α,γ-dienoyl motif in **3** is seen in many antibiotics but that with a C_10_ chain length is only precedented by manumycin C [29], TMC-1 C [30], and phialomustins [26] (Figure 7). In the family *Micromonosporaceae*, a strain belonging to the genus *Plantactinospora* is known to produce U-62162, a manumycin-type metabolite with a methyl-branched C_9_ unsaturated acyl chain [31]. Moreover, salinipyrones, produced by a *Salinispora* strain, were shown to be biosynthetic byproducts of the rosamicin polyketide synthase [32]. Though not a result from *Micromonosporaceae*, another example of truncated polyketides is citreodiol, a similarly methyl-branched unsaturated fatty acid ester, which is produced by type I polyketide synthase in a *Streptomyces* strain by a heterologous expression experiment [33]. These facts suggest that **1**–**5** could be byproducts from the biosynthesis of larger polyketides, but further investigation is necessary for their biosynthesis. Prenylated indoles are widely distributed among bacteria, fungi and plants, and all seven positions are subject of prenylation except for the bridgehead carbons [34]. Compound **6** is the acetylated derivative of 6-(3,3-dimethylallyl)-ʟ-tryptophan from *Streptomyces* sp. SN-593 [35]. Further chemical exploration on the genus *Couchioplanes* will disclose its actual biosynthetic capacity in secondary metabolism.

## Experimental

### General experimental procedures

Optical rotations were measured using a JASCO P-1030 polarimeter. UV spectra were recorded on a Shimadzu UV-1800 UV–vis spectrophotometer. IR spectra were measured on a PerkinElmer Spectrum 100. NMR spectra were obtained on a Bruker AVANCE II 500 or AVANCE NEO 500 spectrometer in DMSO-*d*_6_ or CDCl_3_, and referenced to the residual solvent signals (δ_H_ 2.50, δ_C_ 39.5 for DMSO-*d*_6_; δ_H_ 7.26, δ_C_ 77.2 for CDCl_3_). HR–ESI–TOFMS spectra were recorded on a Bruker micrOTOF spectrometer. Silica gel 60 (spherical) (Kanto Chemical Co., Inc.) was used for silica gel column chromatography. Cosmosil 75C18-PREP (Nacalai Tesque, Inc.) was used for ODS column chromatography. Routine HPLC separations were performed on an Agilent HP1200 system, and chiral HPLC analysis was done on a Shimadzu Prominence UFLC system composed of a DGU-20A_3R_ degasser, an SPD-20A UV detector, and an LC-20AD pump, which is connected to a C-R8A Chromatopac data processor.

#### Microorganism

Strain RD010705 was obtained from NBRC. The strain was identified as a member of the genus *Couchioplanes* on the basis of 98.2% similarity in the 16S rRNA gene sequence (1418 nucleotides; DDBJ accession number LC512746) to *Couchioplanes caeruleus* strain DSM 44103^T^ (accession number NR_026295.1).

#### Fermentation

Strain RD010705, grown on half-strength ISP medium 2 consisting of yeast extract 0.2%, malt extract 0.5%, and glucose 0.2% (pH 7.3) solidified by agar 2%, was inoculated into test tubes (inner diameter, 15 mm; length 16.5 cm) each containing 5 mL of YG seed medium consisting of glucose 1% and yeast extract 1% (pH 7.0). The tubes were shaken at 260 strokes/min at 28 °C for 3 days (TC-500R, Takasaki Scientific Instruments Corp.). One-mL aliquots of the resulting seed cultures were transferred into 500 mL Erlenmeyer flasks each containing 100 mL of production medium and the flasks were fermented on a rotary shaker (TB98, Takasaki Scientific Instruments Corp.) operated at 120 rpm at 30 °C for 8 days.

### Isolation of compounds **1**–**5**

Compounds **1**–**5** were obtained from a culture fermented in A16 production medium with a composition of glucose 2%, Pharmamedia^®^ (Traders Protein, Memphis, TN, USA) 1%, and CaCO_3_ 0.5%. The pH of the medium was adjusted to 7.0 before autoclaving. At the end of fermentation, 100 mL of 1-butanol was added to each flask, and the flasks were allowed to shake for 1 h. The mixture was centrifuged at 6,000 rpm for 10 min and the organic layer was separated from the aqueous layer containing the mycelium. The organic layer was concentrated in vacuo to give 4.4 g of an extract from a 3 L culture. The extract was subjected to silica gel column chromatography eluted with a step gradient of CHCl_3_/MeOH mixture solvent (1:0, 20:1, 10:1, 4:1, 2:1, 1:1, and 0:1 v/v). The sixth fraction eluted with CHCl_3_/MeOH (1:1) was evaporated to dryness and the oily residue (429 mg) was chromatographed on ODS eluted with a step gradient of MeCN/0.1% HCO_2_H solutions (2:8, 3:7, 4:6, 5:5, 6:4, 7:3, and 8:2 v/v). The second, third, and fifth fractions were purified by HPLC on an Nacalai Tesque Cosmosil 5C18-AR-II packed column (10 × 250 mm) eluted with linear gradient elution of MeCN from 15 to 85% over 35 min in 0.1% HCO_2_H at 4 mL/min to give (2*E*,4*E*)-7-hydroxy-2,4-dimethyl-2,4-octadienoic acid (**4**, 6.3 mg, *t*_R_ = 12.9 min), (2*E*,4*E*)-7-hydroxy-2,4,7-trimethyl-2,4-octadienoic acid (**5**, 8.0 mg, *t*_R_ = 13.5 min), and (2*E*,4*E*)-2,4-dimethyl-2,4-octadienoic acid (**1**, 5.2 mg, *t*_R_ = 30.0 min), respectively. The fifth fraction (424 mg) eluted by CHCl_3_/MeOH (2:1) from the silica gel column was similarly fractionated by ODS column chromatography. The sixth fraction was purified by the HPLC operation to give (2*E*,4*E*)-2,4,7-trimethyl-2,4-octadienoic acid (**2**, 2.3 mg, *t*_R_ = 29.1 min) and (*R*)-(−)-phialomustin B (**3**, 1.0 mg, *t*_R_ = 32.1 min).

(2*E*,4*E*)-2,4-Dimethyl-2,4-octadienoic acid (**1**): yellow oil; UV (MeOH) λ_max_ (log ε) 264 (4.08) nm; IR (ATR) ν_max_: 2959, 2610, 1679, 1622, 1417, 1269, 1138, 1012, 926 cm^–1^; ^1^H and ^13^C NMR data, see Table 1 and Table 2; HR–ESI–TOFMS (*m/z*): [M + Na]^+^ calcd for C_10_H_16_NaO_2_, 191.1043; found, 191.1044.

(2*E*,4*E*)-2,4,7-Trimethyl-2,4-octadienoic acid (**2**): yellow oil; UV (MeOH) λ_max_ (log ε) 260 (4.04) nm; IR (ATR) ν_max_: 2957, 1681, 1622, 1417, 1269, 1137 cm^–1^; ^1^H and ^13^C NMR data, see Table 1 and Table 2; HR–ESI–TOFMS (*m/z*): [M + Na]^+^ calcd for C_11_H_18_NaO_2_, 205.1199; found, 205.1202.

(*R*)-(−)-Phialomustin B (**3**): yellow oil; [α]_D_^27^ −12 (*c* 0.035, CHCl_3_), [α]_D_^22^ −76 (*c* 0.05, MeOH); UV (MeOH) λ_max_ (log ε) 256 (4.19) nm; IR (ATR) ν_max_: 2958, 2956, 1686, 1620, 1279, 982 cm^–1^; ^1^H and ^13^C NMR data, see Table 1 and Table 2; HR–ESI–TOFMS (*m/z*): [M + Na]^+^ calcd for C_12_H_20_NaO_2_, 219.1356; found, 219.1356.

(2*E*,4*E*)-7-Hydroxy-2,4-dimethyl-2,4-octadienoic acid (**4**), *R*/*S* = 44:56: yellow oil; [α]_D_^22^ −2.6 (*c* 0.30, MeOH); UV (MeOH) λ_max_ (log *ε*) 259 (4.20) nm; IR (ATR) ν_max_: 3356, 2969, 2929, 1680, 1623, 1266, 1125 cm^–1^; ^1^H and ^13^C NMR data, see Table 1 and Table 2; HR–ESI–TOFMS (*m/z*): [M + Na]^+^ calcd for C_10_H_16_NaO_3_, 207.0992; found, 207.0992.

(2*E*,4*E*)-7-Hydroxy-2,4,7-trimethyl-2,4-octadienoic acid (**5**): yellow oil; UV (MeOH) λ_max_ (log ε) 258 (3.94) nm; IR (ATR) ν_max_: 3365, 2970, 1681, 1623, 1266, 1130 cm^–1^; ^1^H and ^13^C NMR data, see Table 1 and Table 2; HR–ESI–TOFMS (*m/z*): [M + Na]^+^ calcd for C_11_H_18_NaO_3_, 221.1148; found, 221.1149.

### Isolation of **6**

Compound **6** was obtained from a culture fermented in modified V22 production medium with a composition of soluble starch 1%, glucose 0.5%, NZ Amine, Type A 0.3%, yeast extract 0.2%, Tryptone 0.5% K_2_HPO_4_ 0.1%, MgSO_4_·7H_2_O 0.05%, and CaCO_3_ 0.3%. The pH of the medium was adjusted to 7.0 before autoclaving. At the end of fermentation, 100 mL of 1-butanol was added to each flask, and the flasks were allowed to shake for 1 h. The mixture was centrifuged at 6,000 rpm for 10 min and the organic layer was separated from the aqueous layer containing the mycelium. The organic layer was concentrated in vacuo to give 5.2 g of an extract from a 6 L culture. The crude extract was chromatographed on a silica gel column similarly as above and the sixth fraction was fractionated by ODS column chromatography with a gradient of MeCN/0.1% HCO_2_H solution (2:8, 3:7, 4:6, 5:5, 6:4, 7:3, and 8:2 v/v). The fraction 4 (5:5) was evaporated, and purified by preparative HPLC operated at the same conditions as above to give 6-(3,3-dimethylallyl)-*N*-acetyl-ʟ-tryptophan (**6**, 45 mg, *t*_R_ = 10.4 min).

6-(3,3-Dimethylallyl)-*N*-acetyl-ʟ-tryptophan (**6**): yellow oil; [α]_D_^22^ +15 (*c* 1.0, MeOH); UV (MeOH) λ_max_ (log ε) 229 (4.59), 282 (4.05) nm; IR (ATR) ν_max_: 3310, 2916, 1724, 1656, 1627, 1548, 1453, 1223, 806 cm^–1^; ^1^H and ^13^C NMR data, see Table 3; HR–ESI–TOFMS (*m/z*): [M − H]^−^ calcd for C_18_H_21_N_2_O_3_, 313.1558; found, 313.1556.

### Preparation of methyl ester **4'**

To a solution of **4** (0.5 mg, 2.7 μmol) in CHCl_3_/MeOH (25 μL each) was added a solution of TMS-diazomethane in *n*-hexane (2.0 M, 25 μL) at room temperature. After stirring for 15 min, the reaction mixture was concentrated to dryness to give methyl ester **4'** (0.2 mg).

Methyl ester **4'**: ^1^H NMR (500 MHz, CDCl_3_) δ 3.76 (s, 3H, -CO_2_C*H_3_*), 7.14 (brs 1H, H3), 5.66 (t, *J* = 7.4 Hz, 1H, H5), 2.33 (m, 2H, H6), 3.92 (m, 1H, H7), 1.24 (d, *J* = 6.4 Hz, 3H, H8), 2.01 (d, *J* = 2.0 Hz, 3H, H9), 1.87 (s, 3H, H10).

### Preparation of (*R*)- and (*S*)-MPA esters **4'a**–**d**

To a solution of **4'** (0.2 mg, 1.0 μmol), (*R*)-MPA (2.0 mg, 12 μmol), and *N*,*N*-dimethyl-4-aminopyridine (3.9 mg, 32 μmol) in dichloromethane (200 μL) were added *N*,*N’*-diisopropylcarbodiimide (3 μL). After stirring for 1 h at room temperature, ice-water was poured into the reaction mixture, which was then extracted with EtOAc. After evaporation of the solvent, the residue was purified by preparative silica gel thin-layer chromatography (Kieselgel 60F_254_; Merck Co.) developed by a mixture of CHCl_3_/MeOH (5:1) to give (*R*)-MPA esters **4'a** and **4'b** with a slight excess yield of the former. A recursive application of Trost’s chiral anisotropy analysis allowed to identify **4'a** to be (*R*)-MPA ester of *S*- and **4'b** to be that of *R*-enantiomers, respectively.

(*R*)-MPA ester of *S*-enantiomer **4'a**: ^1^H NMR (500 MHz, CDCl_3_) δ 3.77 (s, 3H, -CO_2_C*H_3_*), 7.07 (brs 3H, H3), 5.49 (t, *J* = 7.7 Hz, 1H, H5), 2.37 (m, 1H, H6a), 2.45 (m, 1H, H6b), 5.03 (m, 1H, H7), 1.14 (d, *J* = 6.3 Hz, 3H, H8), 1.95 (d, *J* = 1.3 Hz, 3H, H9), 1.81 (s, 3H, H10), 3.405 (s, 3H, MPA-OC*H_3_*), 4.72 (s, 1H, MPA-Cα*H*).

(*R*)-MPA ester of *R*-enantiomer **4'b**: ^1^H NMR (500 MHz, CDCl_3_) δ 3.76 (s, 3H, -CO_2_C*H_3_*), 6.93 (brs 3H, H3), 5.26 (t, *J* = 7.7 Hz, 1H, H5), 2.27 (m, 1H, H6a), 2.31 (m, 1H, H6b), 5.03 (m, 1H, H7), 1.26 (d, *J* = 6.3 Hz, 3H, H8), 1.88 (d, *J* = 1.3 Hz, 3H, H9), 1.68 (s, 3H, H10), 3.396 (s, 3H, MPA-OC*H_3_*), 4.71 (s, 1H, MPA-Cα*H*).

In the same manner as described for the preparation of **4'a** and **4'b**, a diastereomeric mixture of (*S*)-MPA esters **4'c** and **4'd** was prepared from **4'** and (*S*)-MPA.

(*S*)-MPA ester of 7*S* enantiomer **4'c**: ^1^H NMR data was identical to those of **4'b**.

(*S*)-MPA ester of 7*R* enantiomer **4'd**: ^1^H NMR data was identical to those of **4'a**.

### Chiral HPLC analysis

A 0.2 μL-portion of **4'**, dissolved in iPrOH, was injected into a cellulose tribenzoate-coated silica gel column (CHIRALCEL OB-H, 4.6 mm × 250 mm, Daicel Co.) eluted with *n*-hexane/iPrOH (4:1) at 0.5 mL/min. Peaks for (*S*)- and (*R*)-**4'** were detected at 11.3 min and 14.3 min, respectively, by monitoring the absorbance at 250 nm.

### Preparation of PGME amides **6a** and **6b**

In a manner similar to a procedure from [21], to a solution of **6** (1.0 mg, 3.2 μmol) in dry *N*,*N*-dimethylformamide (100 μL) and *N*,*N*-diisopropylethylamine (10 μL) were added (*R*)-PGME (1.9 mg, 9.6 μmol), PyBOP (3.6 mg, 7.0 μmol) and HOBt (1.0 mg, 7.4 μmol) at room temperature. After stirring for 3 h, ice-water was poured into the reaction mixture, which was then extracted with EtOAc. After evaporation of the solvent, the residue was purified on a silica gel thin-layer plate (Kieselgel 60F_254_; Merck Co.) developed by a mixture of CHCl_3_/MeOH (5:1). Extraction of the collected silica gel powder with MeOH gave (*R*)-PGME amide **6a** (0.5 mg).

(*R*)-PGME amide **6a**: ^1^H NMR (500 MHz, DMSO-*d*_6_) δ 10.61 (s, 1H, N*H*-1), 7.01 (s, 1H, H-2), 7.46 (d, *J* = 8.1 Hz, 1H, H-4), 6.78 (d, *J* = 7.9 Hz, 1H, H-5), 2.85 (dd, *J* = 14.3, 8.4 Hz, 1H, H-8a), 2.97 (dd, *J* = 14.0, 5.1 Hz, 1H, H-8b), 4.72 (m, 1H, H-9), 8.02 (d, *J* = 8.5 Hz, N*H*-11), 1.79 (s, 3H, H-13); HR–ESI–TOFMS (*m/z*): [M + Na]^+^ calcd for C_27_H_31_N_3_NaO_4_, 484.2207; found, 484.2209.

In the same manner as described for **6a**, **6b** (0.4 mg) was prepared from **1** and (*S*)-PGME.

(*S*)-PGME amide **6b**: ^1^H NMR (500 MHz, DMSO-*d*_6_) δ 10.64 (s, 1H, N*H*-1), 7.07 (d, *J* = 2.5 Hz, 1H, H-2), 7.53 (d, *J* = 8.0 Hz, 1H, H-4), 6.80 (d, *J* = 8.1 Hz, 1H, H-5), 2.88 (dd, *J* = 14.8, 9.5 Hz, 1H, H-8a), 3.06 (dd, *J* = 14.8, 4.8 Hz, 1H, H-8b), 4.68 (m, 1H, H-9), 8.01 (d, *J* = 8.3 Hz, 1H, N*H*-11), 1.73 (s, 3H, H-13); HR–ESI–TOFMS (*m/z*): [M + Na]^+^ calcd for C_27_H_31_N_3_NaO_4_, 484.2207; found, 484.2207.

### Cytotoxicity assay

The cytotoxicity assay was carried out against P388 murine leukemia cells in the same manner as reported previously [36]. The IC_50_ of a reference drug doxorubicin hydrochloride was 0.13 μM.

### Antimicrobial assay

Antimicrobial assays were carried out against five bacteria, *K. rhizophila* ATCC 9341, *S. aureus* FDA209P JC-1, *E. coli* NIHJ JC-2, *R. solanacearum* SUPP1541, and *R. radiobacter* NBRC 14554, and a yeast *C. albicans* NBRC 0197 in the same manner as reported previously [36]. The MIC of the reference antibiotic kanamycin was 0.31 μg/mL (against *K. rhizophila*).

## Supporting Information

File 1 Copies of NMR spectra.
