# Supplementary material for: Unsaturated fatty acids and a prenylated tryptophan derivative from a rare actinomycete of the genus Couchioplanes
Source: Beilstein J Org Chem. 2021 Dec 16;17:2939–49. doi: 10.3762/bjoc.17.203 (PMC8685556; doi:10.3762/bjoc.17.203)

BEILSTEIN JOURNAL OF ORGANIC CHEMISTRY

Supporting Information

for

Unsaturated fatty acids and a prenylated tryptophan derivative from a rare actinomycete of the genus *Couchioplanes*

Shun Saito, Kanji Indo, Naoya Oku, Hisayuki Komaki, Masashi Kawasaki and Yasuhiro Igarashi

Beilstein J. Org. Chem. **2021,** *17,* 2939–2949. doi:10.3762/bjoc.17.203

Copies of NMR spectra

License and Terms: This is a supporting information file under the terms of the Creative Commons Attribution License (https://creativecommons.org/licenses/by/4.0). Please note that the reuse, redistribution and reproduction in particular requires that the author(s) and source are credited and that individual graphics may be subject to special legal provisions.
The license is subject to the *Beilstein Journal of Organic Chemistry* terms and conditions: (https://www.beilstein-journals.org/bjoc/terms)

Table of contents

Figure S1. ^{1}H NMR spectrum of compound **1** (500 MHz, $CDCl_3$) S4
Figure S2. ^{13}C NMR spectrum of **1** (125 MHz, $CDCl_3$) S5
Figure S3. ^{1}H-^{1}H COSY spectrum of **1** (500 MHz, $CDCl_3$) S6
Figure S4. HSQC spectrum of **1** (500 MHz, $CDCl_3$) S7
Figure S5. HMBC spectrum of **1** (500 MHz, $CDCl_3$) S8
Figure S6. NOESY spectrum of **1** (500 MHz, $CDCl_3$) S9
Figure S7. ^{1}H NMR spectrum of compound **2** (500 MHz, $CDCl_3$) S10
Figure S8. ^{13}C NMR spectrum of **2** (125 MHz, $CDCl_3$) S11
Figure S9. ^{1}H-^{1}H COSY spectrum of **2** (500 MHz, $CDCl_3$) S12
Figure S10. HSQC spectrum of **2** (500 MHz, $CDCl_3$) S13
Figure S11. HMBC spectrum of **2** (500 MHz, $CDCl_3$) S14
Figure S12. NOESY spectrum of **2** (500 MHz, $CDCl_3$) S15
Figure S13. ^{1}H NMR spectrum of compound **3** (500 MHz, $CDCl_3$) S16
Figure S14. ^{13}C NMR spectrum of **3** (125 MHz, $CDCl_3$) S17
Figure S15. ^{1}H-^{1}H COSY spectrum of **3** (500 MHz, $CDCl_3$) S18
Figure S16. HSQC spectrum of **3** (500 MHz, $CDCl_3$) S19
Figure S17. HMBC spectrum of **3** (500 MHz, $CDCl_3$) S20
Figure S18. NOESY spectrum of **3** (500 MHz, $CDCl_3$) S21
Figure S19. ^{1}H NMR spectrum of compound **4** (500 MHz, $CDCl_3$) S22
Figure S20. ^{13}C NMR spectrum of **4** (125 MHz, $CDCl_3$) S23
Figure S21. ^{1}H-^{1}H COSY spectrum of **4** (500 MHz, $CDCl_3$) S24
Figure S22. HSQC spectrum of **4** (500 MHz, $CDCl_3$) S25
Figure S23. HMBC spectrum of **4** (500 MHz, $CDCl_3$) S26
Figure S24. NOESY spectrum of **4** (500 MHz, $CDCl_3$) S27
Figure S25. ^{1}H-^{1}H COSY spectrum of **4’a** (500 MHz, $CDCl_3$) S28
Figure S26. ^{1}H-^{1}H COSY spectrum of **4’b** (500 MHz, $CDCl_3$) S29
Figure S27. ^{1}H NMR spectrum of compound **5** (500 MHz, $CDCl_3$) S30
Figure S28. ^{13}C NMR spectrum of **5** (125 MHz, $CDCl_3$) S31
Figure S29. ^{1}H-^{1}H COSY spectrum of **5** (500 MHz, $CDCl_3$) S32
Figure S30. HSQC spectrum of **5** (500 MHz, $CDCl_3$) S33
Figure S31. HMBC spectrum of **5** (500 MHz, $CDCl_3$) S34
Figure S32. NOESY spectrum of **5** (500 MHz, $CDCl_3$) S35
Figure S33. ^{1}H NMR spectrum of compound **6** (500 MHz, $CDCl_3$) S36
Figure S34. ^{13}C NMR spectrum of **6** (125 MHz, $CDCl_3$) S37

Figure S35. ^{1}H-^{1}H COSY spectrum of **6** (500 MHz, $CDCl_3$) S38
Figure S36. HSQC spectrum of **6** (500 MHz, $CDCl_3$) S39
Figure S37. HMBC spectrum of **6** (500 MHz, $CDCl_3$) S40
Figure S38. ^{1}H NMR spectrum of **6a** (500 MHz, DMSO-d_6) S41
Figure S39. ^{1}H-^{1}H COSY spectrum of **6a** (500 MHz, DMSO-d_6) S42
Figure S40. HSQC spectrum of **6a** (500 MHz, DMSO-d_6) S43
Figure S41. HMBC spectrum of **6a** (500 MHz, DMSO-d_6) S44
Figure S42. ^{1}H NMR spectrum of **6b** (500 MHz, DMSO-d_6) S45
Figure S43. ^{1}H-^{1}H COSY spectrum of **6b** (500 MHz, DMSO-d_6) S46
Figure S44. HSQC spectrum of **6b** (500 MHz, DMSO-d_6) S47
Figure S45. HMBC spectrum of **6b** (500 MHz, DMSO-d_6) S48

Figure S1. ^{1}H NMR spectrum of compound **1** (500 MHz, $CDCl_3$).

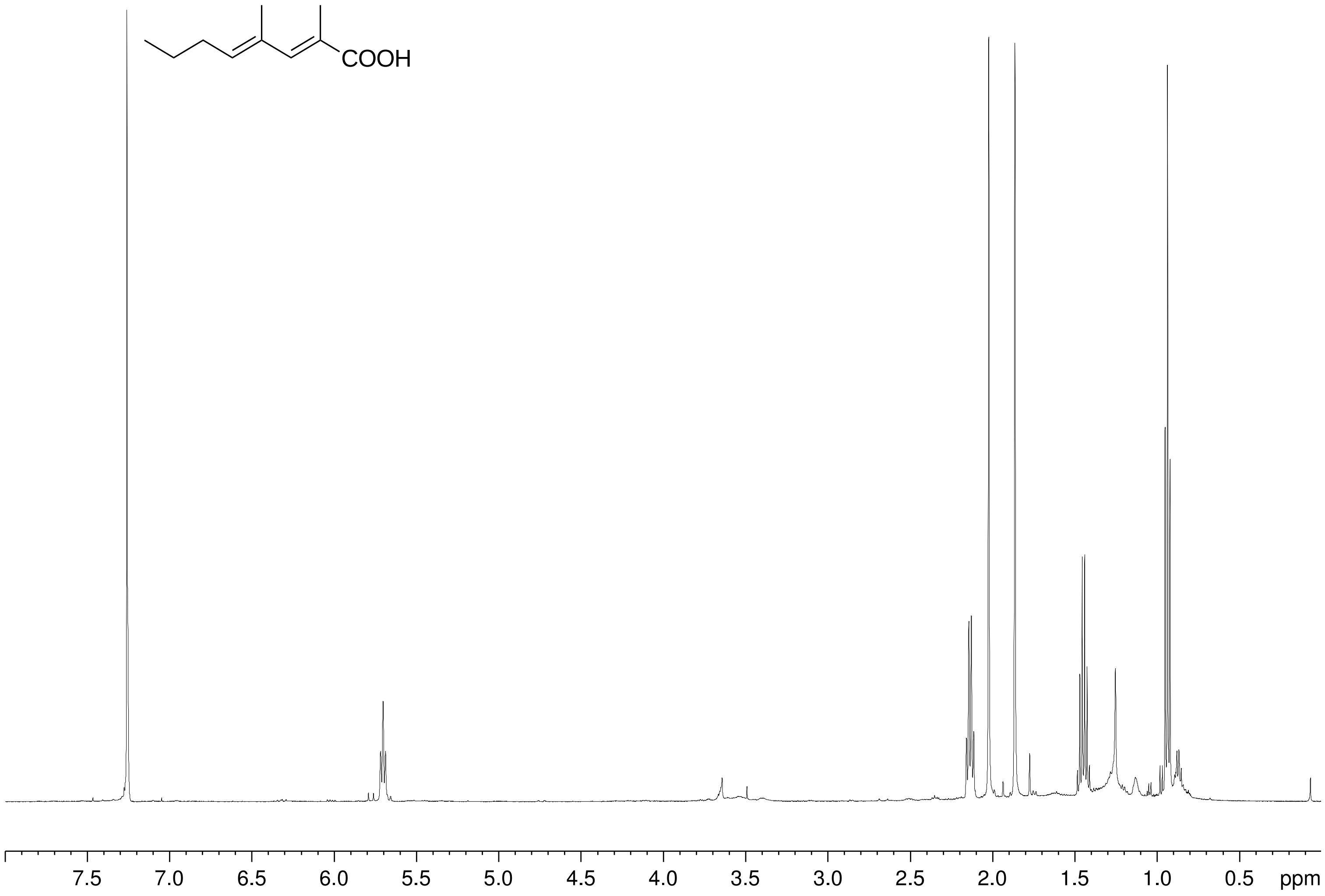

Figure S2. ${}^{13}C$ NMR spectrum of **1** (500 MHz, $CDCl_3$).

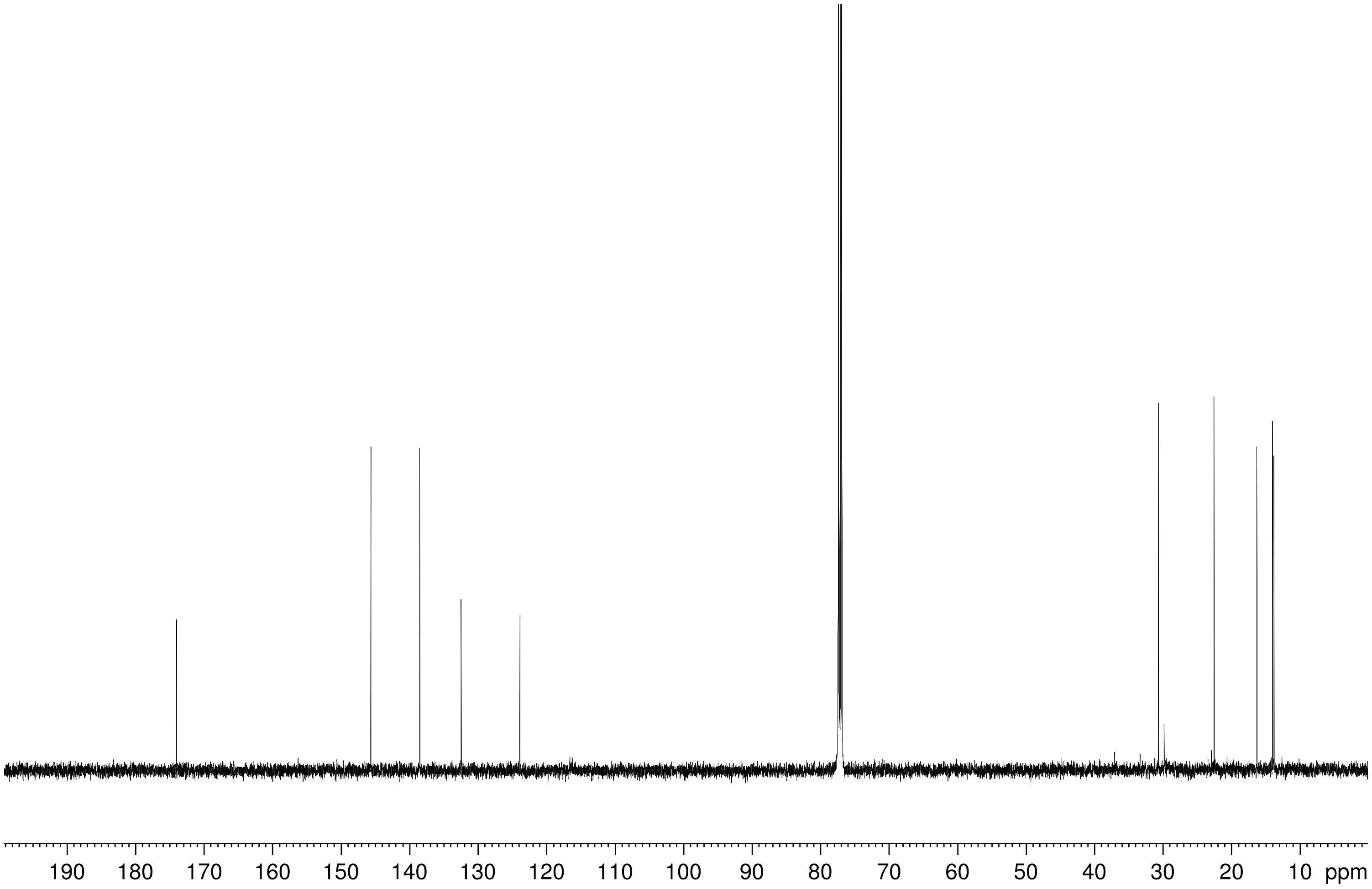

Figure S3. ^{1}H-^{1}H COSY spectrum of **1** (500 MHz, $CDCl_3$).

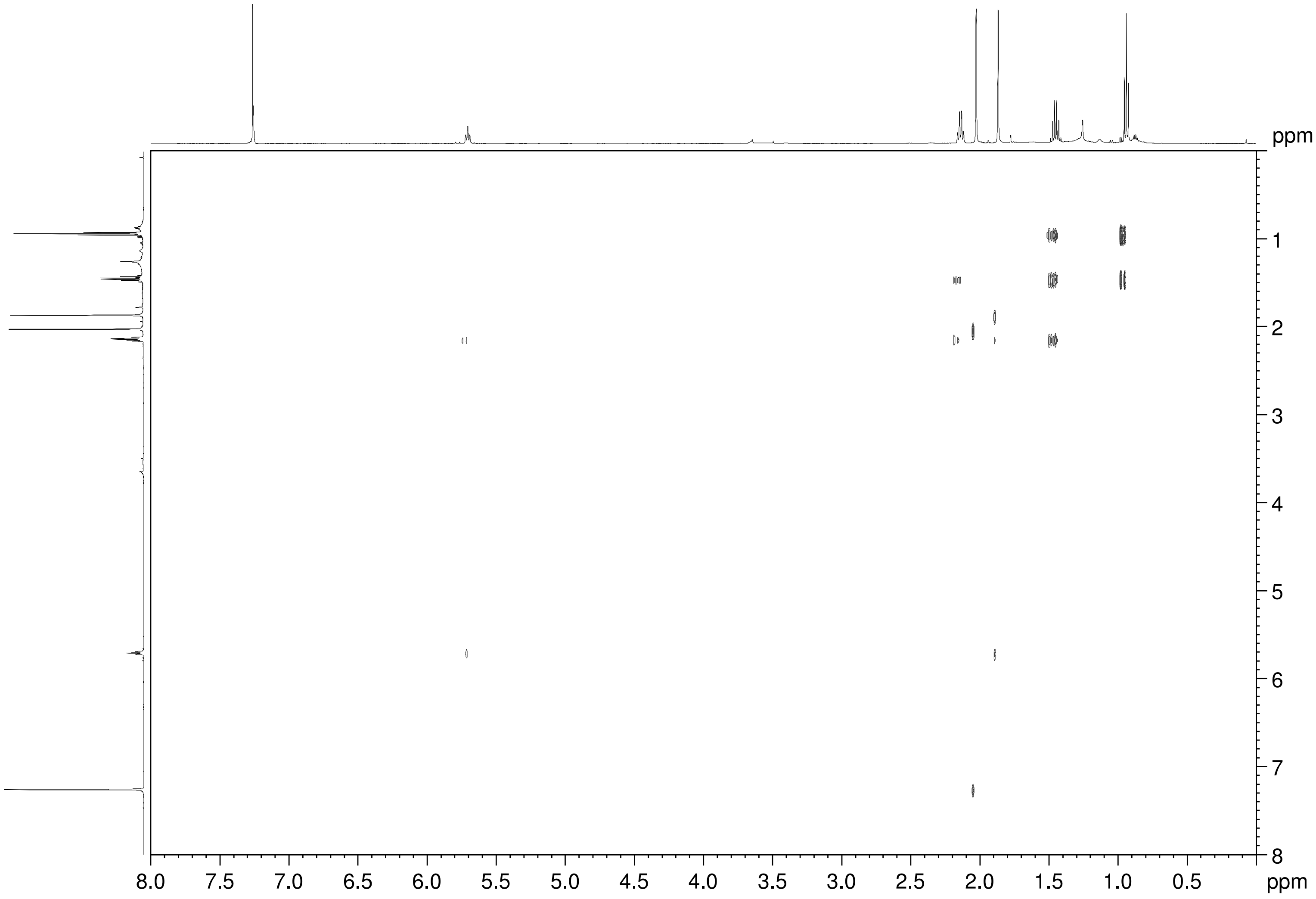

Figure S4. HSQC spectrum of **1** (500 MHz, $CDCl_3$).

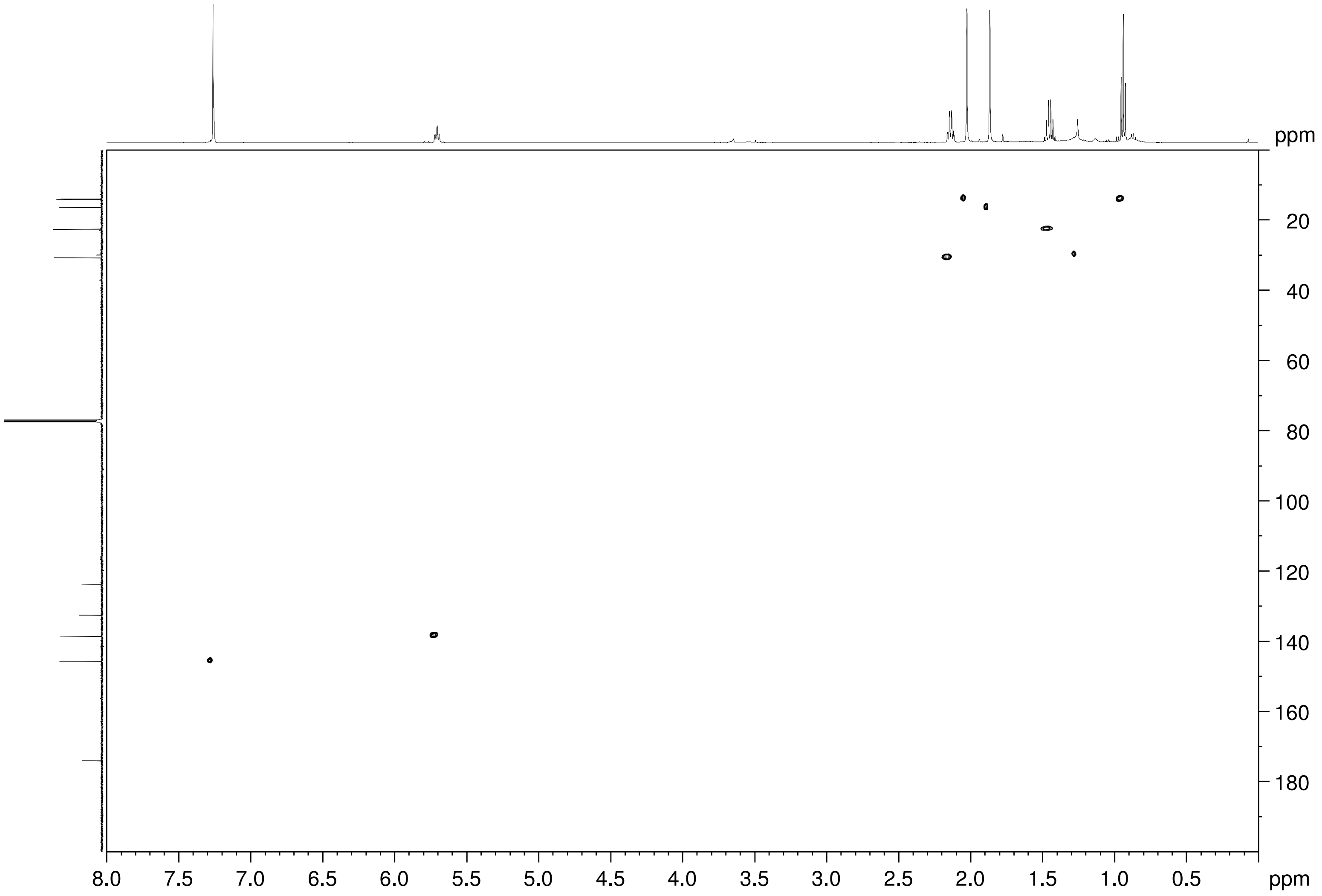

Figure S5. HMBC spectrum of **1** (500 MHz, $CDCl_3$).

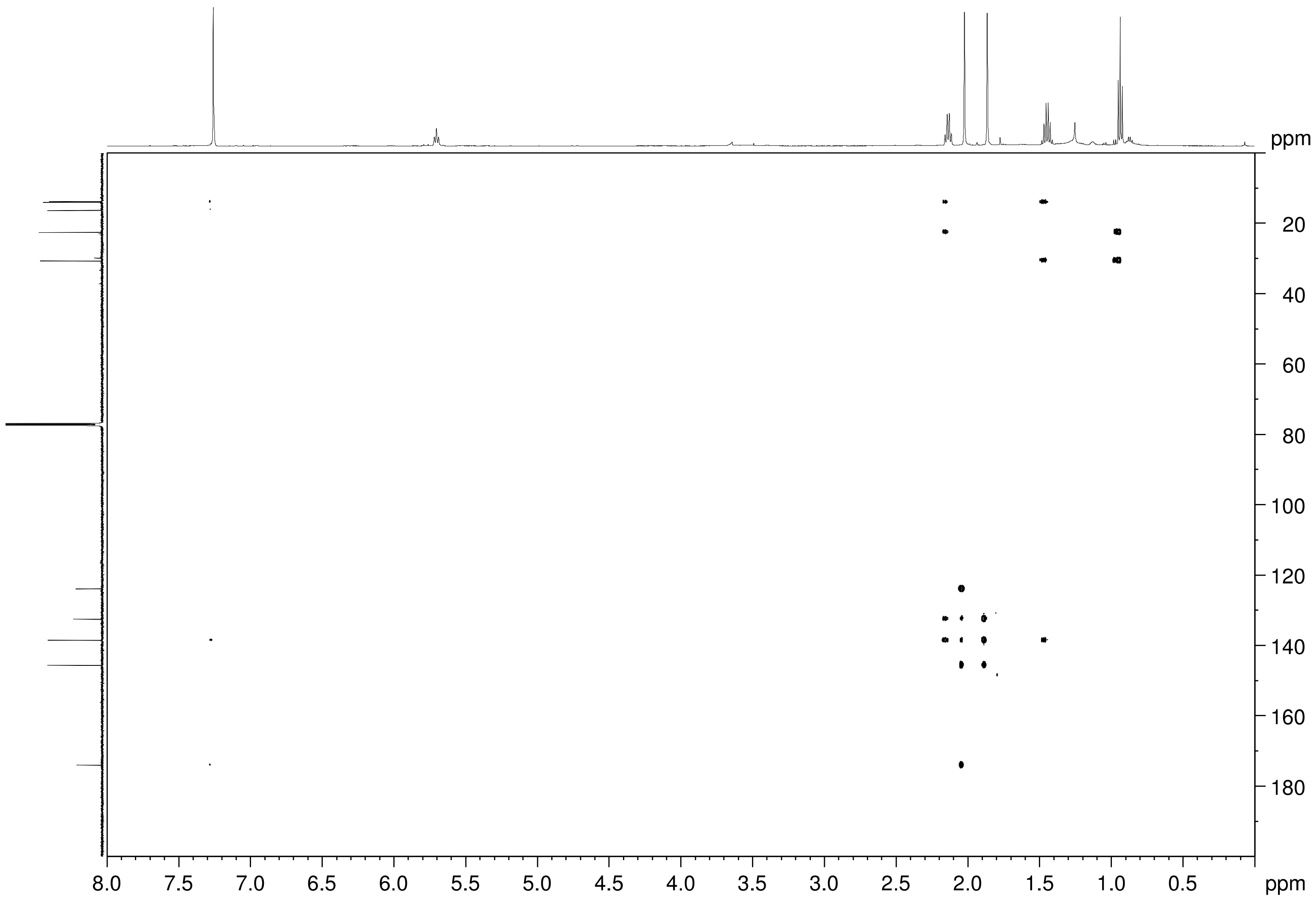

Figure S6. NOESY spectrum of **1** (500 MHz, $CDCl_3$).

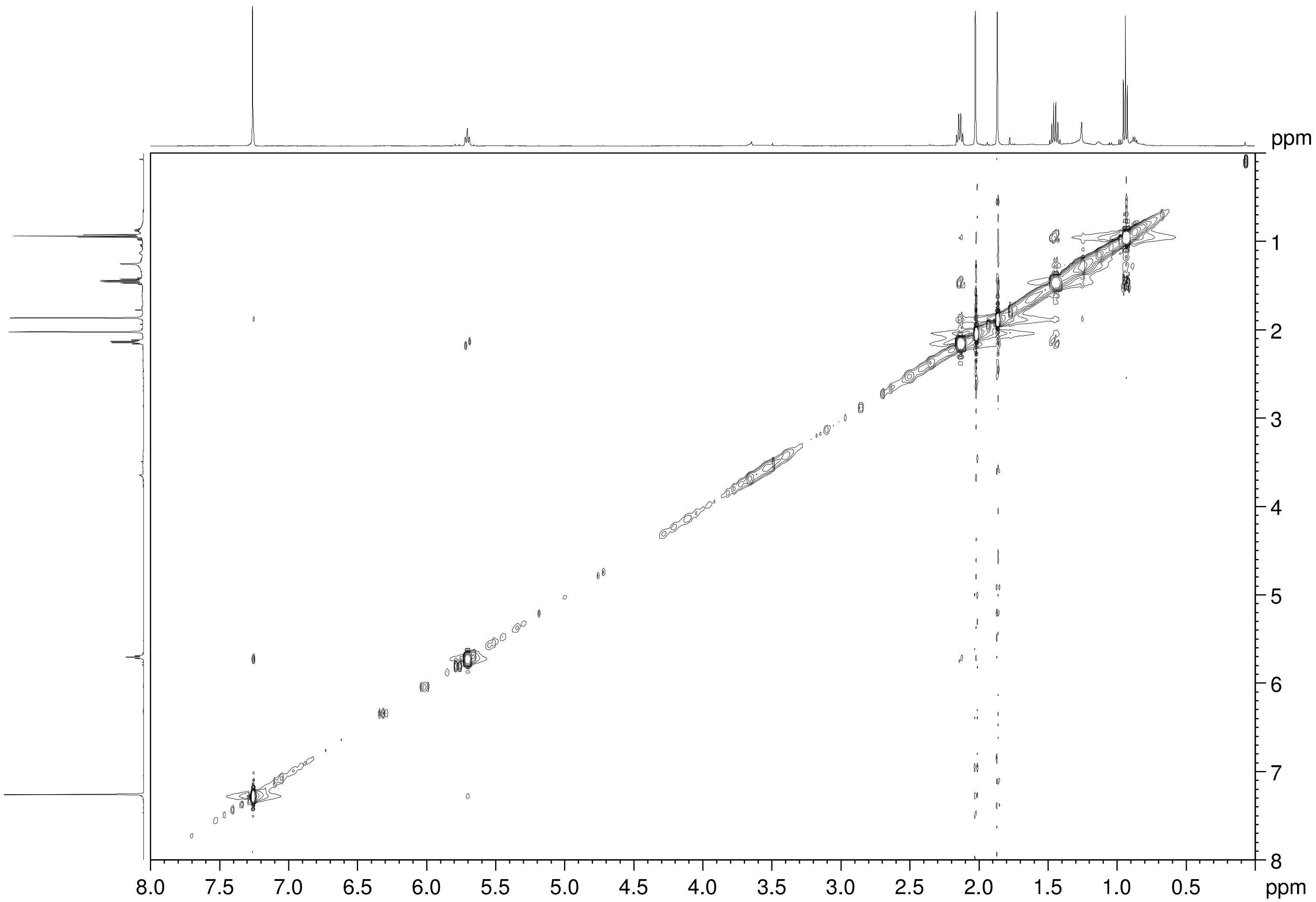

Figure S7. ^{1}H NMR spectrum of compound **2** (500 MHz, $CDCl_3$).

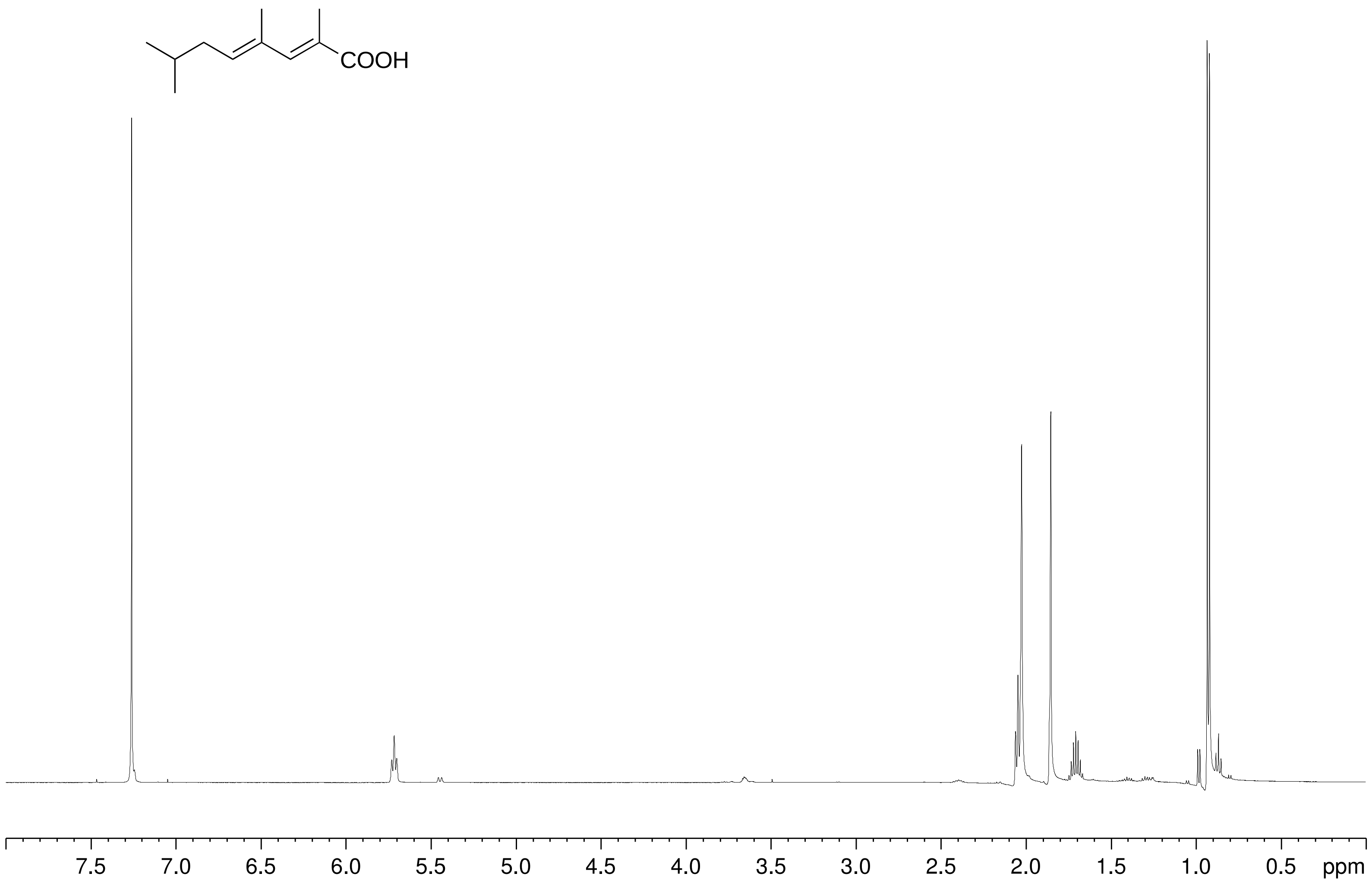

Figure S8. ^{13}C NMR spectrum of **2** (125 MHz, $CDCl_3$).

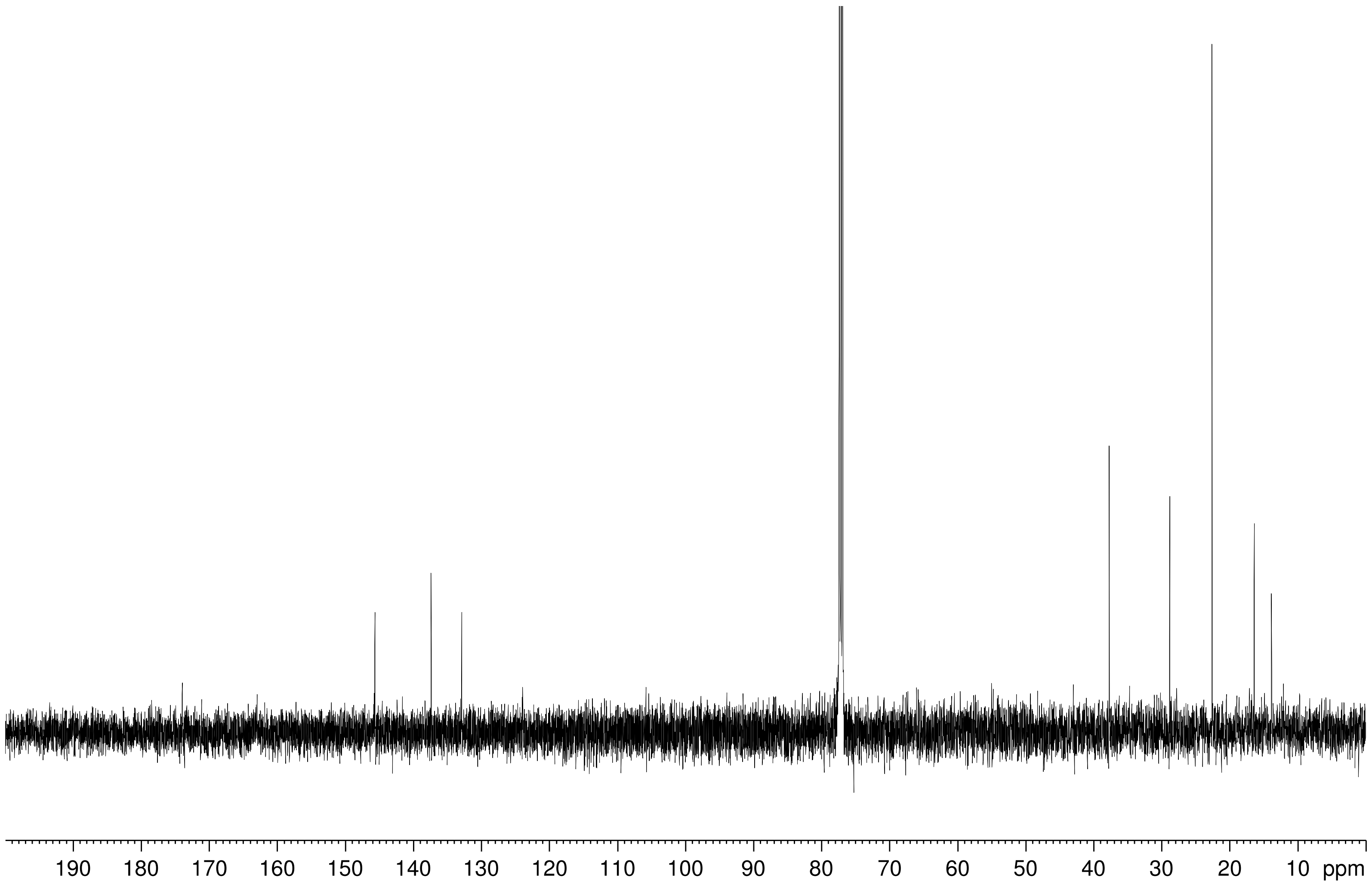

Figure S9. ^1H-^1H COSY spectrum of **2** (500 MHz, $CDCl_3$).

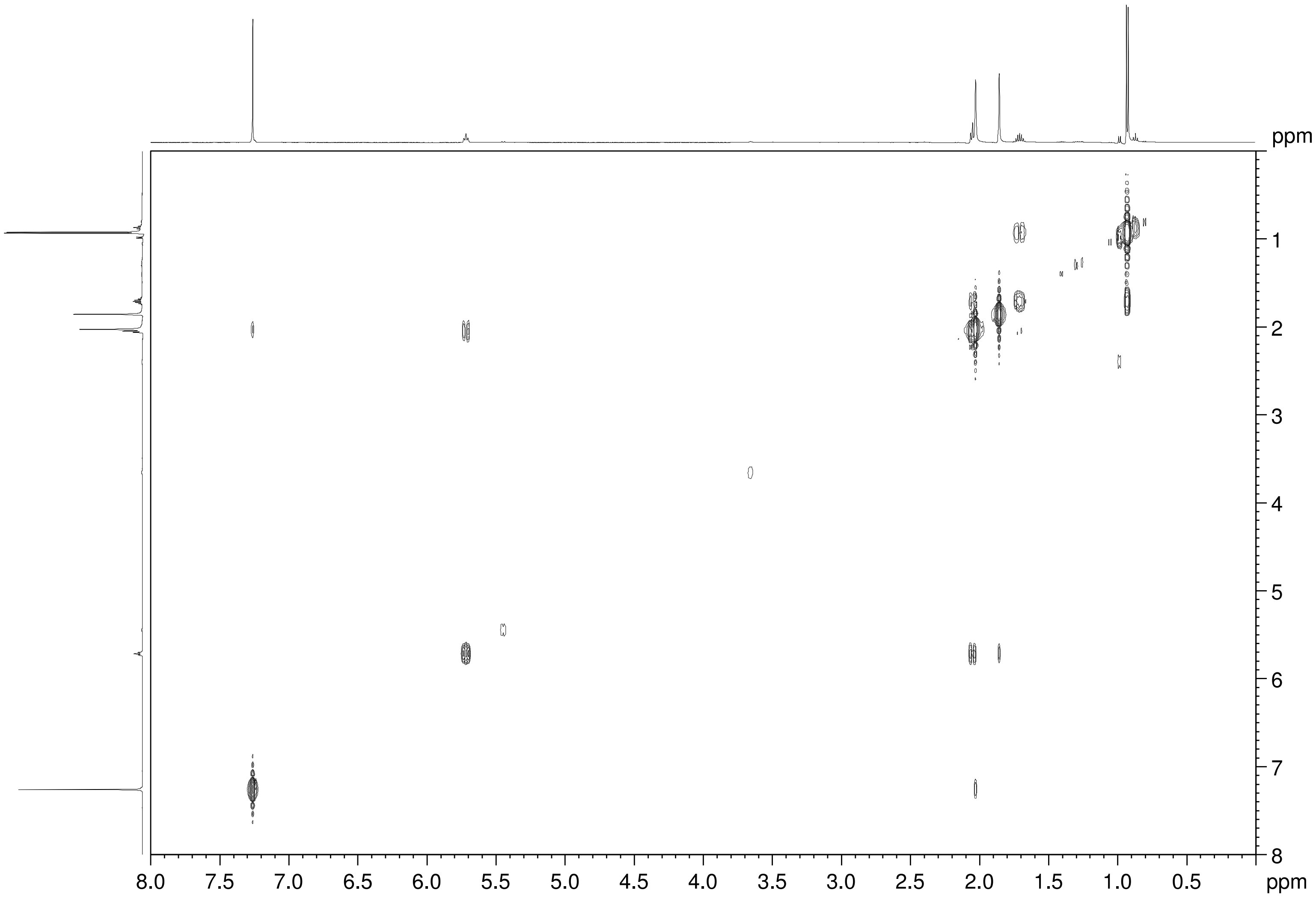

Figure S10. HSQC spectrum of **2** (500 MHz, $CDCl_3$)**.**

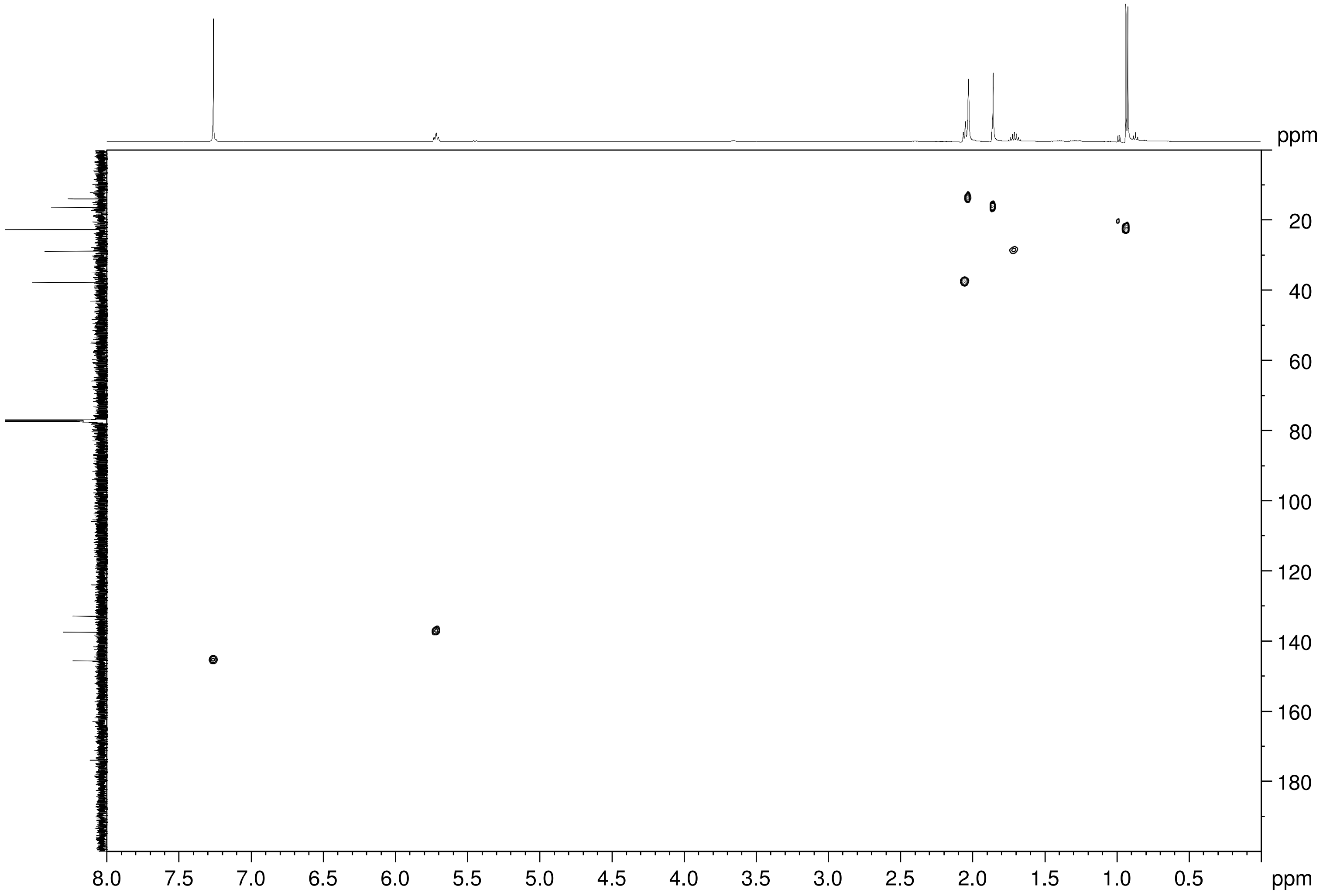

Figure S11. HMBC spectrum of **2** (500 MHz, $CDCl_3$).

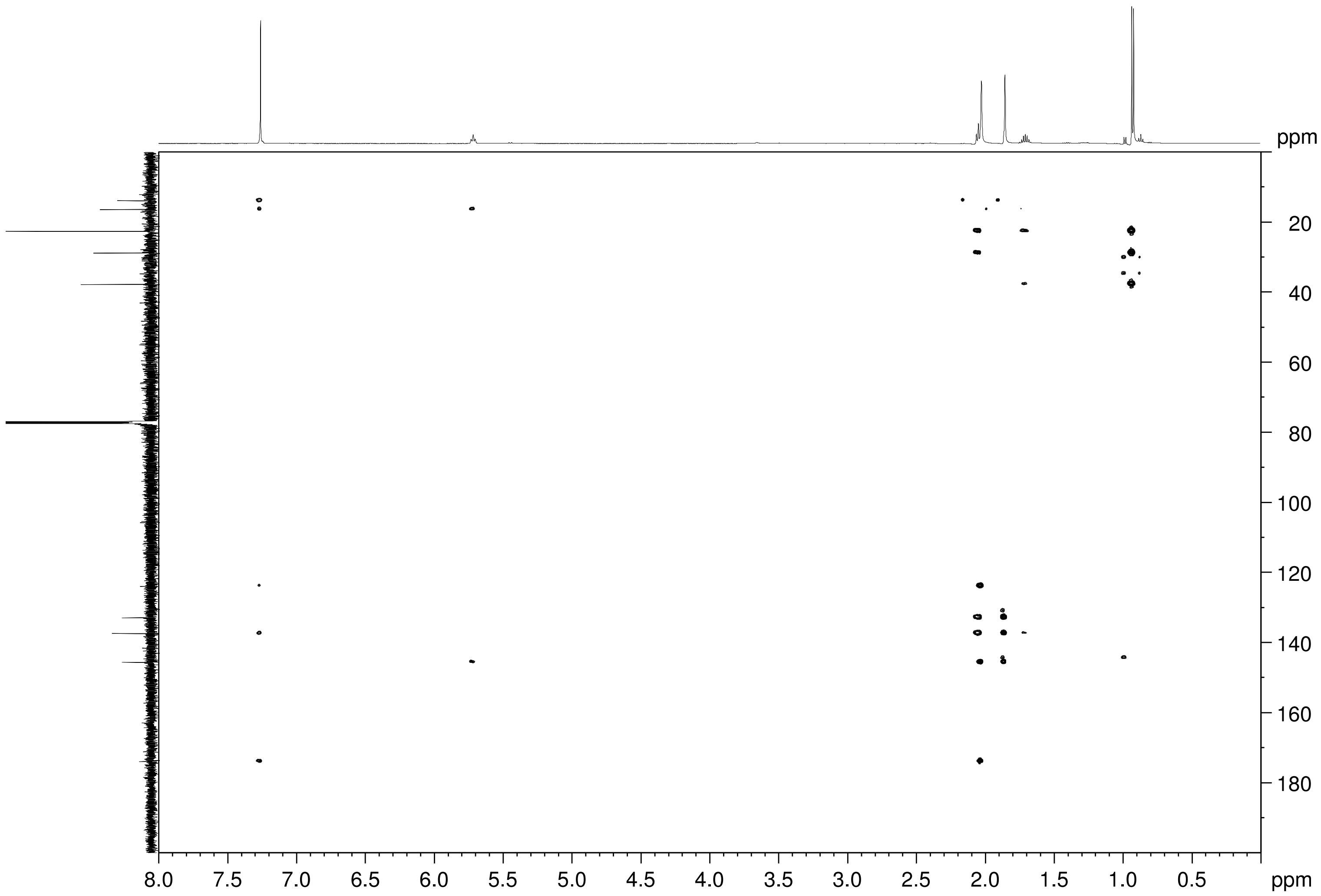

Figure S12. NOESY spectrum of **2** (500 MHz, $CDCl_3$).

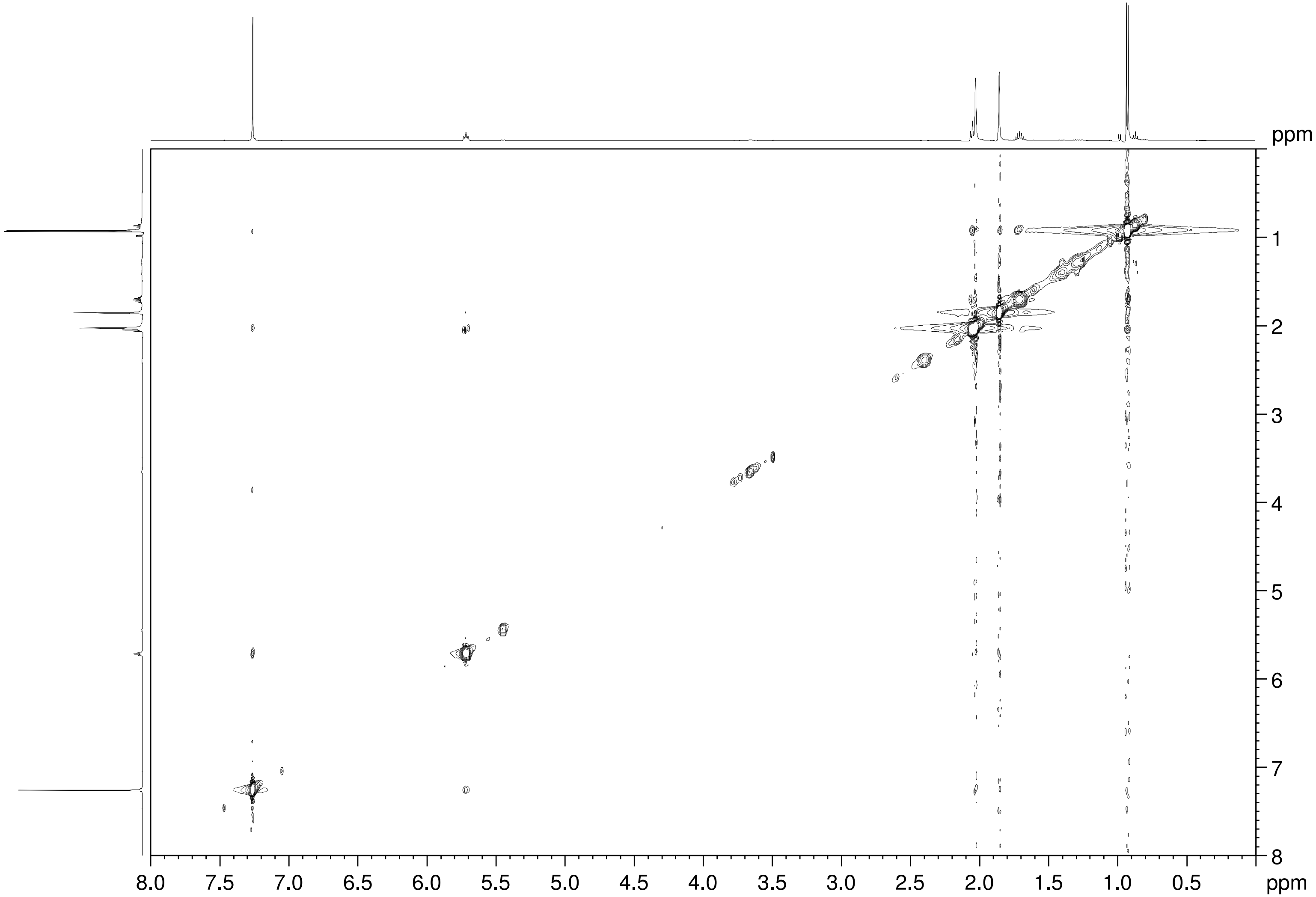

Figure S13. ^{1}H NMR spectrum of compound **3** (500 MHz, $CDCl_3$).

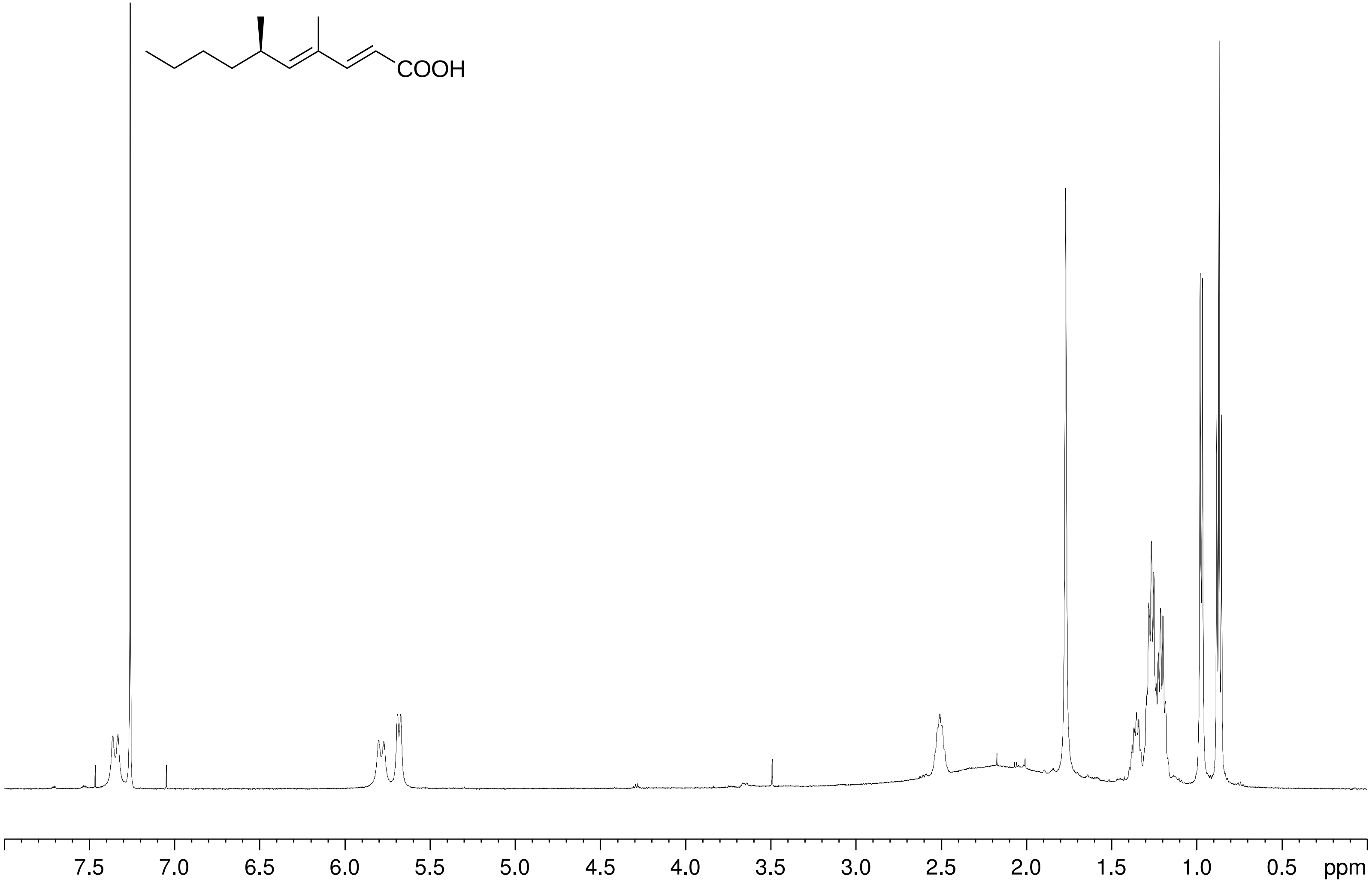

Figure S14. ^{13}C NMR spectrum of **3** (125 MHz, $CDCl_3$).

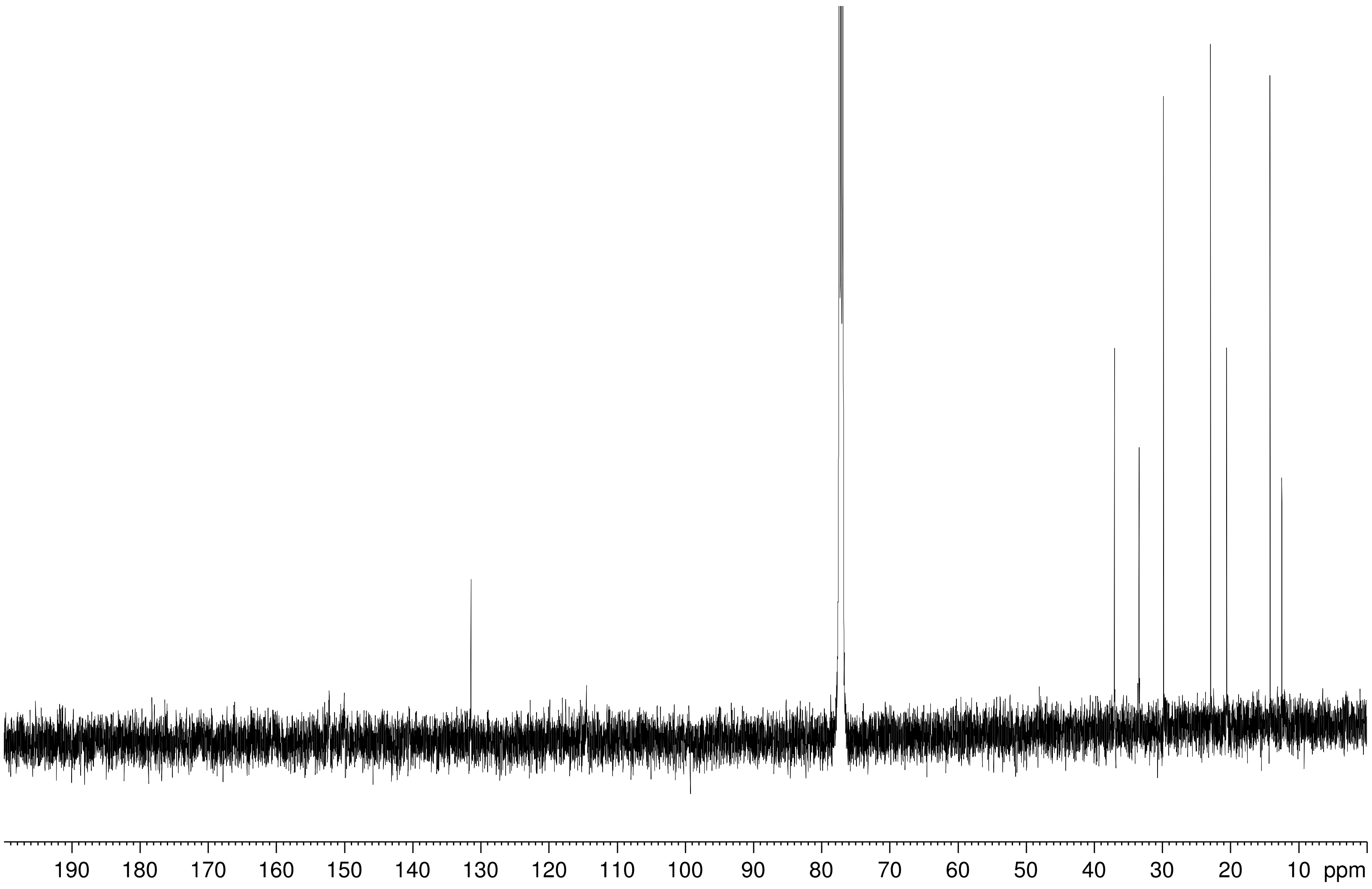

Figure S15. ^{1}H-^{1}H COSY spectrum of **3** (500 MHz, $CDCl_3$).

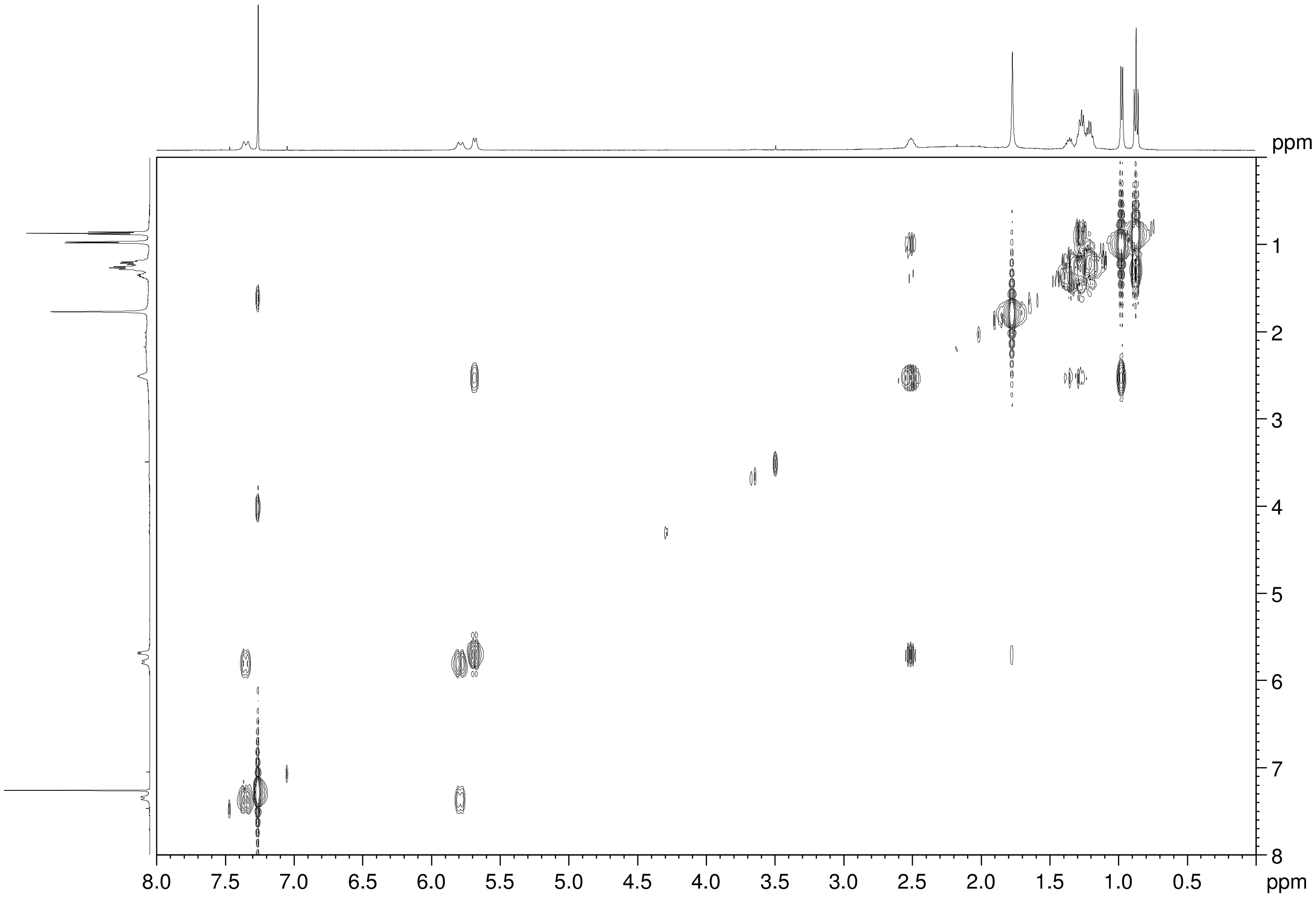

Figure S16. HSQC spectrum of **3** (500 MHz, $CDCl_3$).

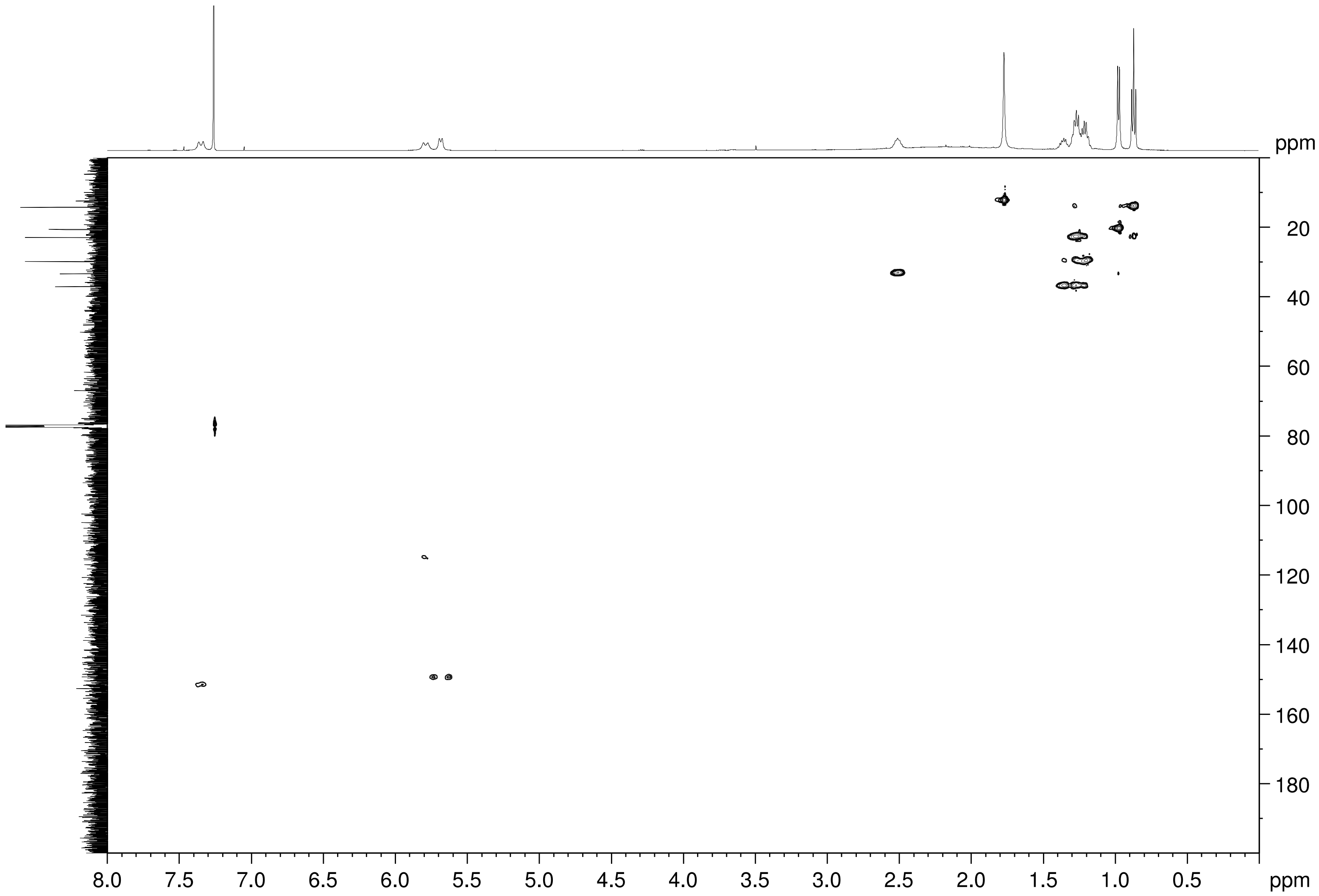

Figure S17. HMBC spectrum of **3** (500 MHz, $CDCl_3$).

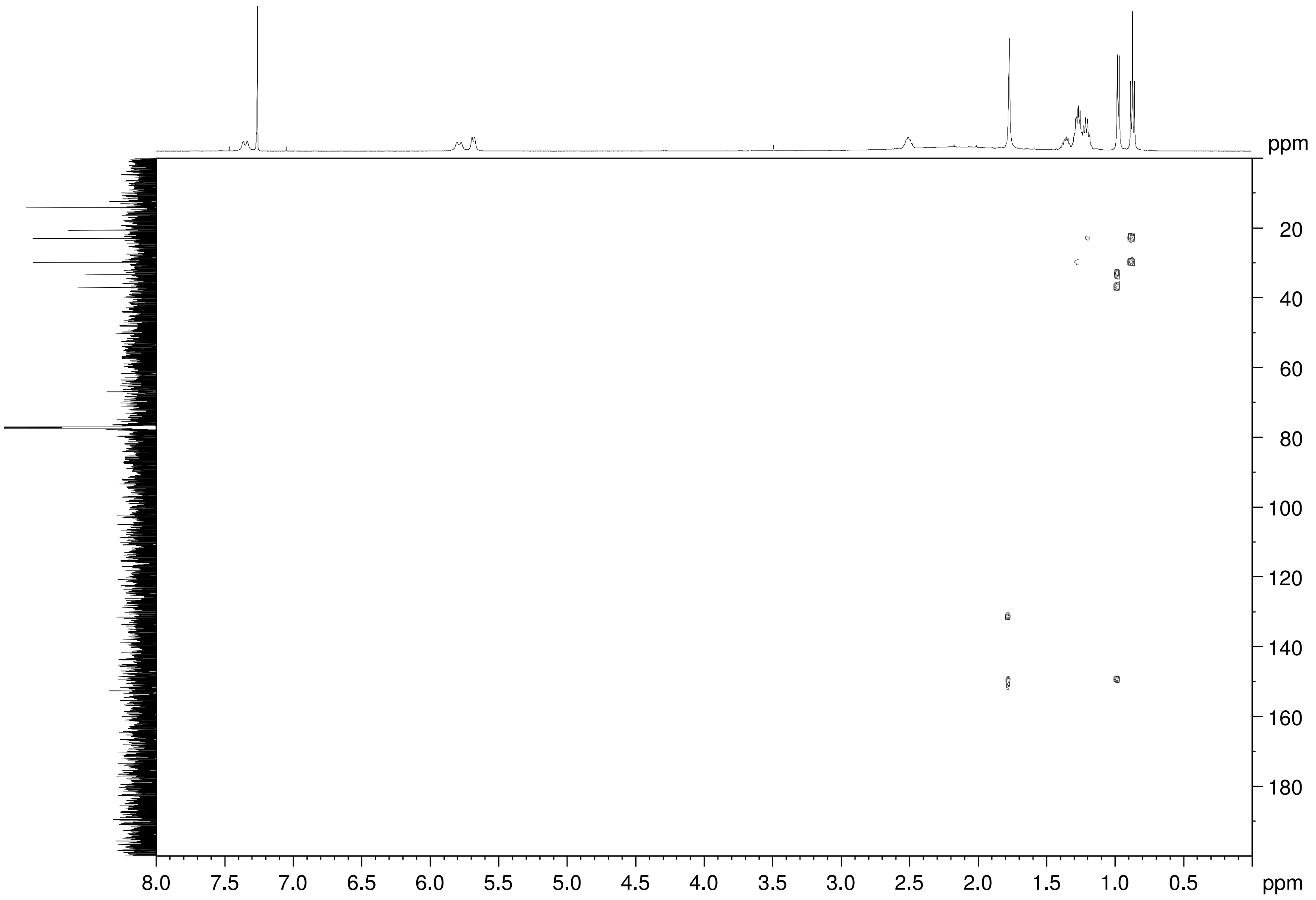

Figure S18. NOESY spectrum of **3** (500 MHz, $CDCl_3$).

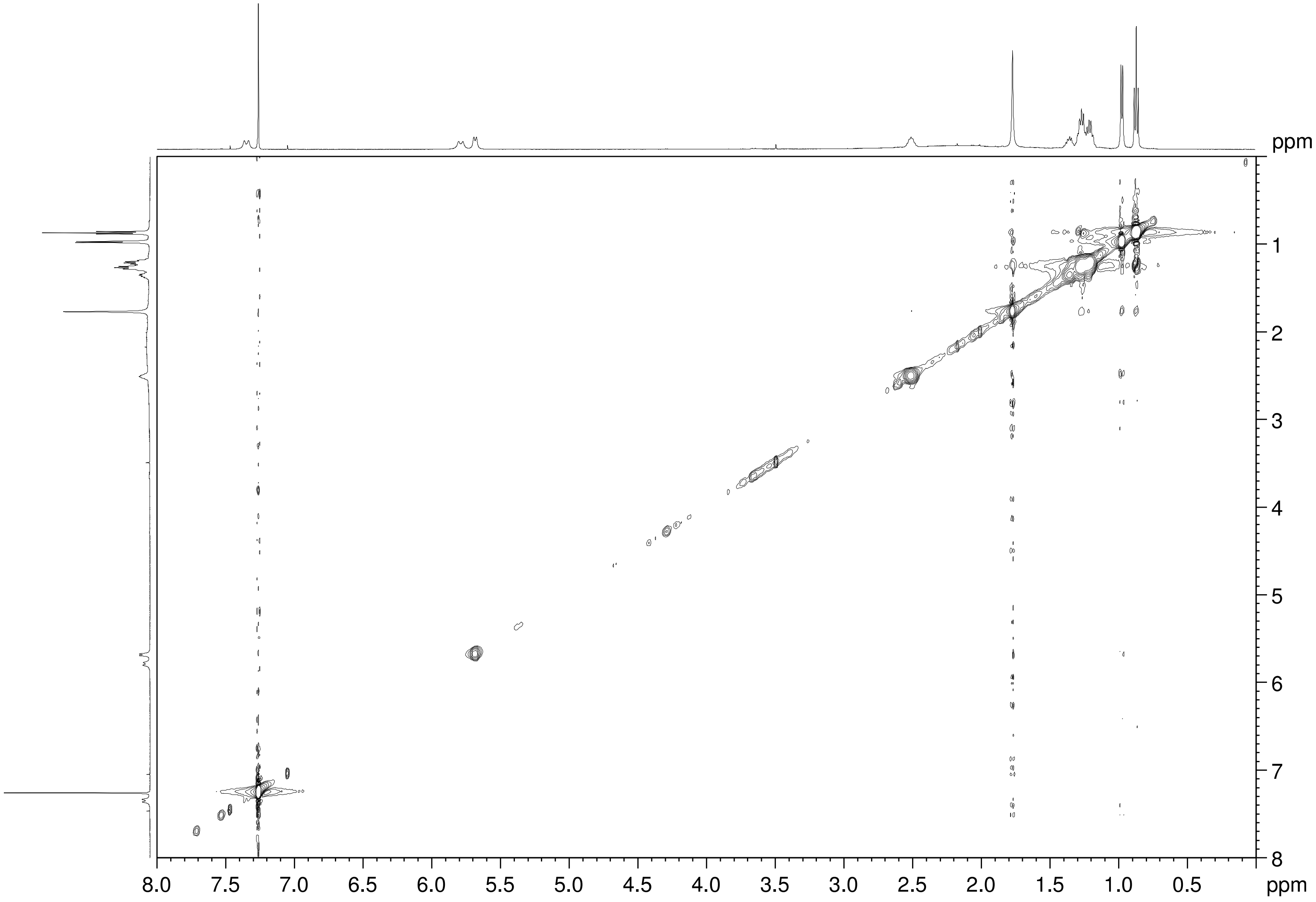

Figure S19. ^{1}H NMR spectrum of compound **4** (500 MHz, $CDCl_3$).

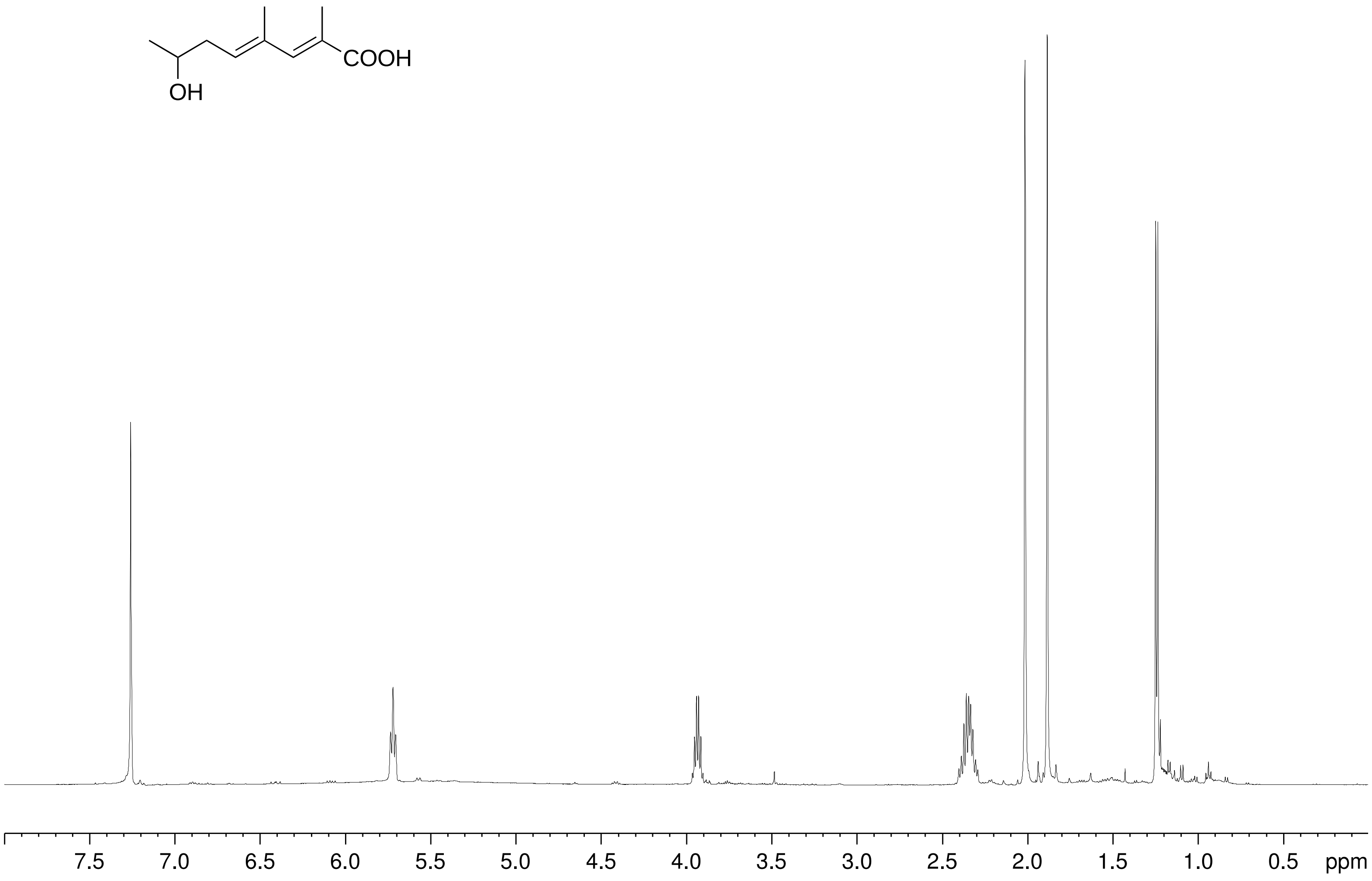

Figure S20. ^{13}C NMR spectrum of **4** (125 MHz, $CDCl_3$).

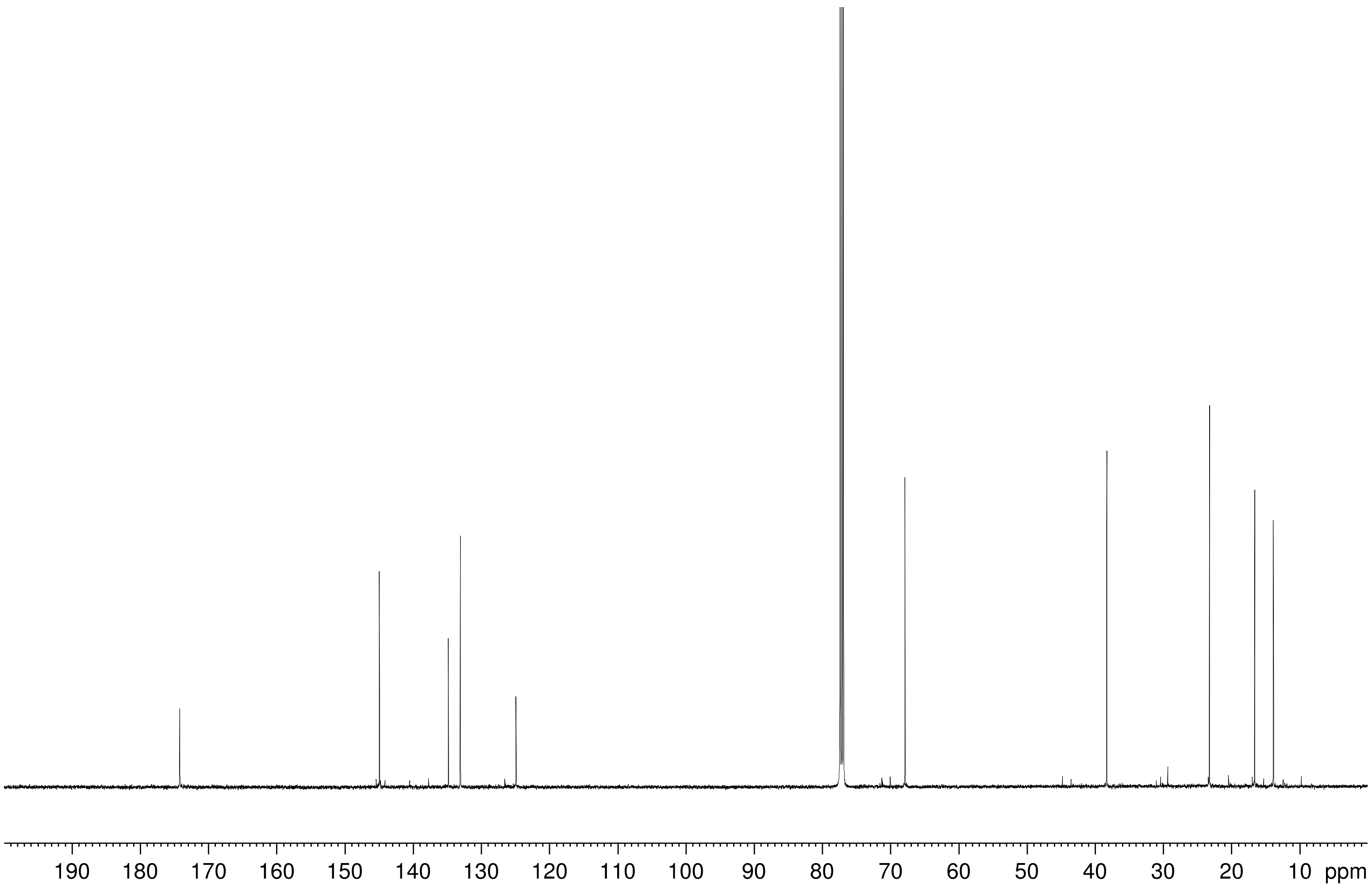

Figure S21. ^{1}H-^{1}H COSY spectrum of **4** (500 MHz, $CDCl_3$).

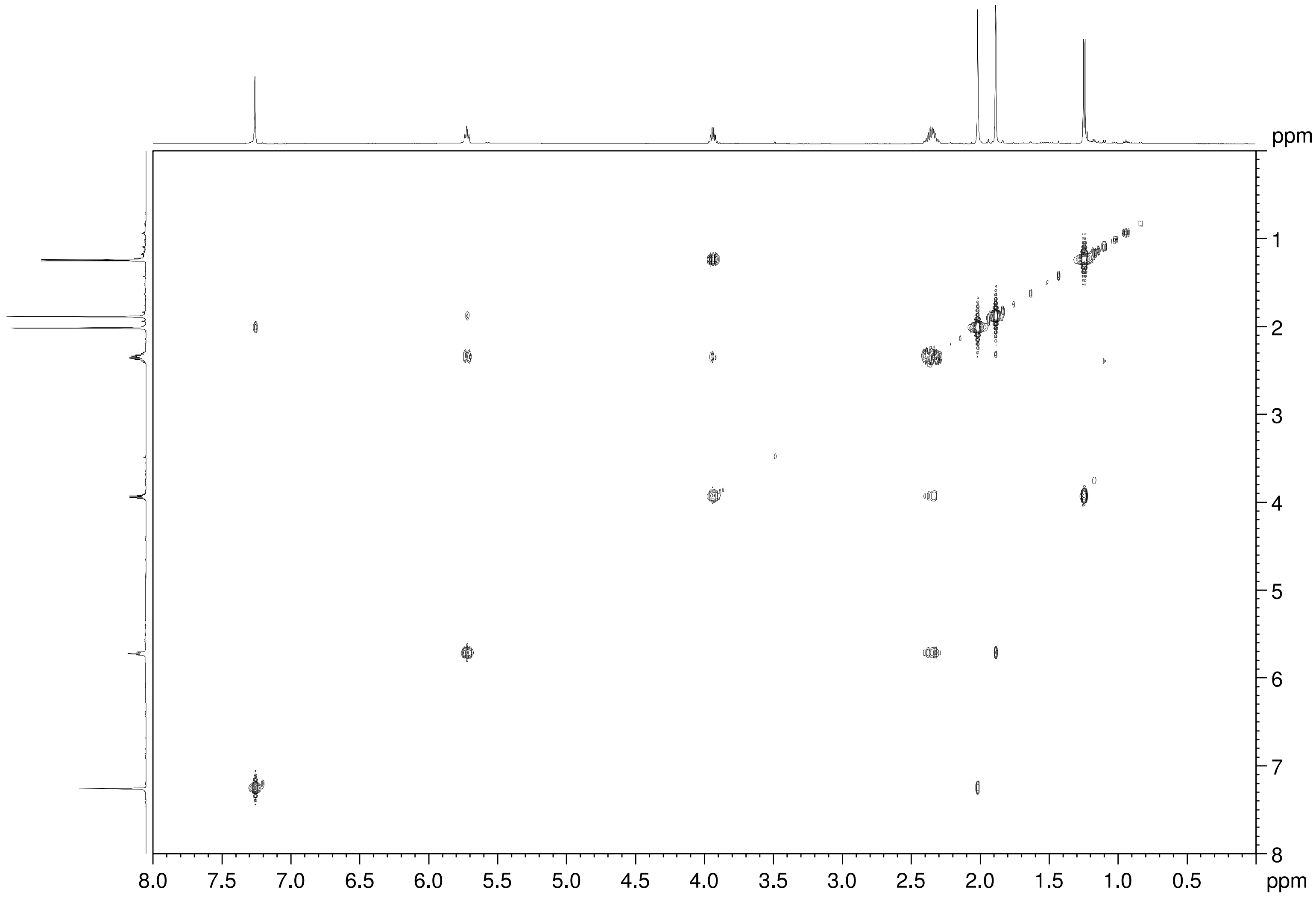

Figure S22. HSQC spectrum of **4** (500 MHz, $CDCl_3$).

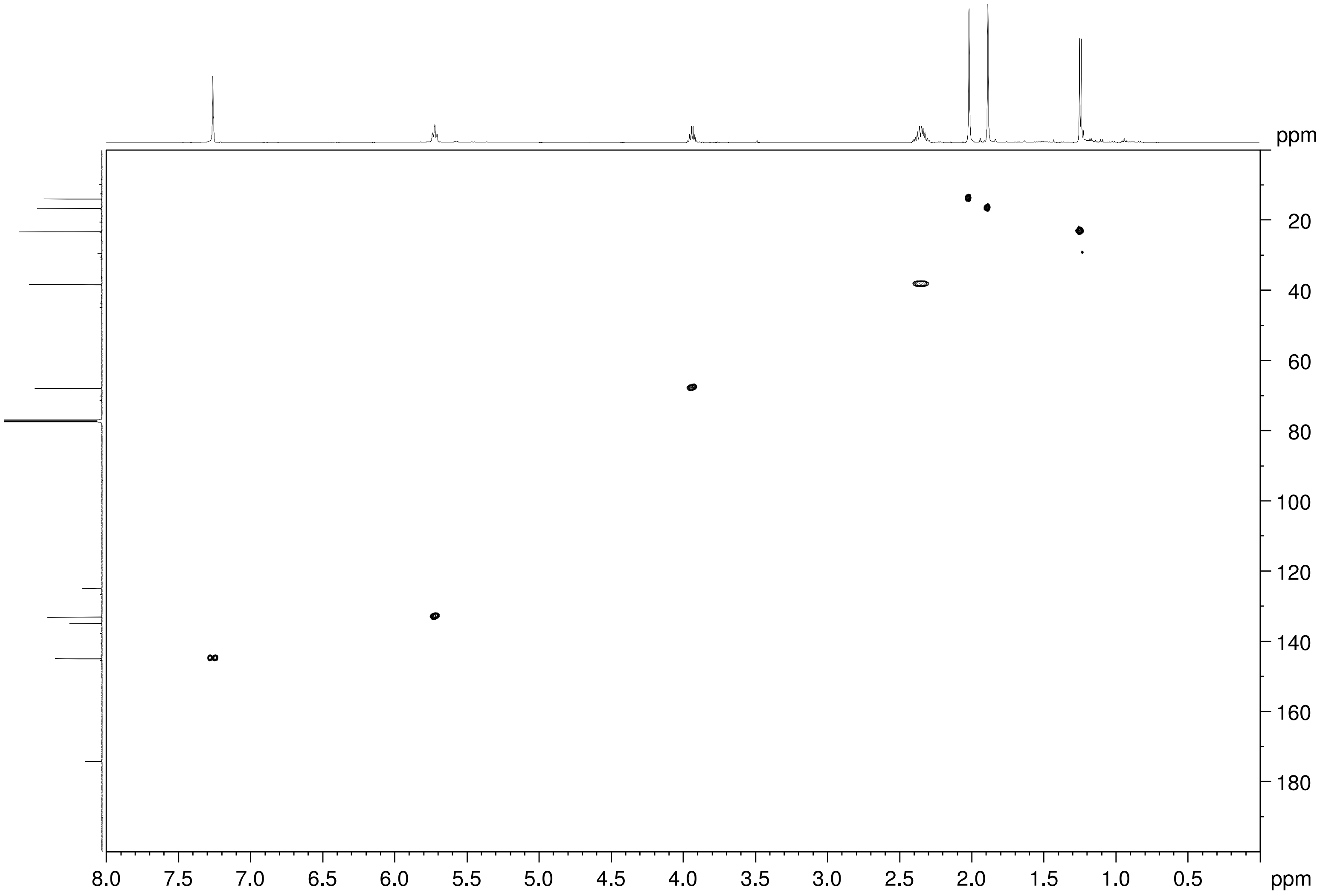

Figure S23. HMBC spectrum of **4** (500 MHz, $CDCl_3$).

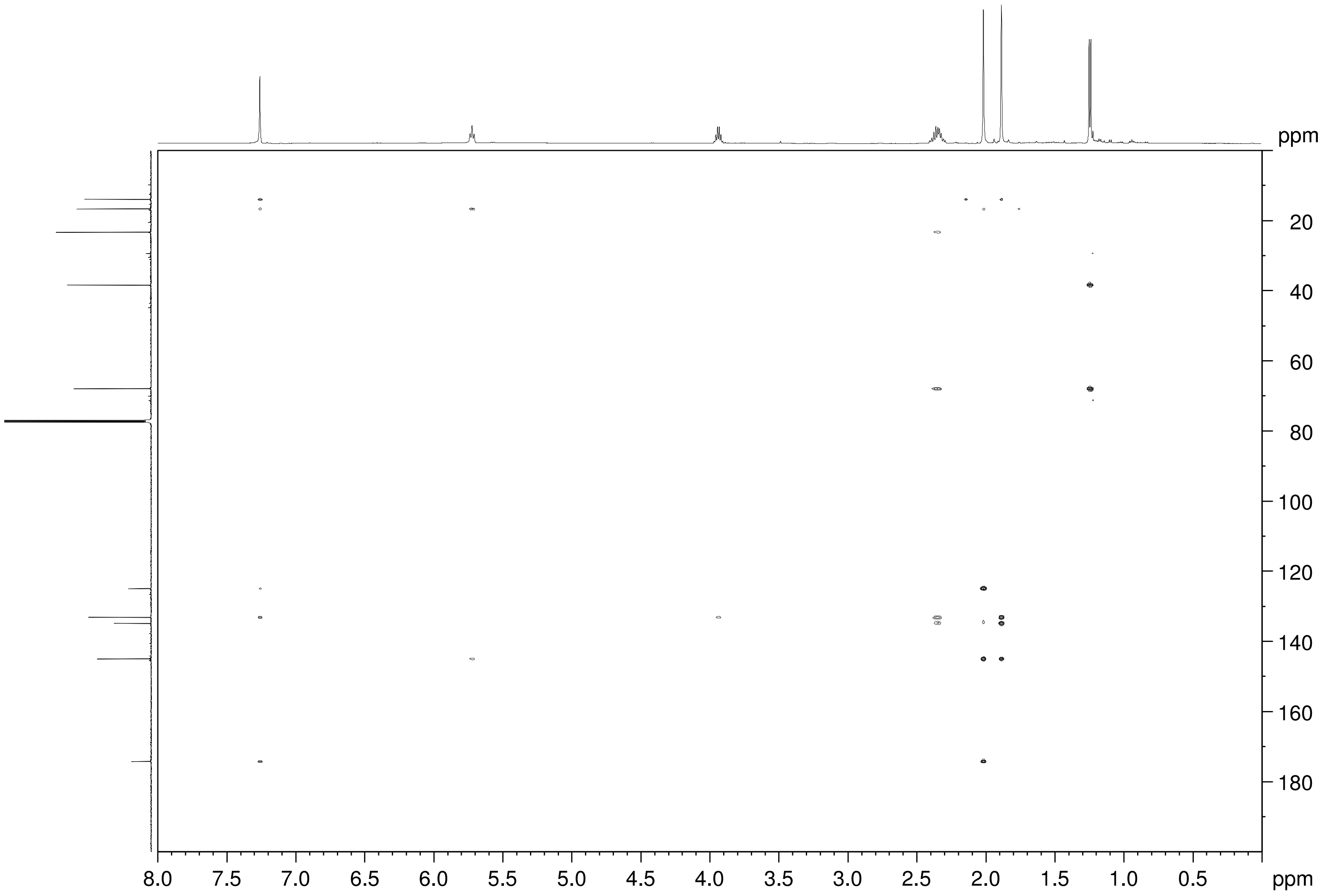

Figure S24. NOESY spectrum of **4** (500 MHz, $CDCl_3$).

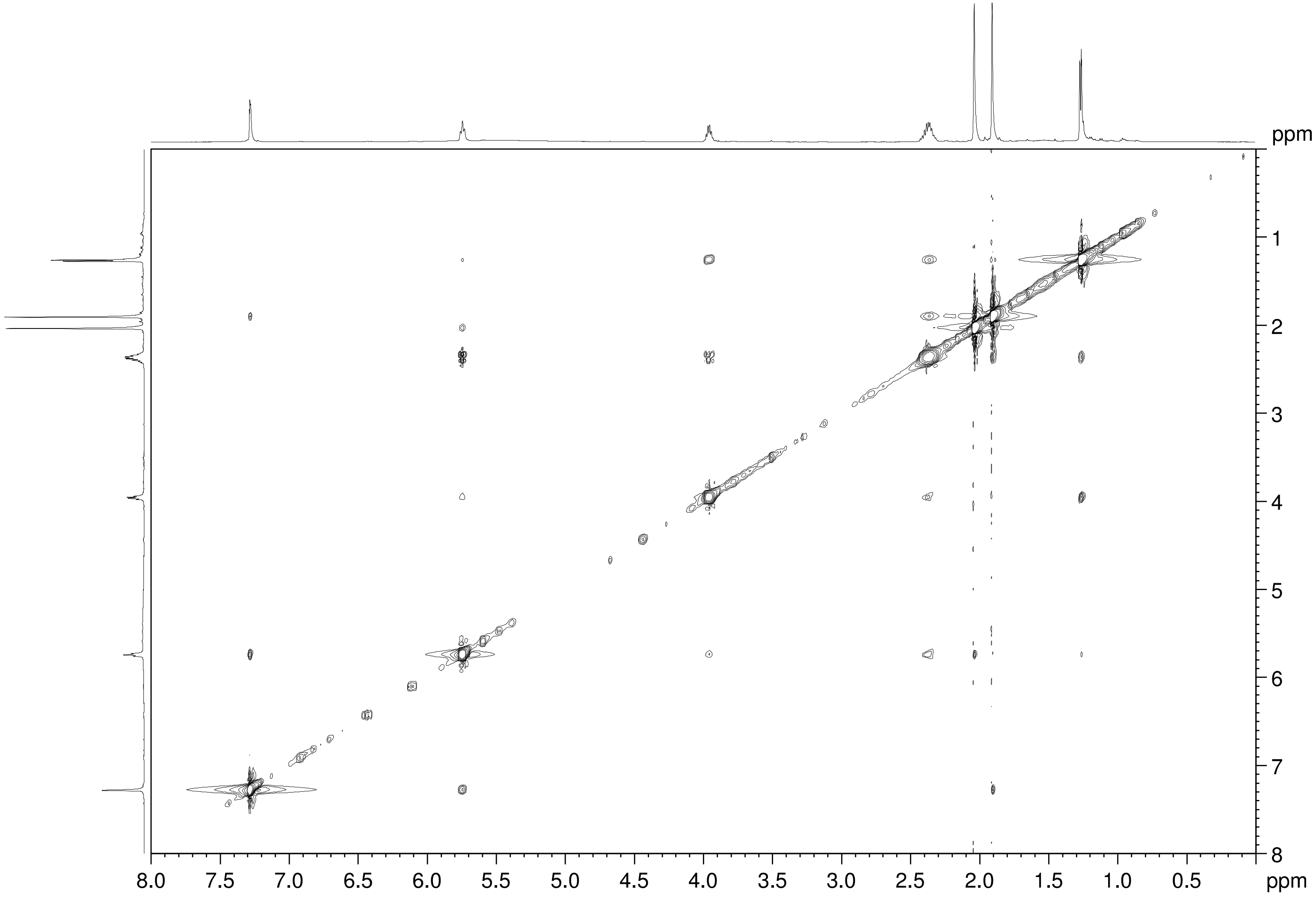

Figure S25. ^{1}H-^{1}H COSY spectrum of **4'a** (500 MHz, $CDCl_3$).

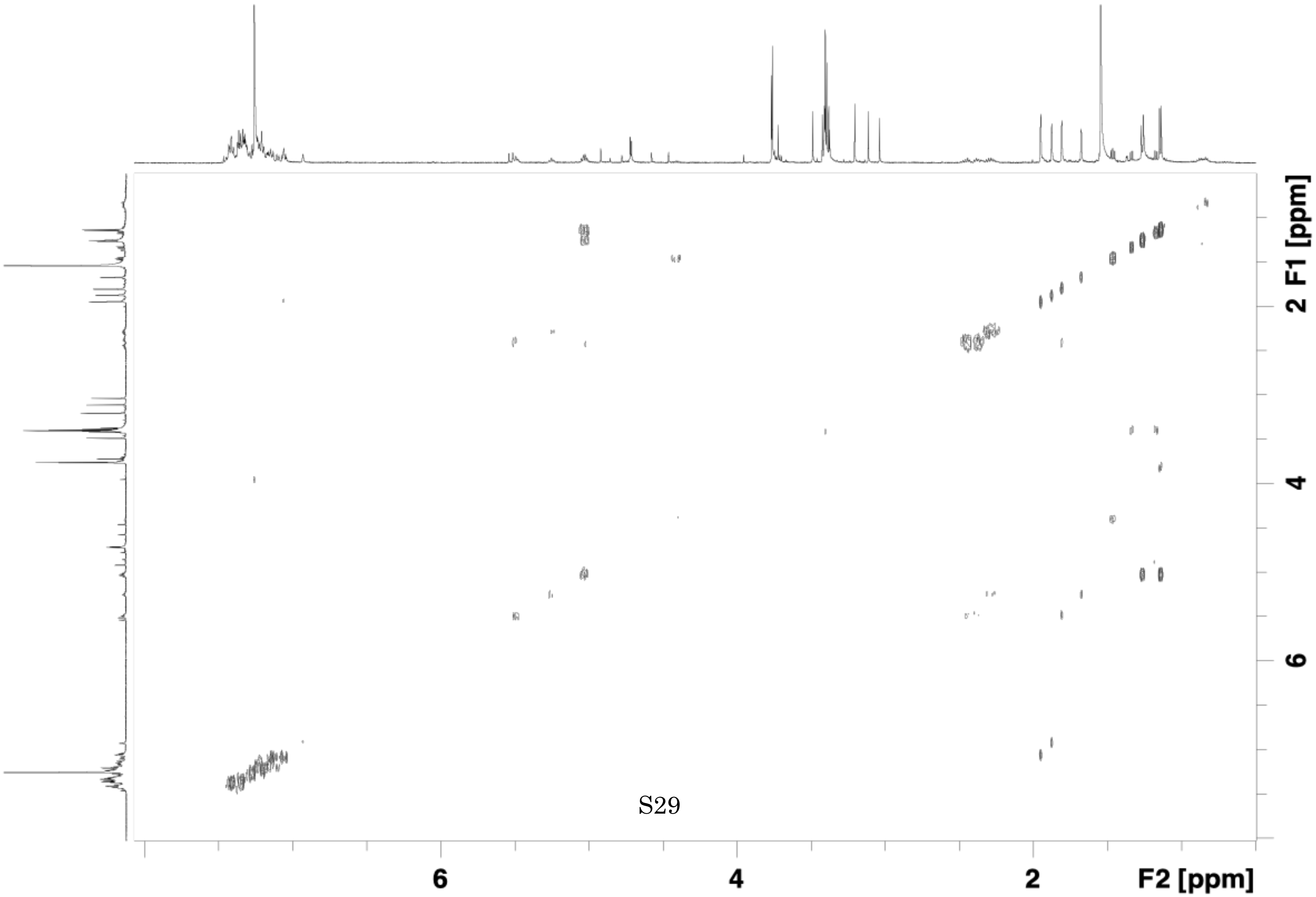

2 F1 [ppm]
4
6
6
4
2
F2 [ppm]

Figure S26. ^{1}H-^{1}H COSY spectrum of **4’b** (500 MHz, $CDCl_3$).

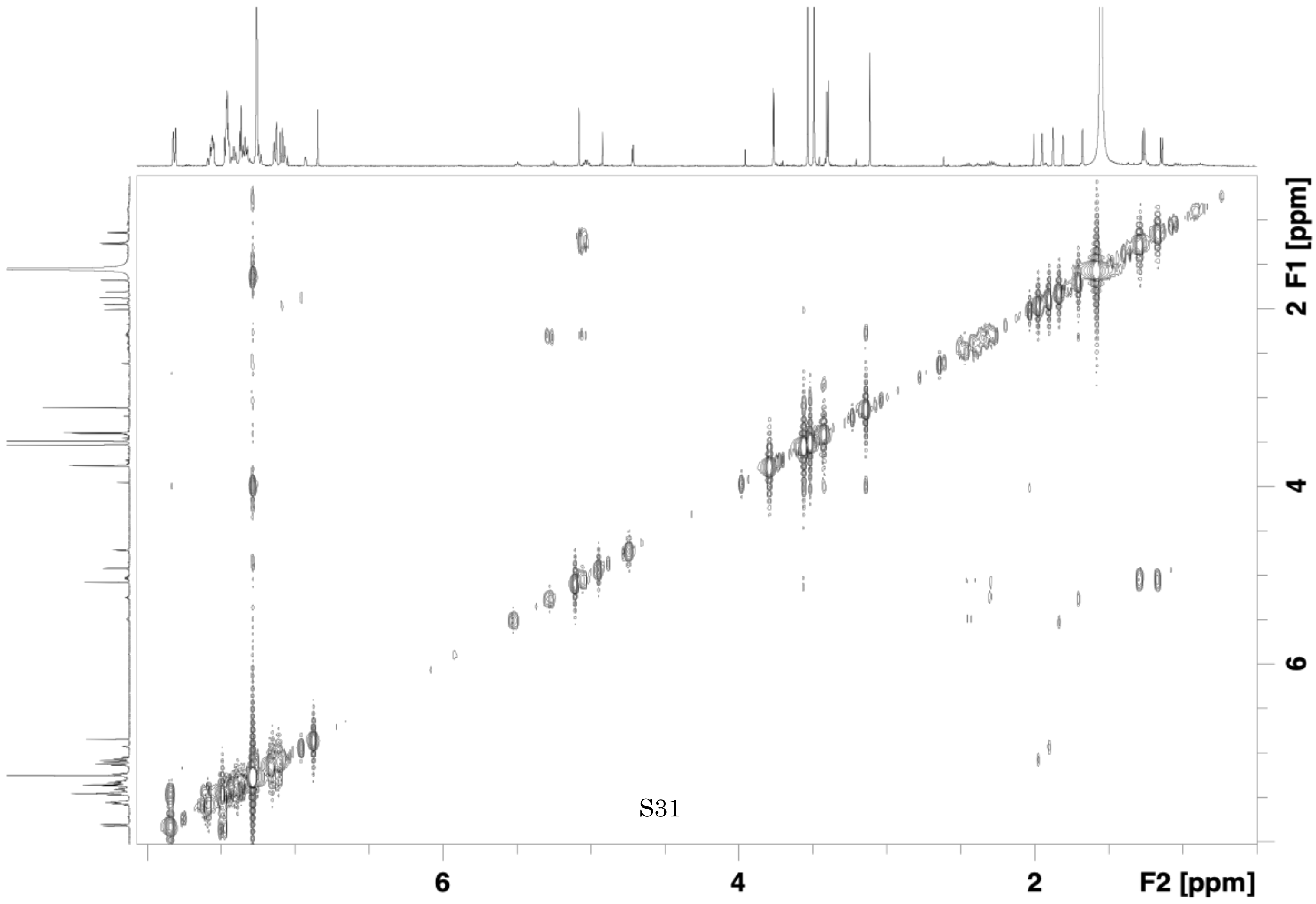

2 F1 [ppm]
4
6
6
4
2
F2 [ppm]

Figure S27. ^{1}H NMR spectrum of compound **5** (500 MHz, $CDCl_3$).

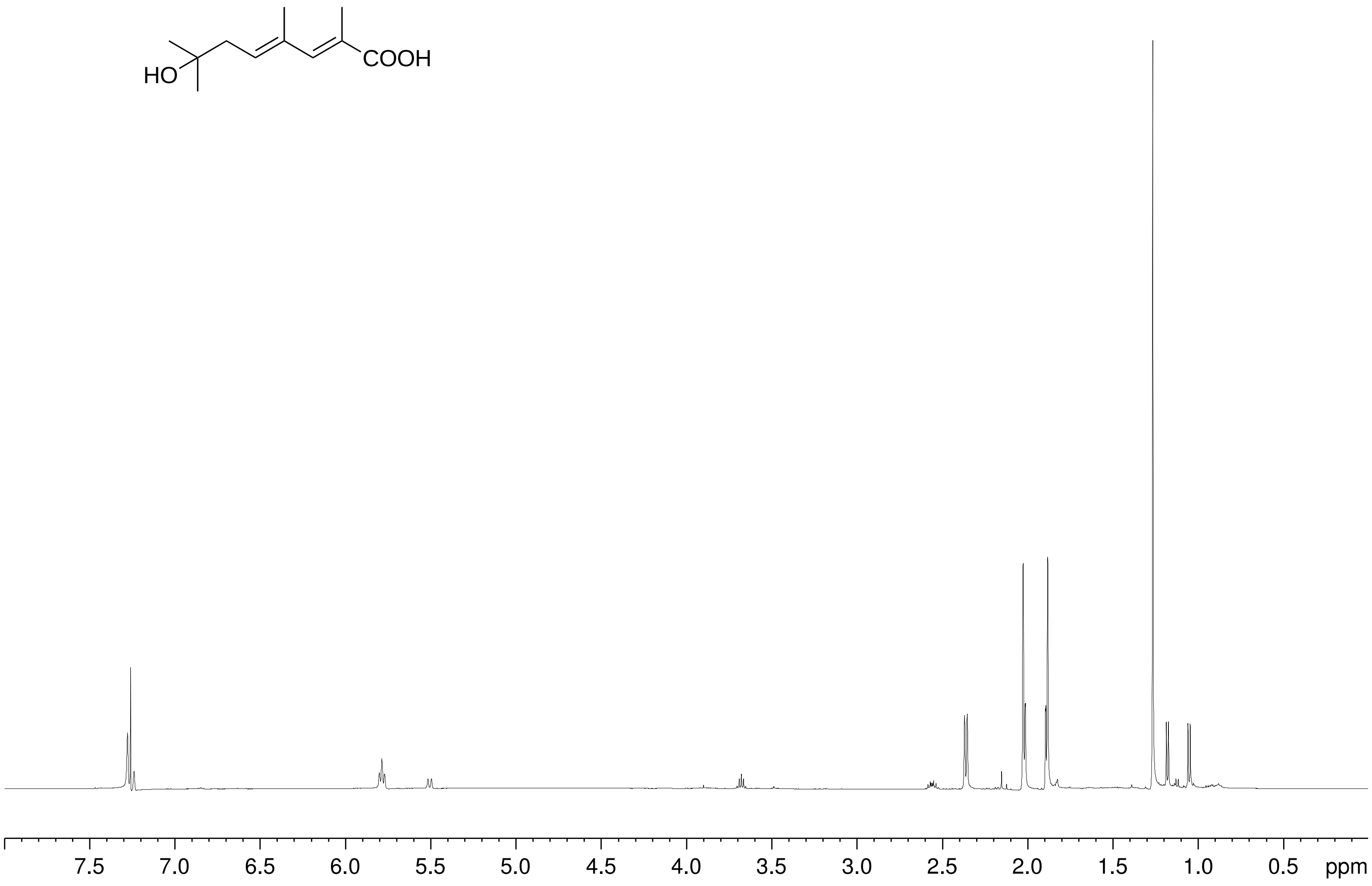

Figure S28. ^{13}C NMR spectrum of **5** (125 MHz, $CDCl_3$).

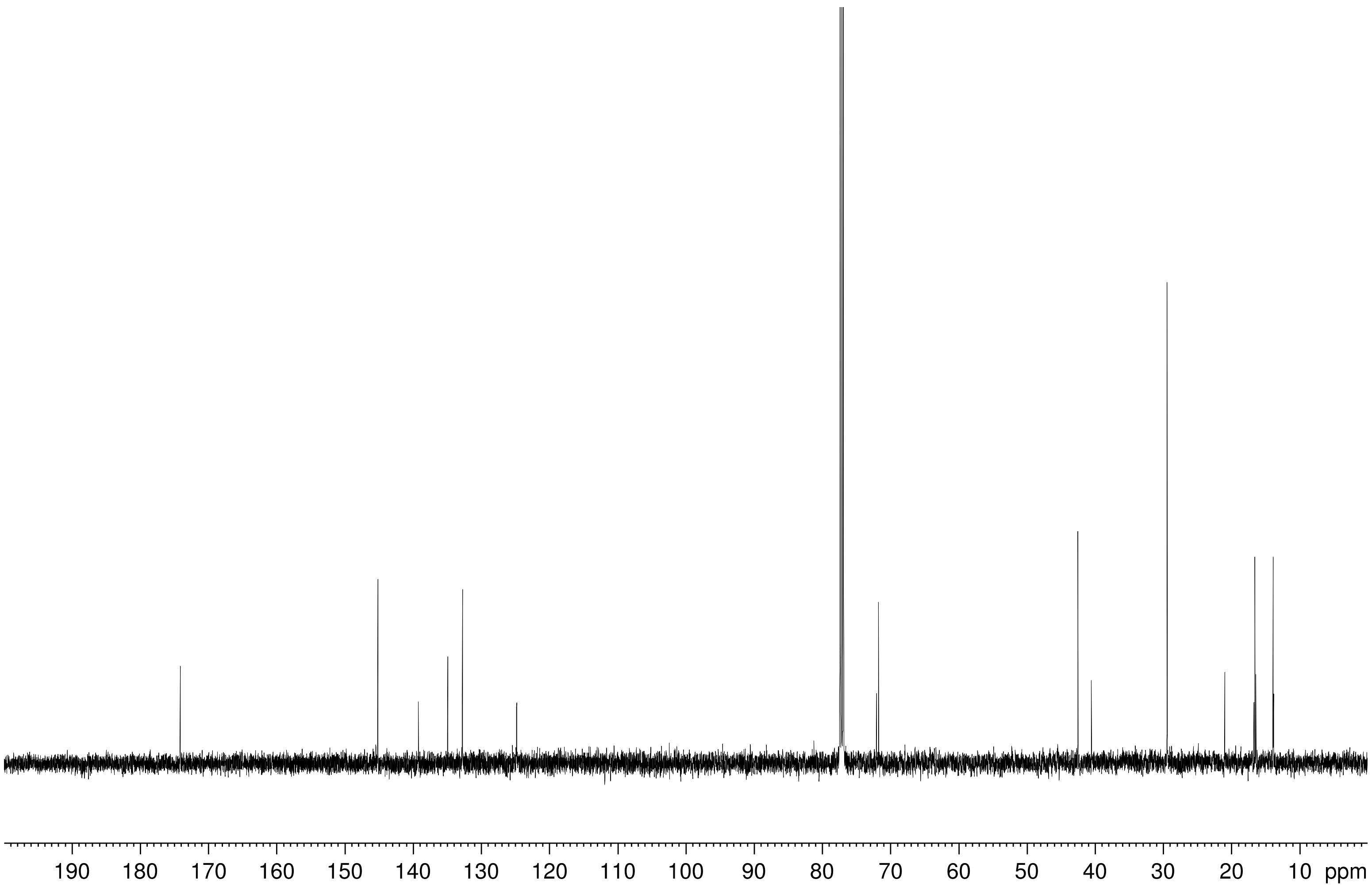

Figure S29. ^{1}H-^{1}H COSY spectrum of **5** (500 MHz, $CDCl_3$).

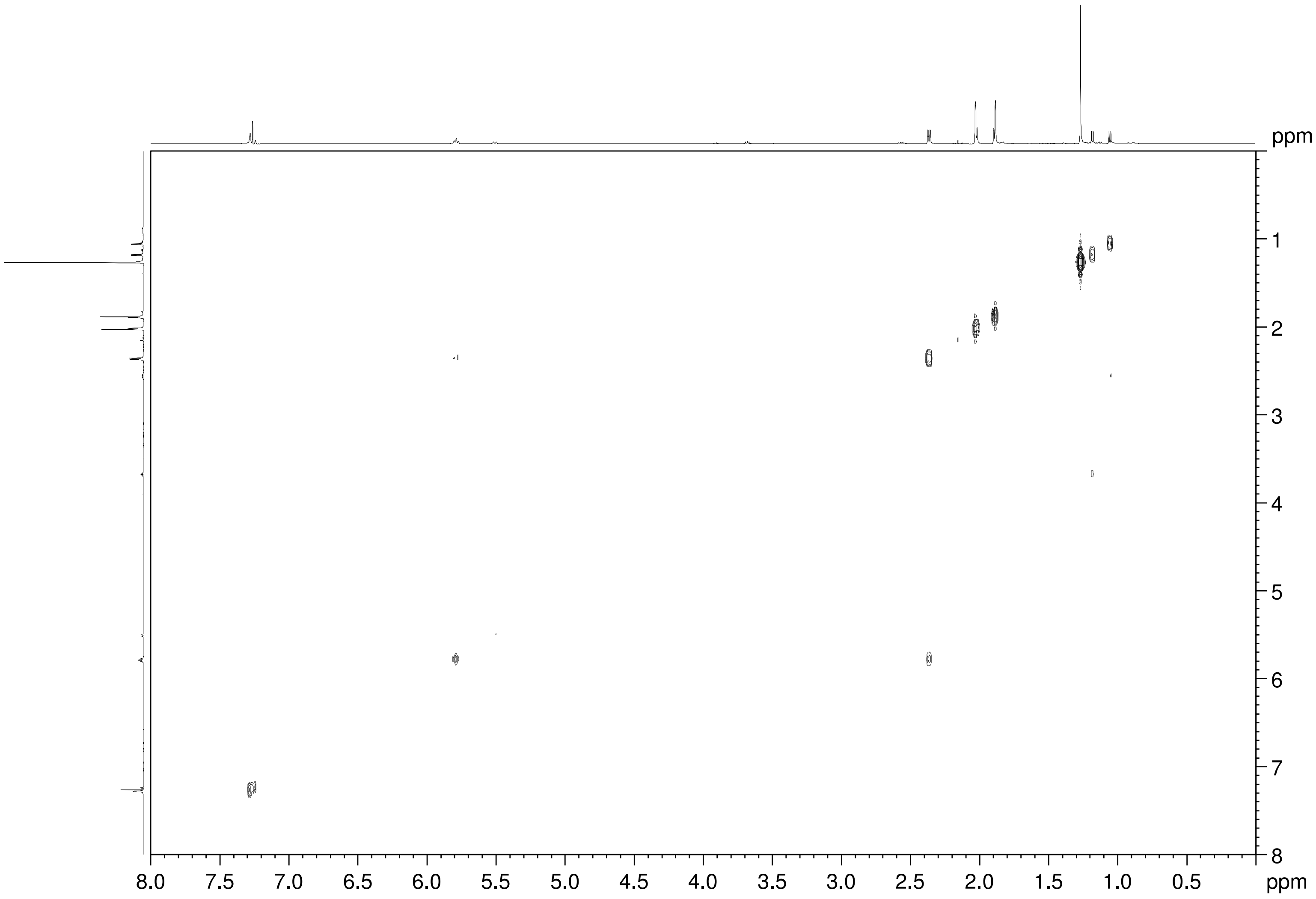

Figure S30. HSQC spectrum of **5** (500 MHz, $CDCl_3$).

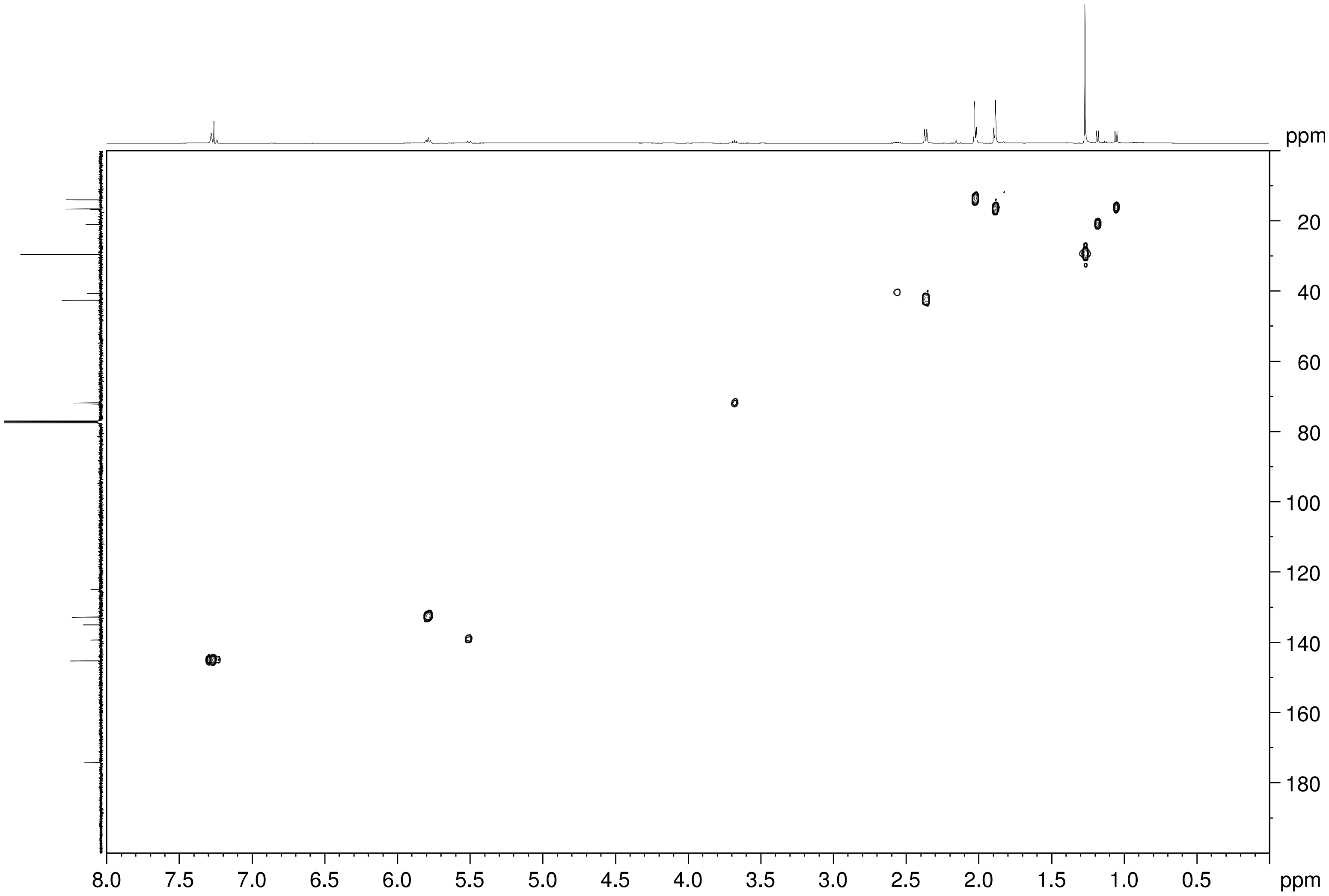

Figure S31. HMBC spectrum of **5** (500 MHz, $CDCl_3$).

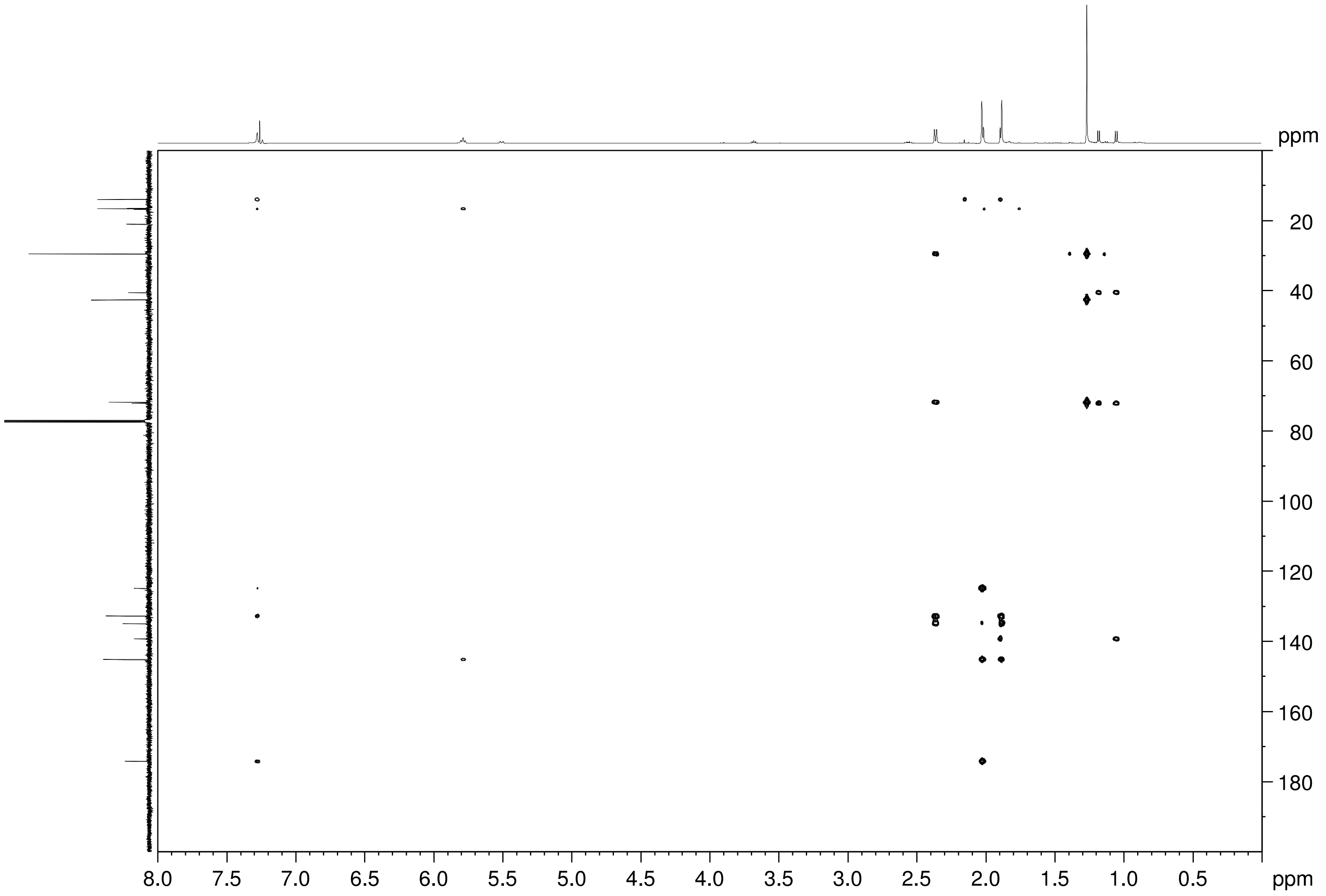

Figure S32. NOESY spectrum of **5** (500 MHz, $CDCl_3$).

Figure S33. ^{1}H NMR spectrum of compound **6** (500 MHz, $CDCl_3$).

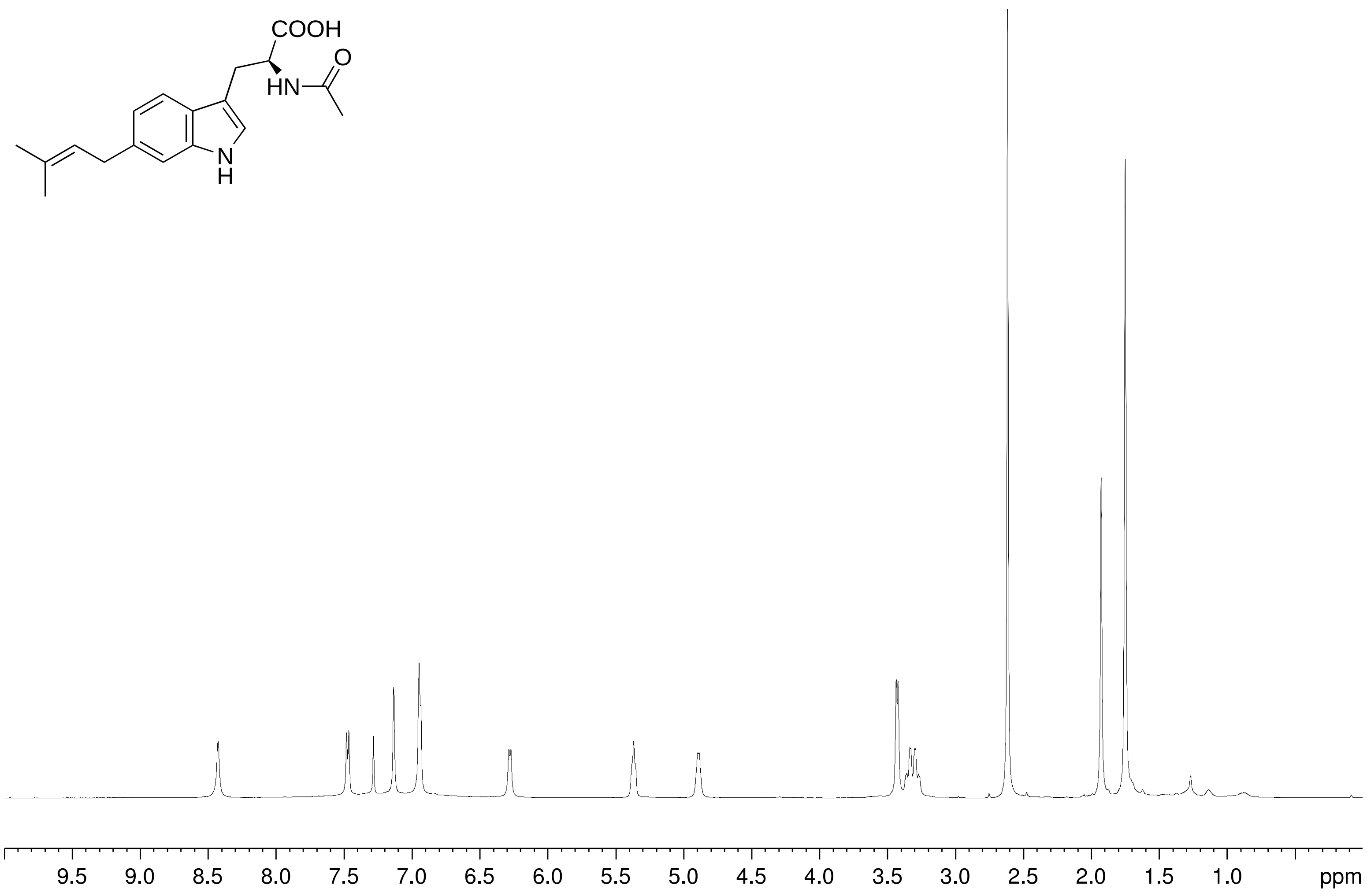

Figure S34. ^{13}C NMR spectrum of **6** (125 MHz, $CDCl_3$).

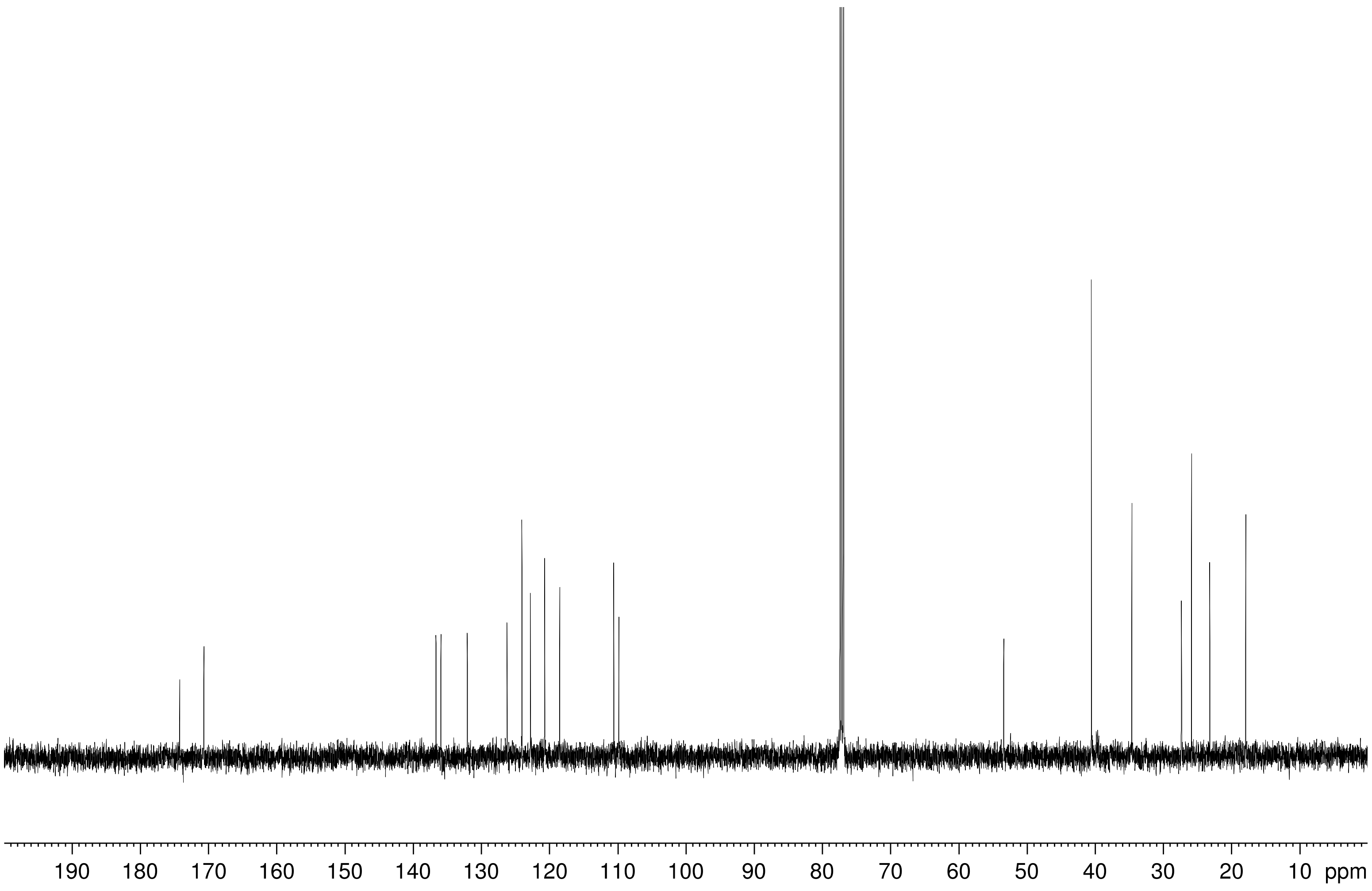

Figure S35. ^{1}H-^{1}H COSY spectrum of **6** (500 MHz, $CDCl_3$).

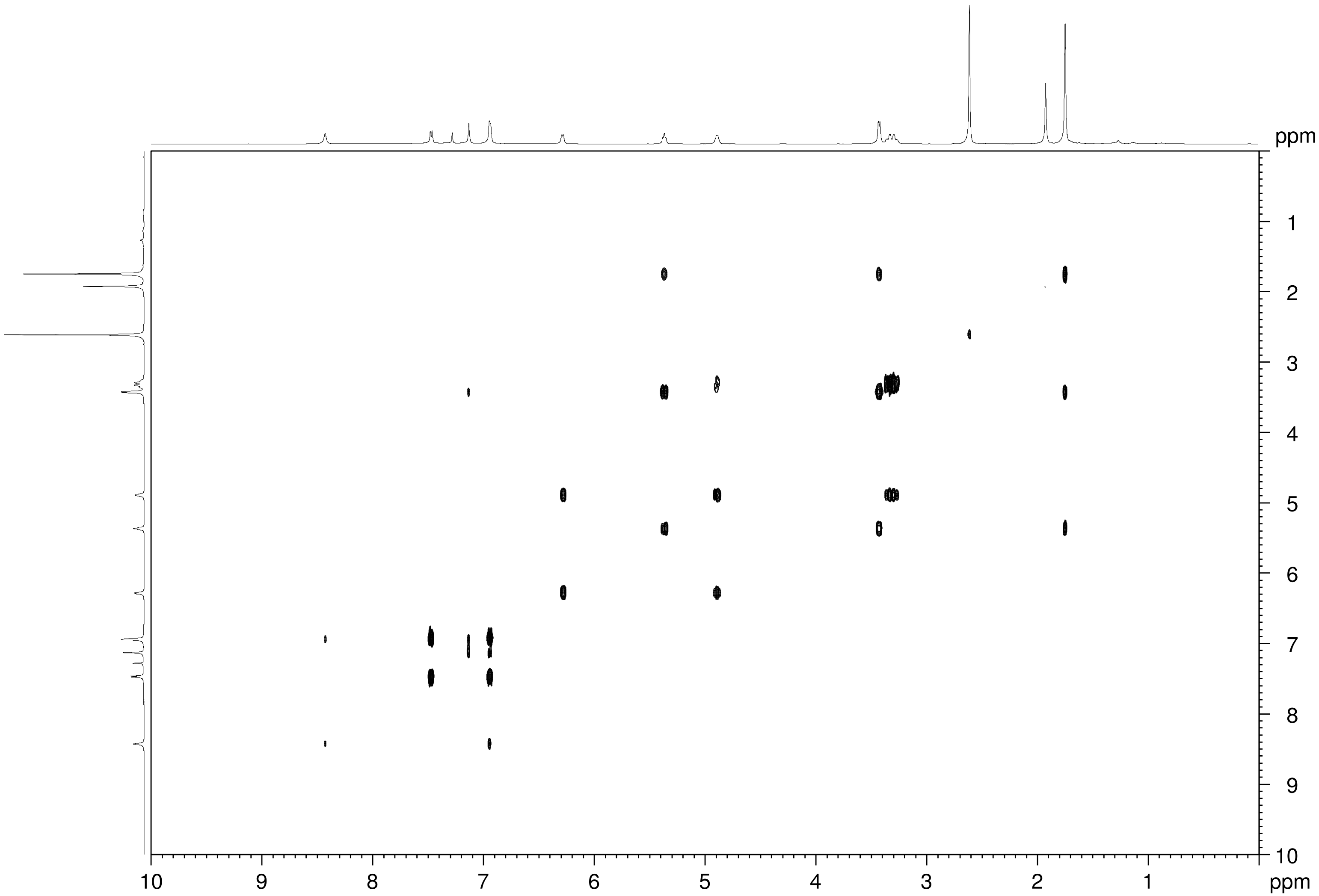

Figure S36. HSQC spectrum of **6** (500 MHz, $CDCl_3$).

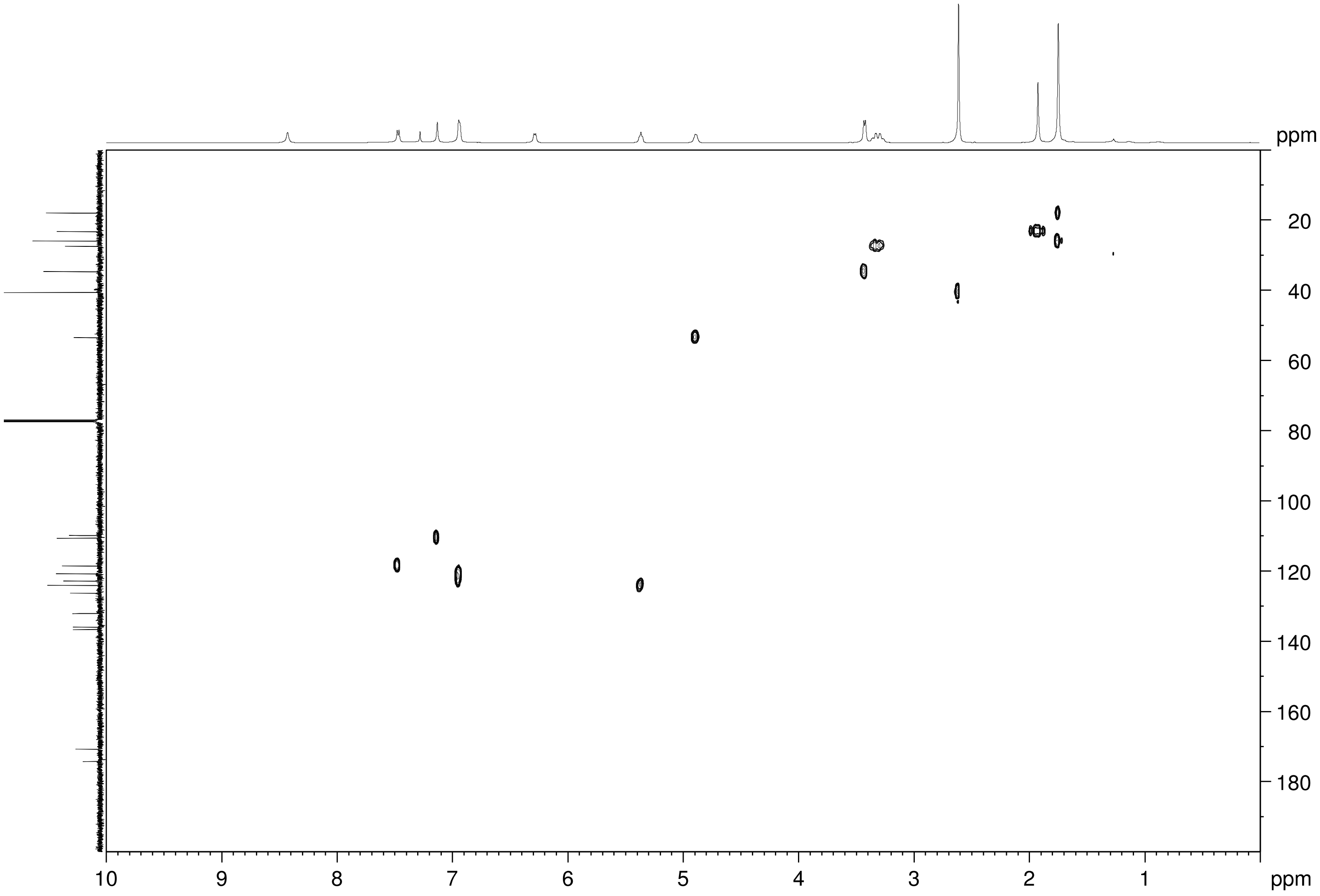

Figure S37. HMBC spectrum of **6** (500 MHz, $CDCl_3$).

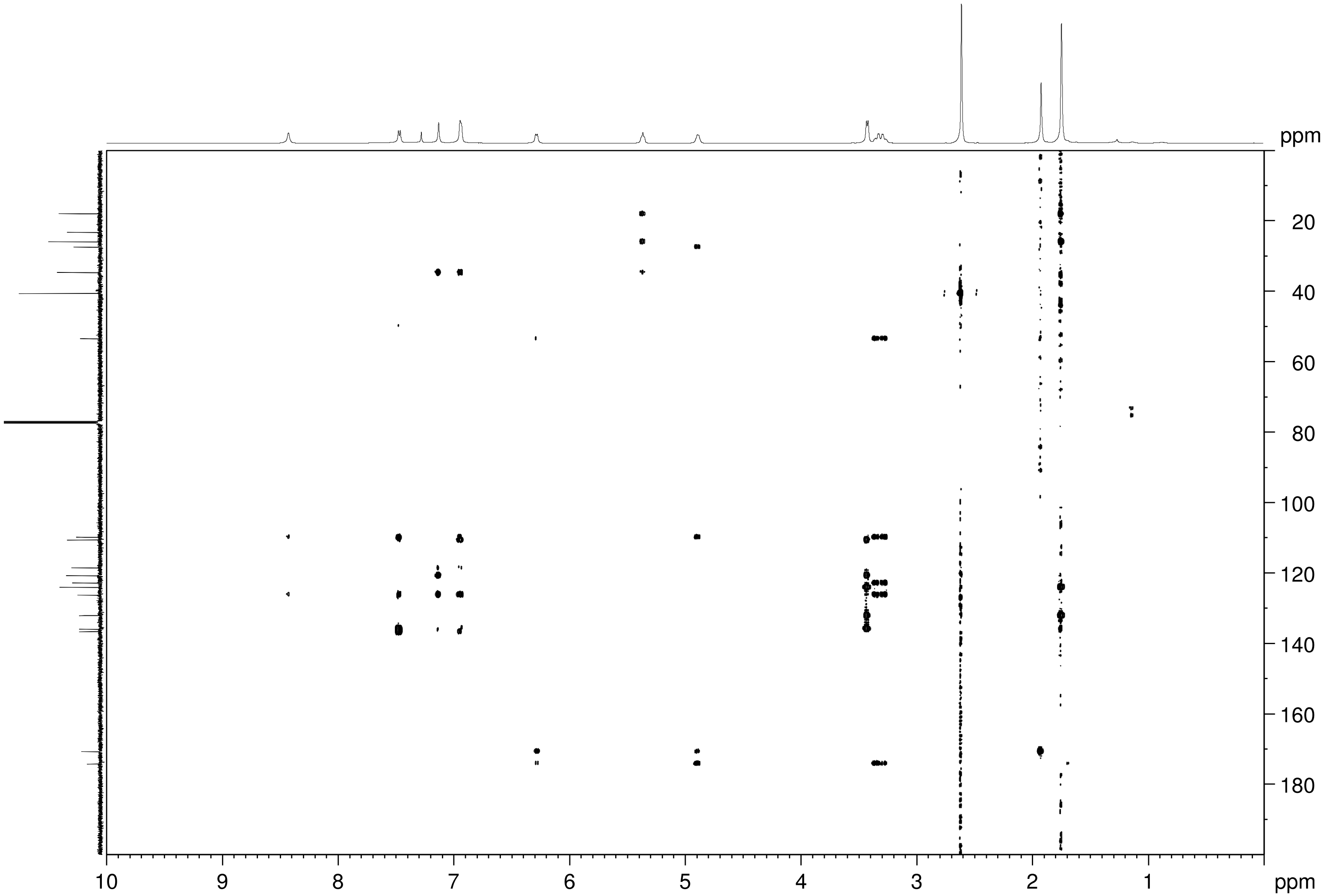

Figure S38. ^{1}H NMR spectrum of **6a** (500 MHz, DMSO-d_6).

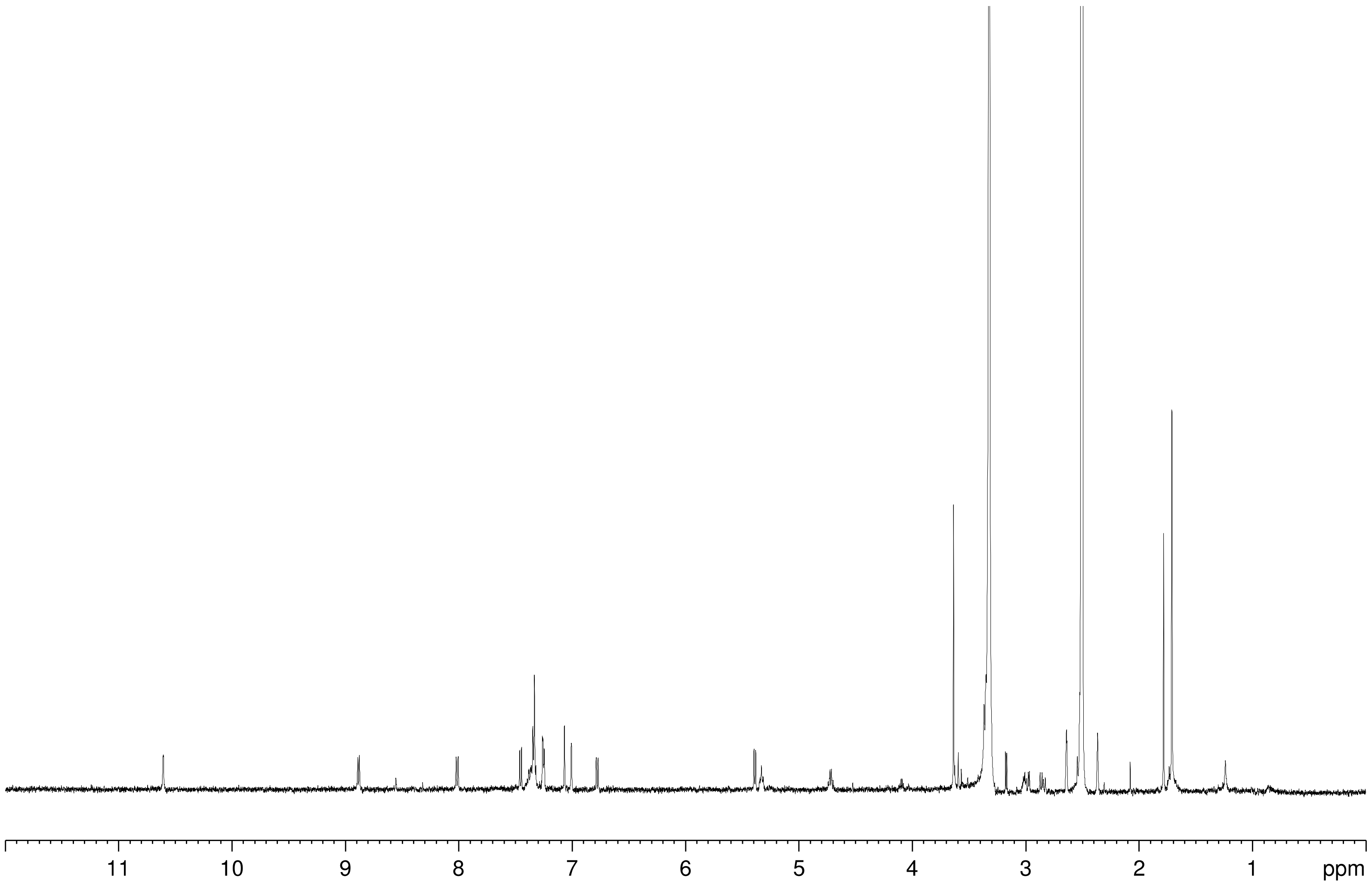

Figure S39. ^{1}H-^{1}H COSY spectrum of **6a** (500 MHz, DMSO-d_6).

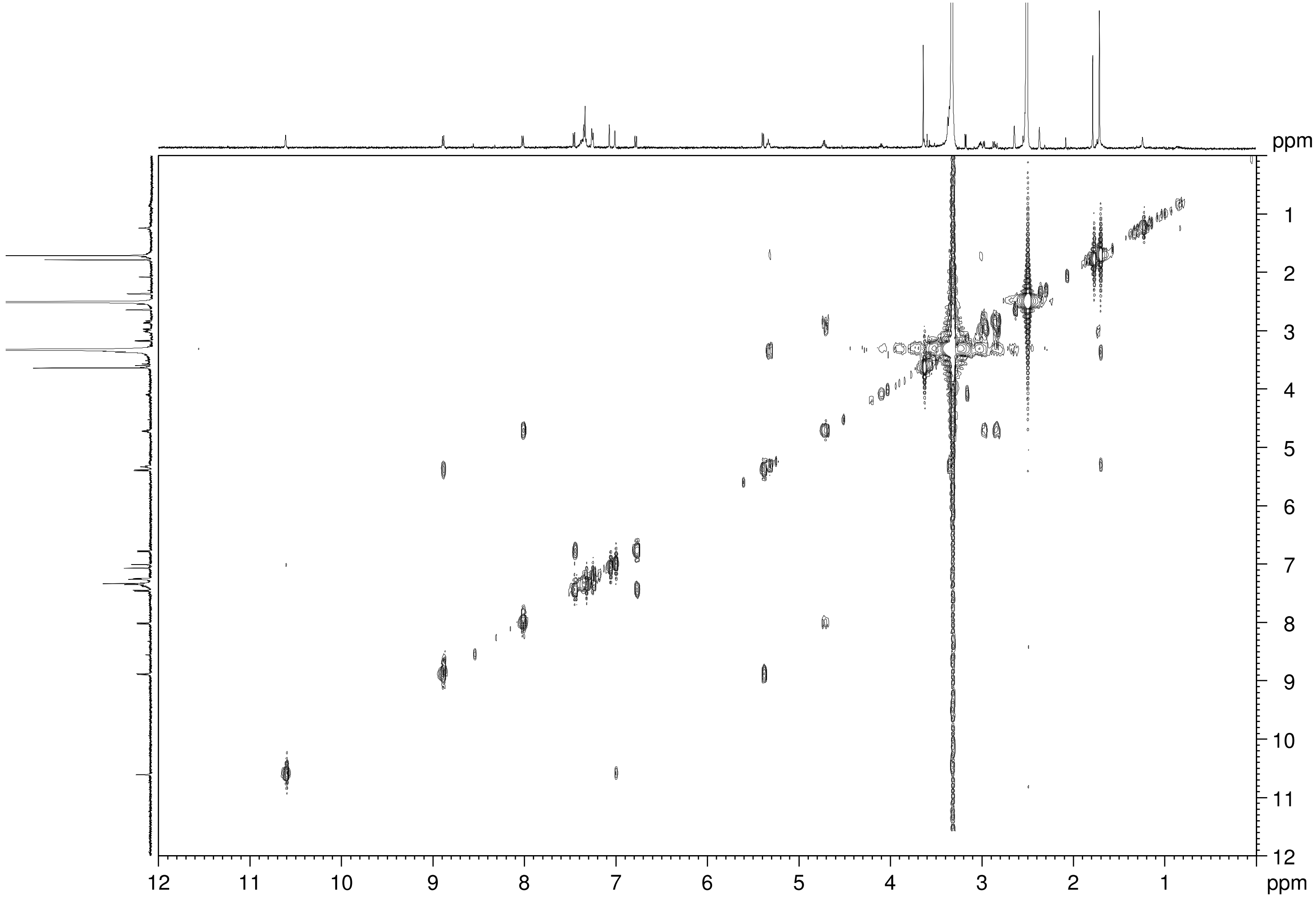

Figure S40. HSQC spectrum of **6a** (500 MHz, DMSO-d_6).

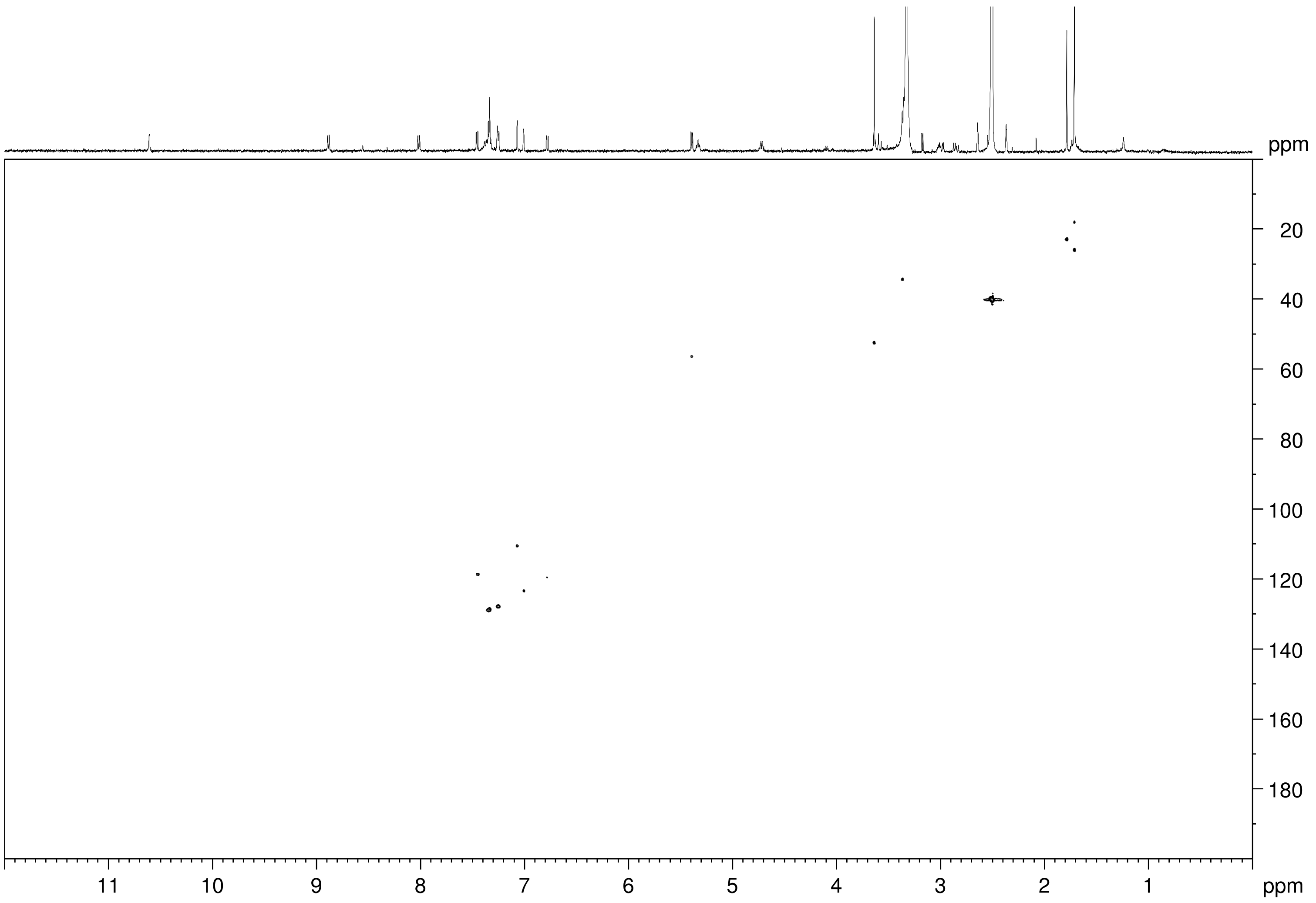

Figure S41. HMBC spectrum of **6a** (500 MHz, DMSO-d_6).

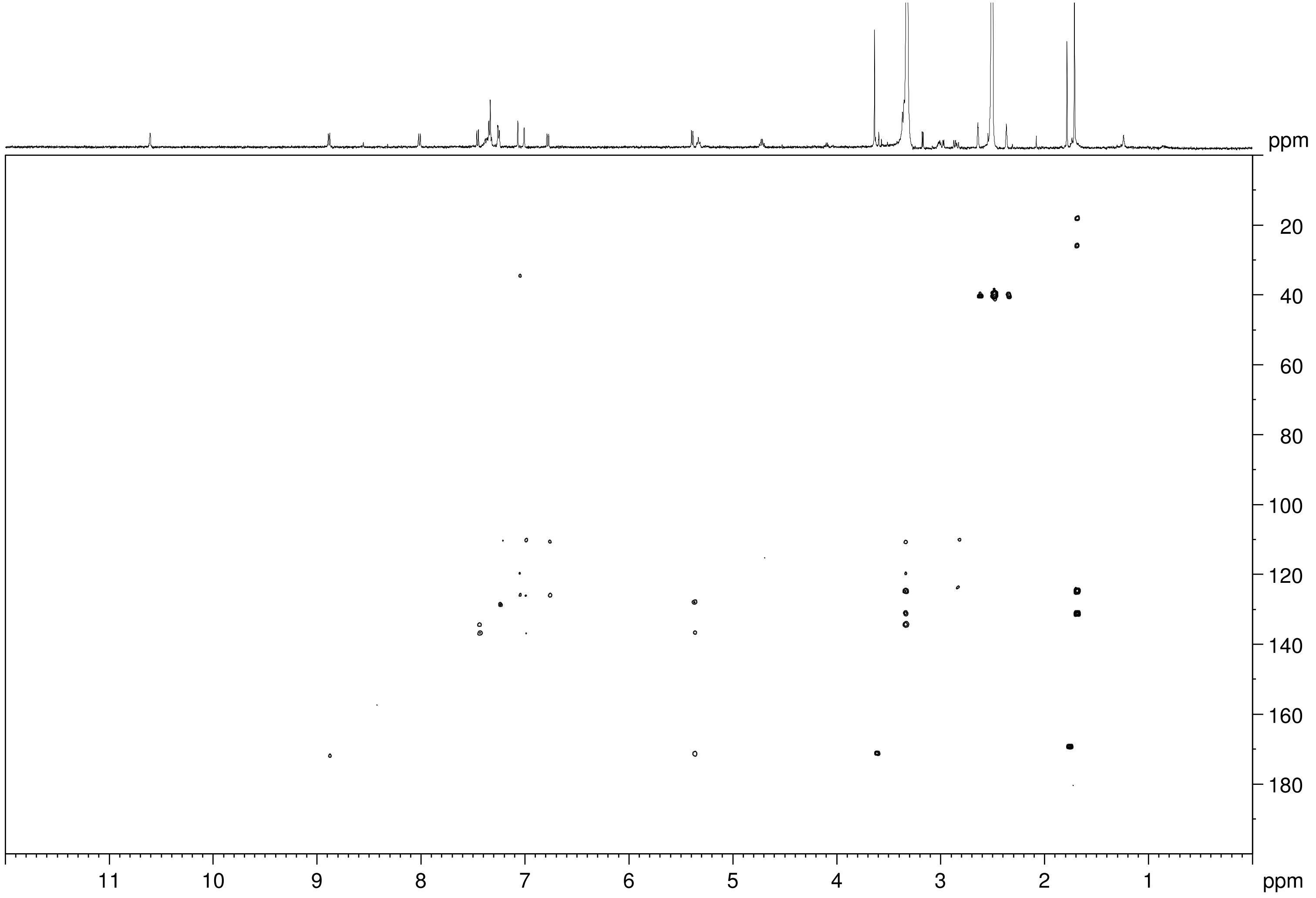

Figure S42. ^{1}H NMR spectrum of **6b** (500 MHz, DMSO-d_6).

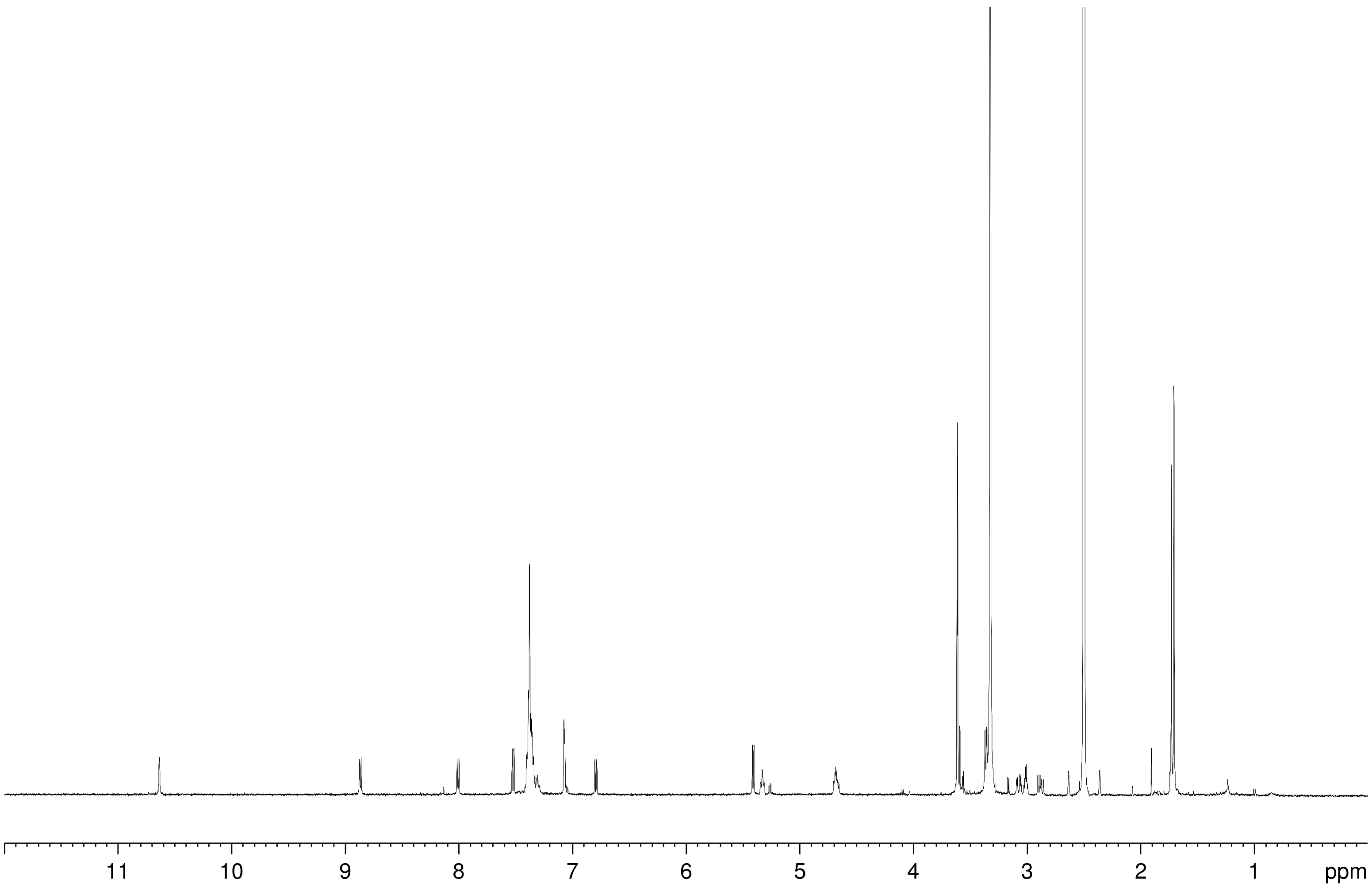

Figure S43. ^{1}H-^{1}H COSY spectrum of **6b** (500 MHz, DMSO-d_6).

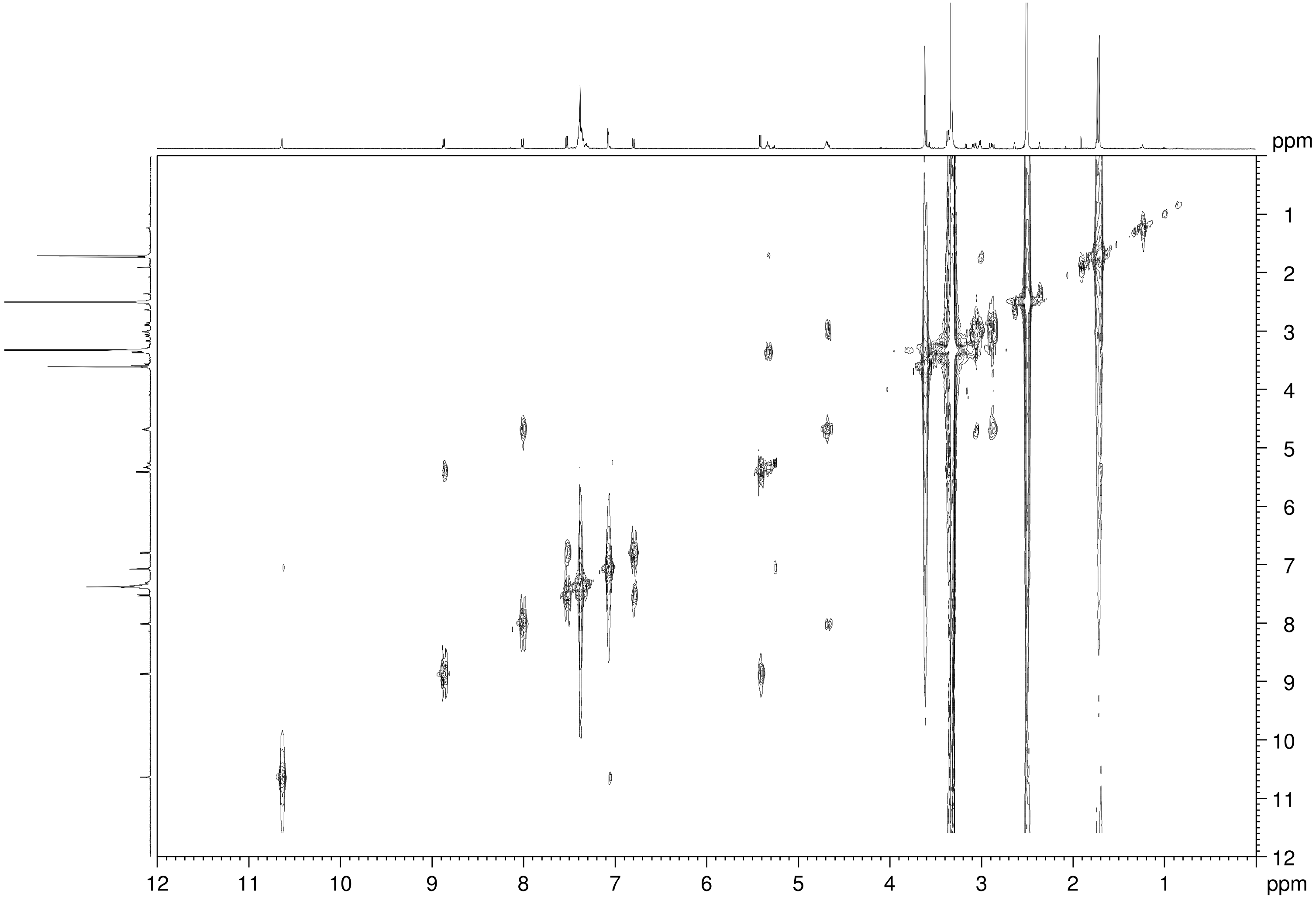

Figure S44. HSQC spectrum of **6b** (500 MHz, DMSO-d_6).

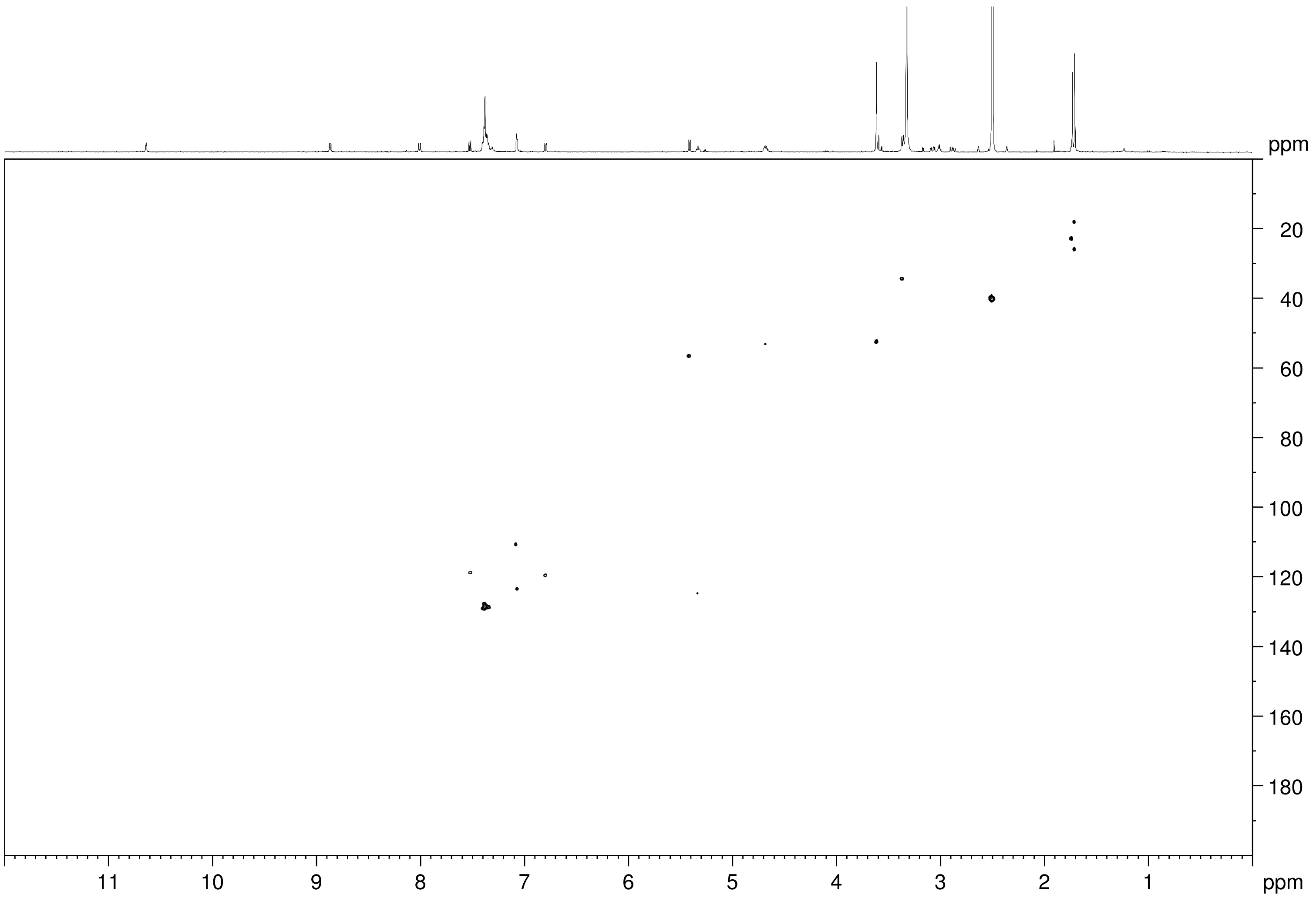

Figure S45. HMBC spectrum of **6b** (500 MHz, DMSO-d_6).

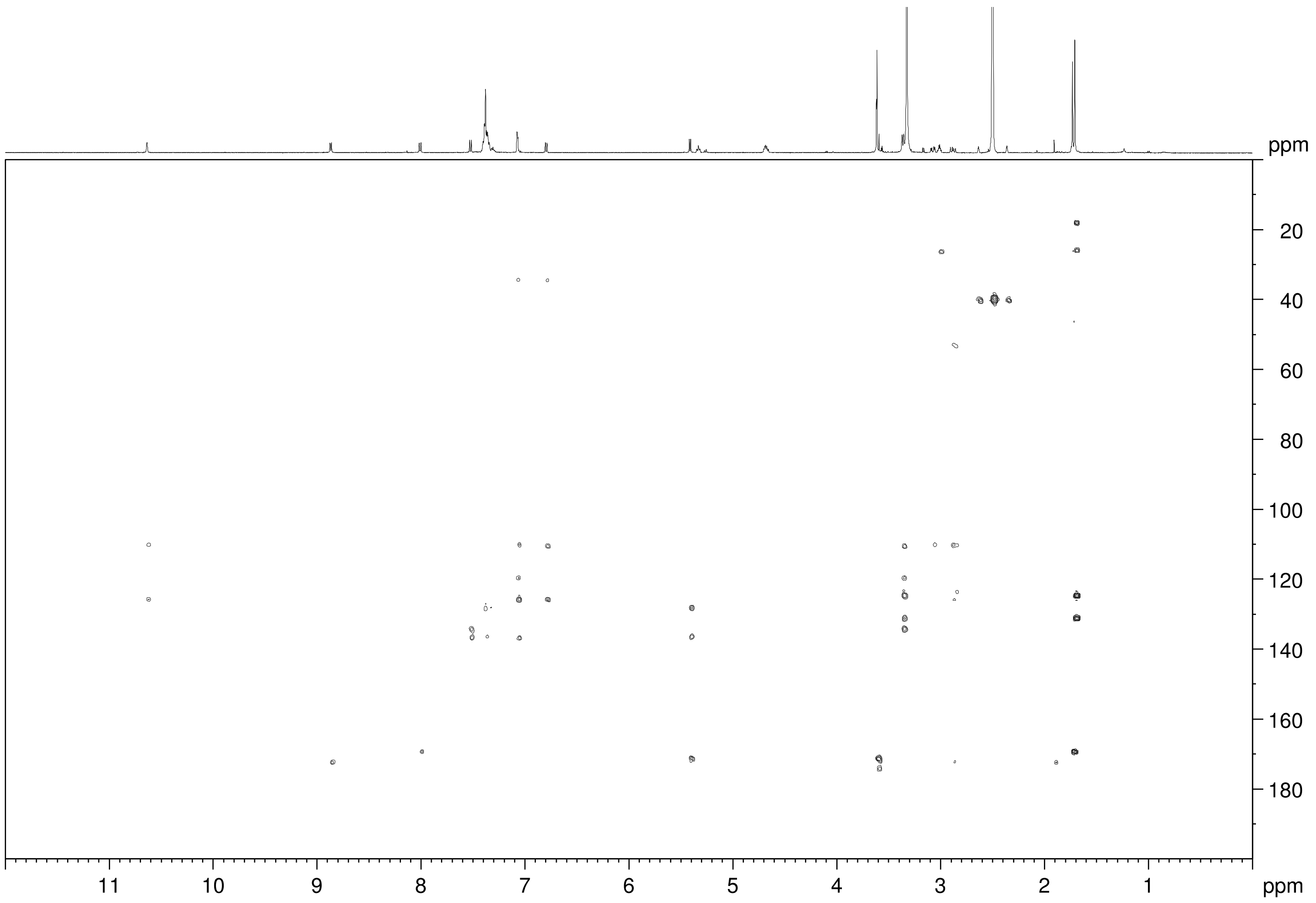

Supplement: File 1 — Copies of NMR spectra. [file Beilstein_J_Org_Chem-17-2939-s001.pdf]
